# Highly Diastereoselective Synthesis of Tetrahydroquinoline Derivatives *via* [4 + 2] Annulation of *Ortho*-Tosylaminophenyl-Substituted *Para-*Quinone Methides and Cyanoalkenes

**DOI:** 10.3389/fchem.2021.764866

**Published:** 2021-11-03

**Authors:** Taiwei Dong, Peifeng Wei, Min Li, Feng Gao, Yuan Qin

**Affiliations:** ^1^ College of Pharmacy, Shaanxi University of Chinese Medicine, Xianyang, China; ^2^ State Key Laboratory of Military Stomatology and National Clinical Research Center for Oral Diseases and Shaanxi Clinical Research Center for Oral Diseases, Department of Orthodontics, School of Stomatology, The Fourth Military Medical University, Xi’an, China

**Keywords:** tetrahydroquinolines, p-quinone methides, multi-substituted alkenes, Aza-Michael, 1, 6-conjugate addition

## Abstract

As a privileged structural motif, tetrahydroquinoline skeletons widely exist in biologically active natural products and pharmaceuticals. In this protocol, a highly diastereoselective [4 + 2] annulation of *ortho*-tosylaminophenyl-substituted *p*-QMs and cyanoalkenes to construct tetrahydroquinoline derivatives has been successfully achieved. This strategy proceeds efficiently under mild condition, offering straightforward route to a variety of 4-aryl-substituted tetrahydroquinolines with high yields, excellent diastereoselectivities, broad functional group tolerance as well as gram-scale capacity. Moreover, a one-pot reaction sequence utilizing *in situ* generated *p*-QMs under the similar condition to build tetrahydroquinoline framework is smoothly conducted with good reaction performance as well as step and atom economy.

## Introduction

**FIGURE 1:**
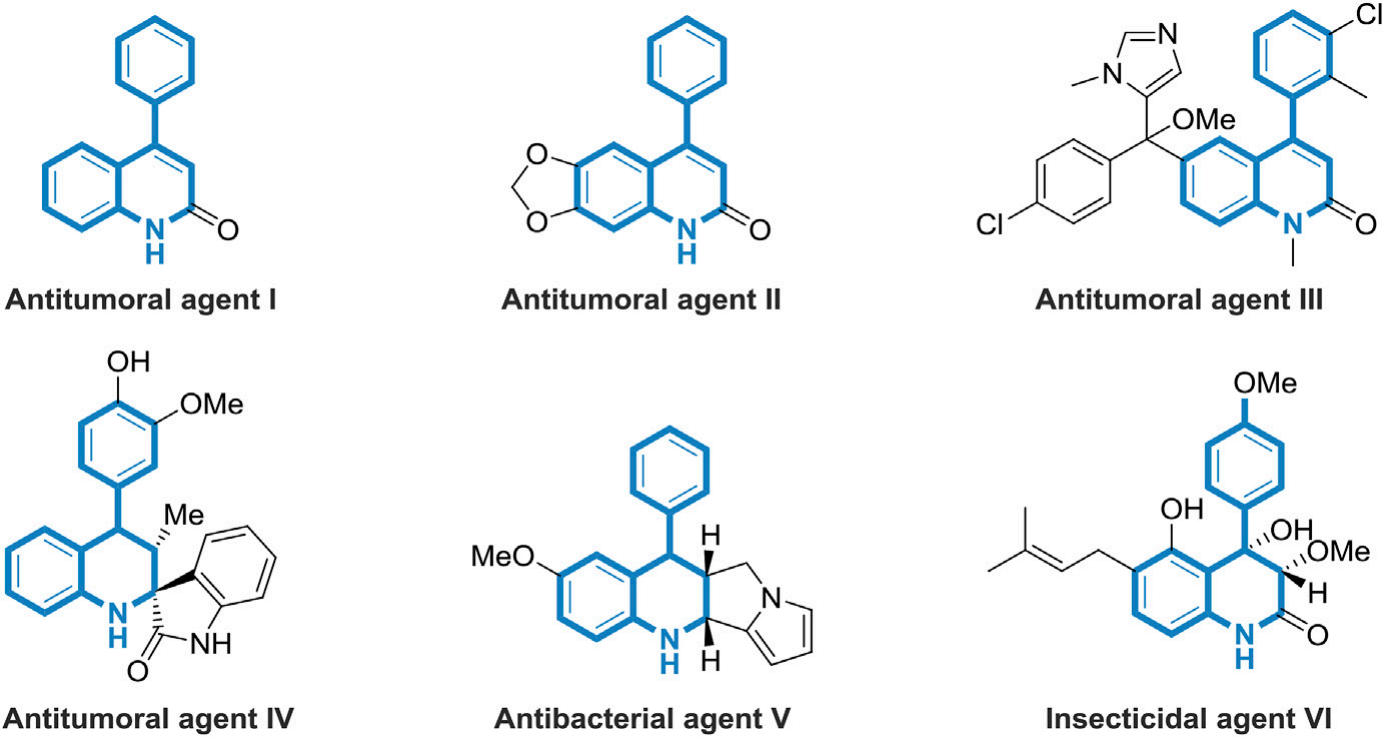
Selected natural products and synthetic compounds containing di- or tetra-hydroquinoline frameworks.

As privileged structural motifs, nitrogen-containing heterocycles widely exist in biologically active natural products and pharmaceuticals (Noolvi et al., 2011; Solomon and Lee, 2011; Afzal et al., 2015; Murlykina et al., 2018; Harikandei et al., 2019; Staskiewicz et al., 2021). Among them, 4-phenyl-substituted tetrahydroquinolines are of great importance owing to their wide range of applications in medicinal chemistry, exhibiting antitumor and antibacterial properties (Figure 1). For example, 4-phenylquinolin-2 (1H)-one I shows potential as a specific allosteric inhibitor of Akt (Huang et al., 2017). Compound II has been designed as novel anticancer agents that induce apoptosis with cell cycle arrest at G2/M phase (Chen et al., 2013). As a rationally developed antitumoral agent, compound III displays excellent bioactivity to kill parasite 14DM (Kraus et al., 2009). Known as a novel synthetic molecule, compound IV exhibits antitumoral and antiplasmodial activities (Vladimir et al., 2010). The well-designed compound V owns good antibacterial activity against microorganisms of *Escherichia coli* (Ramesh et al., 2009). Peniprequinolone VI, isolated from *Penicillium* sp. FKI-2140, demonstrates impressive insecticidal activity (Uchida et al., 2006). Thus, considering the significant research value of tetrahydroquinolines derivatives in medicinal chemistry, the development of efficient and facile methods to build these valuable skeletons is highly demanded.

**Scheme 1:**
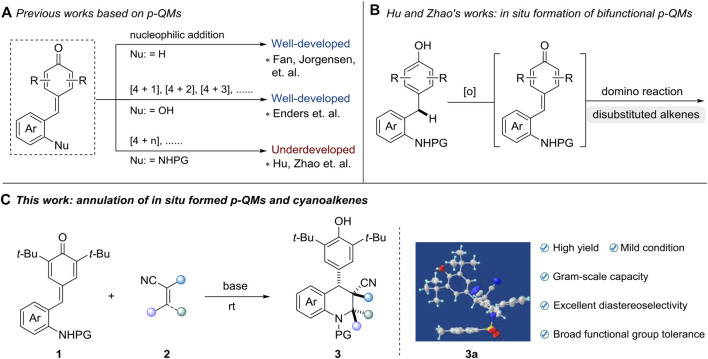
Advancements based on functional *p*-QMs **(A, B)** and our strategy for synthesis of multi-substituted tetrahydroquinolines **(C)**.

Owing to the remarkable chemical reactivity comprising reactive carbonyl and olefinic moieties, *p*-quinone methides (*p*-QMs) have been considered versatile building blocks in organic synthesis (Toteva and Richard, 2011; Parra and Tortosa, 2015; Chauhan et al., 2017; Lima et al., 2020; Wang J.-Y. et al., 2020). Inspired by the seminal works of Fan and Jorgensen (Chu et al., 2013; Caruana et al., 2014), numerous of methods utilizing *p*-QMs as vinylogous Michael acceptors have been successfully developed through 1,6-conjugate addition and annulation reactions (Lou et al., 2015; Wang et al., 2015; Deng et al., 2016; Dong et al., 2016; He et al., 2016; Li et al., 2016; Ma et al., 2016; Zhang et al., 2016; Roiser and Waser, 2017; Zhang et al., 2017). Recently, Enders and co-workers first demonstrated the potential of *ortho*-hydroxyphenyl-substituted *p*-QMs in [4 + 2] cyclization reaction (Zhao et al., 2016), which subsequently enables the extensively investigation of [4 + 1] (Chen et al., 2018; Liu L. et al., 2018; Xiong et al., 2018; Zhi et al., 2018; Zhou et al., 2018; Lu et al., 2019; Tan et al., 2019) [4 + 2] (Jiang X. L. et al., 2018; Mei et al., 2018; Zhang et al., 2018; Yang et al., 2019; Zhang et al., 2019; Huang et al., 2020; Roy et al., 2020; Tan et al., 2020; You et al., 2020) and [4 + 3] (Jiang F. et al., 2018; Li et al., 2018; Liu Q. et al., 2018; Chen et al., 2019) annulations by various research groups. Although great progress has been witnessed in this field, the employment of *p*-QMs substrates in the construction of heterocyclic frameworks, especially nitrogen-containing heterocyclic frameworks, still remains underdeveloped (Scheme 1A). Very recently, the group of Hu and Zhao pioneered the design of *in situ* generated *ortho*-tosylaminophenyl-substituted *p*-QMs and transformations of this class of substrates in [4 + 1] (Wang et al., 2019; Wang et al., 2020a) and [4 + 2] (Wang et al., 2018; Si et al., 2020) annulation reactions, providing a straightforward access to construct valuable tetrahydroquinoline and 2,3-dihydroindole derivatives, respectively (Scheme 1B). Those transformations avoid the utilization of presynthesized *p*-QMs, which greatly enhance the step and atom economy of this strategy. To date, currently limited reports almost concentrate on applications between *ortho*-tosylaminophenyl-substituted *p*-QMs and disubstituted alkenes. However, rare explorations regarding multifunctional alkenes like tri- or tetra-substituted alkenes, which contain large steric hindrance or relatively poor reactivity, have been successfully achieved so far (Wang et al., 2020b). Hence, to address the aforementioned challenges, we herein reported a highly diastereoselective [4 + 2] annulation of *ortho*-tosylaminophenyl-substituted *p*-QMs and cyanoalkenes for the efficient synthesis of 4-aryl-substituted tetrahydroquinolines under mild conditions. This reaction features high yield (up to 96% yield), excellent diastereoselectiveties (>20:1 dr), broad functional group tolerance as well as gram-scale capacity (Scheme 1C).

## Results and Discussion

To verify the feasibility of our protocol, a serial of reaction parameters was investigated to explore the best condition for the [4 + 2] annulation. Initially, we established the model reaction using *ortho*-tosylaminophenyl-*p*-QMs **1a** and α,α-dicyanoalkenes **2a** as substrates (Table 1). To our delight, with the help of Cs_2_CO_3_ as base, the desired product **3a** was successfully obtained in DCM at room temperature in 80% yield with excellent diastereoselectivity of >20:1 (Table 1, entry 1). Encouraged by this promising result, different bases were systematically evaluated, and we found that Na_2_CO_3_, pyrolidine, and triethylamine (TEA) were inefficient for this reaction compared to Cs_2_CO_3_ (entries 2–4). Delightfully, the organic base 1,8-diazabicyclo [5.4.0]undec-7-ene (DBU) was beneficial to this transformation, generating product **3a** in 89% yield (entry 5). Then, we switched attention to solvents screening to further improve reaction efficiency (entries 6–10), and the toluene serving as reaction mediate performed best with up to 96% yield (entry 8). After the confirmation of the optimized base and solvent, the factors of temperature and substrate ratio in this base-mediated catalyst-free protocol was subsequently investigated, and it was found that none of improvement of reaction efficiency was observed when applying other temperature or substrate ratio (entries 11–14). Furthermore, we found that reducing the DBU loading to 0.01 mmol and raising the reaction temperature led to slight decrease in yield with prolonged reaction time (entries 15–16).

**TABLE 1:** Optimization of [4 + 2] annulation conditions.^a^ 
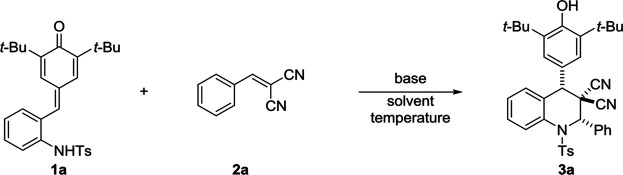

Entry	Base	Solvent	Yield^b^ (%)
1	Cs_2_CO_3_	DCM	80
2	Na_2_CO_3_	DCM	<10
3	Pyrrolidine	DCM	59
4	TEA	DCM	74
5	DBU	DCM	89
6	DBU	MeCN	80
7	DBU	THF	82
8	DBU	Toluene	96
9	DBU	Ethyl Acetate	91
10	DBU	1,4-Dioxane	84
11^c^	DBU	Toluene	96
12^d^	DBU	Toluene	93
13^e^	DBU	Toluene	96
14^f^	DBU	Toluene	90
15^g^	DBU	Toluene	89
16^h^	DBU	Toluene	86

a Unless other noted, all reactions were conducted with 1a (0.1 mmol), 2a (0.12 mmol) and base (0.02 mmol) in anhydrous solvent (2 ml) at room temperature for 1 h.

b Determined by ^1^H NMR using bromoform as an internal standard; dr > 20:1.

c The reaction was performed at 0°C for 3 h;

d The reaction was performed at 40°C for 30 min.

e 0.15 mmol 2a was added.

f 0.15 mmol 1a was added.

g 0.01 mmol base was added.

h 0.01 mmol base was added at the temperature of 80°C.

**Scheme 2:**
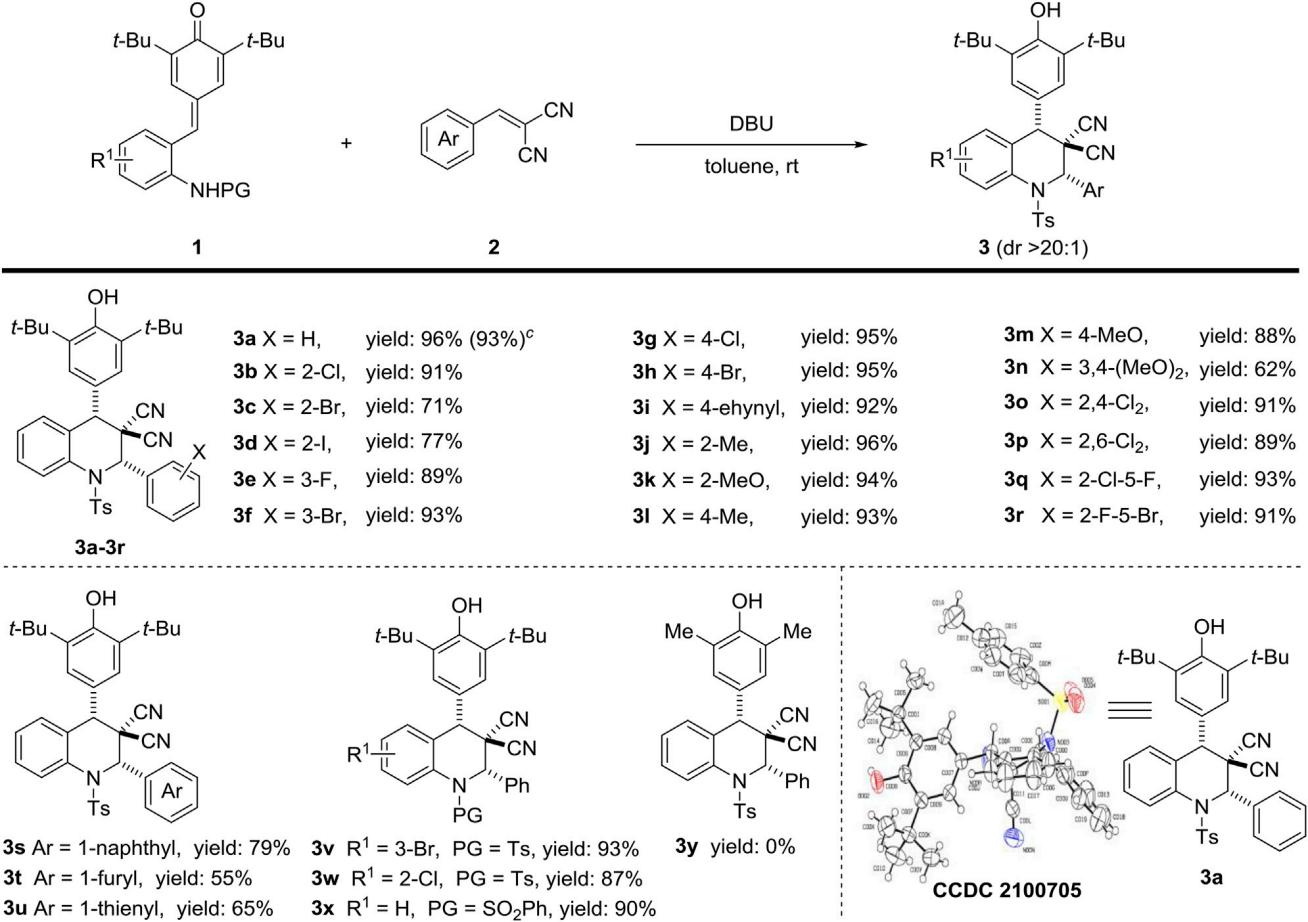
Investigation of the substrate scope of the [4 + 2] annulation. ^*a,b*^. ^*a*^ Unless other noted, the reaction was carried out with 1a (46.36 mg, 0.1 mmol), 2a (18.5 mg, 0.12 mmol) and base (0.02 mmol) in anhydrous solvent (2 ml) at room temperature for 1 h^*b*^ Yields are those of isolated products 3 after column chromatography; the diastereomeric ratio was determined by ^1^H NMR, dr > 20:1. ^*c*^ Gram scale reaction of 1a (1.02 g, 2.2 mmol) in toluene (5 ml) was conducted under the standard condition.

With the optimal reaction conditions in hand (Table 1, entry 8), we started to explore the substrate scope of this [4 + 2] annulation reaction (Scheme 2). Firstly, the scope of the α, *a*-dicyanoalkenes part was examined, and we were pleased to find that this protocol tolerated a wide range of α, *a*-dicyanoalkenes 2, which could readily react with 1a to afford 3a–3u in 55–96% yields. In detail, the α, *a*-dicyanoalkenes 2 bearing electron-withdrawing (F, Cl, Br, I, ethynyl) and electron-donating (Me, MeO) groups at the phenyl ring could be efficiently converted into the desired products 3a–3n in 62–96% yields (Scheme 2, line 1). The relative configuration of 3a (CCDC 2100705) was determined by X-ray crystallographic analysis, and the relative configurations of other products **three** were tentatively assigned by analogy. Besides, the α, *a*-dicyanoalkenes 2 containing challenging disubstituted groups.

Were also available, giving the corresponding products 3o–3r in consistent high yields (Scheme 2, line 1). Moreover, aromatic series (naphthyl, furyl, and thienyl) could also participate in this [4 + 2] annulation sequence to provide expected products 3s, 3t and 3u in 79, 55 and 65% yields, respectively (Scheme 2, line 2). Subsequently, several *ortho*-tosylaminophenyl-*p*-QMs 1 were investigated to further verify the generality of this method. The results indicated that electronic-withdrawing substituents on the phenyl ring of substrate 1 showed rare affection on efficiency, delivering target products 3v and 3w in high yields (Scheme 2, line 2). Changing the type of protecting group still resulted in desire compound 3x with excellent reaction performance (Scheme 2, line 2). However, the unavailability of dimethyl substituted substrate failed to provide the expected product 3y. To evaluate the general utility and robustness of this protocol, we also conducted the gram-scale reaction under the standard condition, and the target product 3a could be smoothly isolated in 93% yield.

**Scheme 3:**
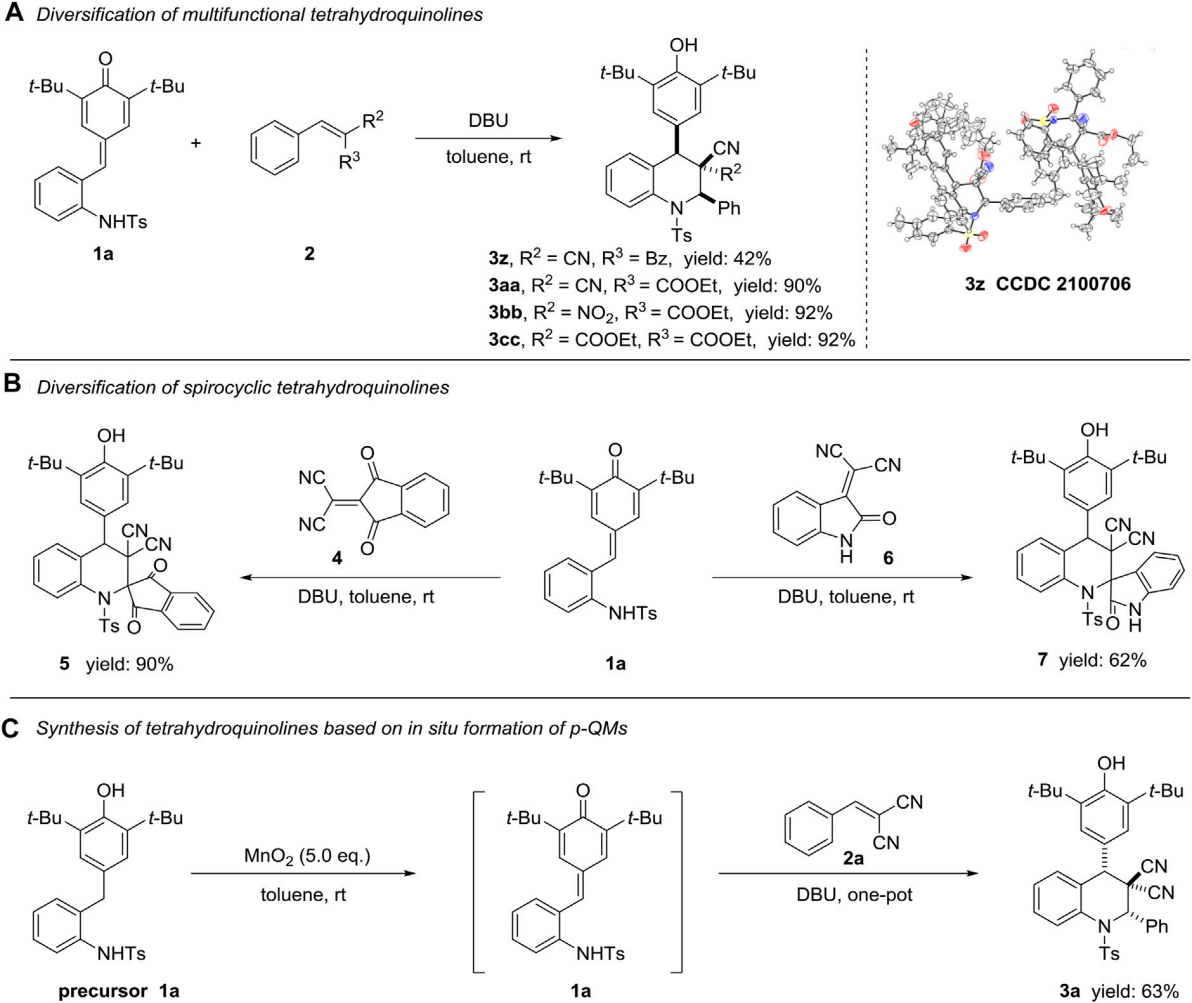
Diversification of multifunctional **(A)** and spirocyclic **(B)** tetrahydroquinoline derivatives, and one-pot synthesis of compound 3a **(C)**. All the reactions were conducted under the standard condition, dr > 20:1.

To enrich the diversification of this protocol towards functionalized tetrahydroquinoline derivatives, we established the verification with represented substrates 2 (Scheme 3). Fixed different electronic properties groups (such as nitro, benzoxyl and COOEt groups) on the position of *R* ^2^ and/or R^3^, all reaction could move forward the production of compounds 3z-3bb under optimal condition in 42, 90 and 92% yields, respectively (Scheme 3A). However, substrate containing two ester groups could not offer the target product 3cc under this condition. The relative configuration of 3aa (CCDC 2100706) was determined by X-ray crystallographic analysis, and the relative configurations of 3z–3bb were tentatively assigned by analogy. Moreover, we attempted to construct valuable spirocyclic frameworks employing tetra-substituted substrates **four** and **six** within the established condition, and successfully obtained desirable products **five** and **seven** in 90 and 62% yields, respectively (Scheme 3B). Regretfully, we failed to accurately assign the relative configuration of compound 5 and 7 with limited information (for details please see ESI). In order to further explore the robustness of this methodology, a preliminary attempt of one-pot synthesis of functionalized tetrahydroquinoline compound starting from precursor 1a was successfully conducted, producing target molecule 3a in 63% yield (Scheme 3C, Wang et al., 2018).

**Scheme 4:**
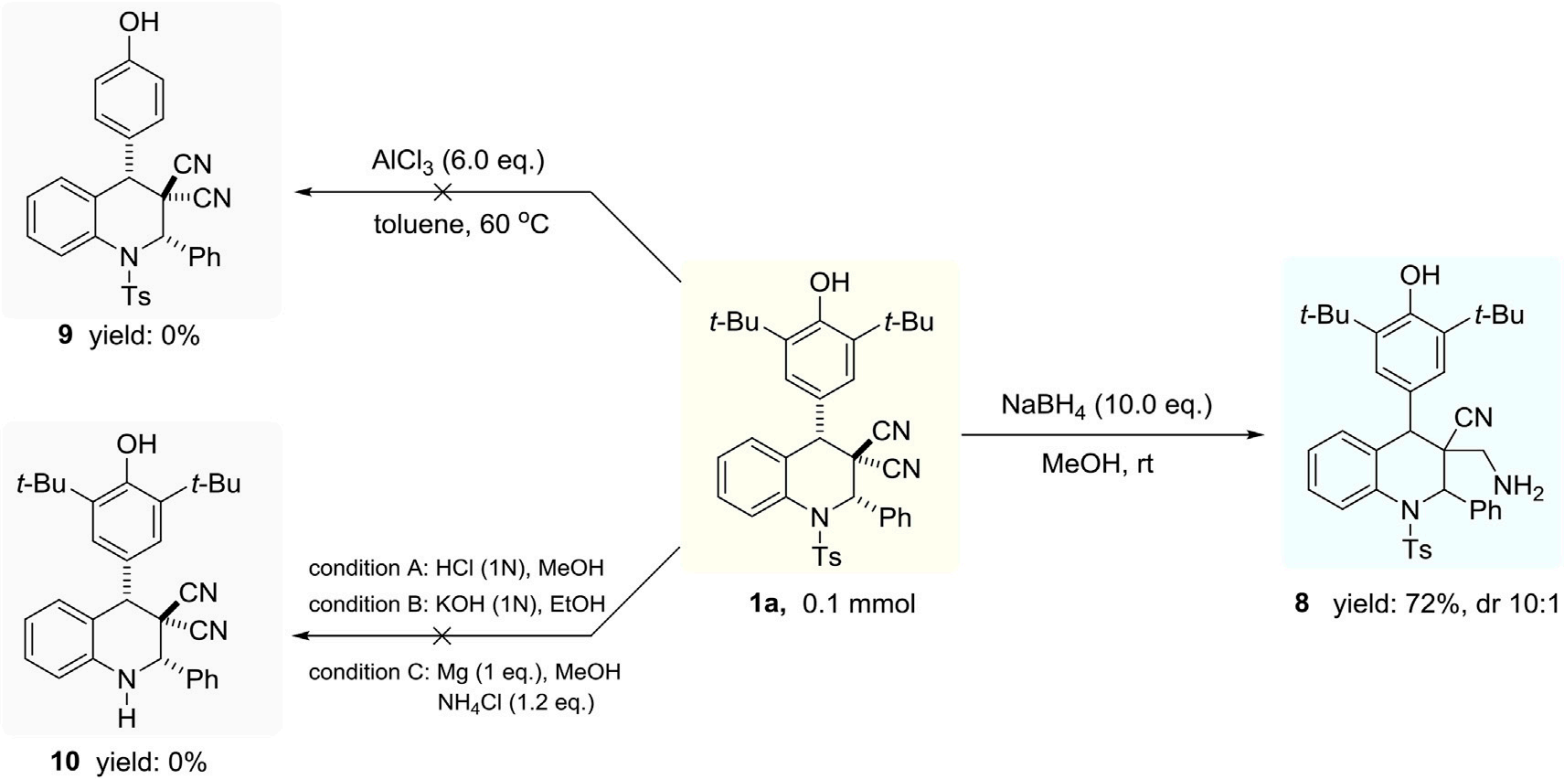
Transformation of multifunctional tetrahydroquinoline derivatives.

Meanwhile, the successful transformation of reducing cyan group into primary amine delivered the valuable product 8 with good reaction performance and undefined-relative configur–ation (for details please see ESI), which may show the potential application in medicinal chemistry (Scheme 4, right column). However, the removal of *para*-toluene sulfonamide and di-tertiary butyl groups failed to provide the expected products 9 and 10 (Scheme 4, left column).

## Conclusion

In conclusion, we have developed a DBU-mediated catalyst-free [4 + 2] annulation between *ortho*-tosylaminophenyl-substituted *p*-QMs and cyanoalkenes for synthesis of valuable tetrahydroquinoline derivatives through an aza-Michael/1,6-conjugate addition sequence. This protocol features broad of tolerance and diversification on substrates, offering straightforward route to various of 4-aryl-substituted tetrahydroquinolines with high yields, excellent diastereoselectivities as well as gram-scale capacity. Moreover, a one-pot reaction sequence utilizing *in situ* generated *p*-QMs under the similar condition to build tetrahydroquinoline framework is smoothly conducted with good reaction performance as well as step and atom economy. Further studies on the bioactivity of those promising tetrahydroquinolines will be reported in due course.

### Experiment

#### General Information

NMR data were obtained for ^1^H at 400 MHz and for ^13^C at 100 MHz, or for ^1^H at 600 MHz and for ^13^C at 150 MHz. Chemical shifts were reported in parts per million (ppm) using tetramethyl silane as internal standard with solvent resonance in CDCl_3_. UV detection was performed at 254 nm. ESI-HRMS spectra were measured with a Q-TOF instrument. Column chromatography was performed on a silica gel (200–300 mesh) using an eluent of ethyl acetate and petroleum ether. TLC was performed on glass-backed silica plates; products were visualized using UV light. Melting points were determined on a Mel-Temp apparatus. All reagents and solvents were obtained from commercial sources and used without further purification. Substrates 1 and precursor 1a were prepared according to the literature procedures (Wang et al., 2018; Wang J.-Y. et al., 2020). Substrates 2 (Ghosh et al., 2021) and other cyanoalkenes (Fioravanti et al., 2012; Donckele et al., 2015; Zhu et al., 2017; Chen et al., 2021) were prepared through the Knoevenagel reactions, and all substrates can be stored at 4°C for 25 days without decomposition.

General procedure for the synthesis of 3: The reaction was carried out with 1 (0.1 mmol), 2 (0.12 mmol) and DBU (0.02 mmol) in anhydrous toluene (2 ml) at room temperature for 1 h. Upon the consumption of substrate 1 (monitored by TLC), the reside was directly purified by flash column chromatography (PE/EA = 20:1 to 10:1) to provide the desired product 3. The one-pot synthesis of product 3a was conducted in similar manner and MnO_2_ (5.0 eq.) was added as oxidant in the reaction.

4-(3,5-di-tert-butyl-4-hydroxyphenyl)-2-phenyl-1-tosyl-1,4-dihydroquinoline-3,3(2H)-dicarbonitrile (3a). Compound 3a was synthesized in a manner of the general procedure for the synthesis of 3. Yield 96%, white powder solid, >20:1 dr, m. p. 215–216°C. ^1^H NMR (600 MHz, CDCl_3_) *δ* ppm 7.94 (dd, *J* = 7.8, 1.2 Hz, 1H), 7.92–7.87 (m, 1H), 7.63–7.57 (m, 2H), 7.57–7.52 (m, 2H), 7.49–7.37 (m, 5H), 7.32–7.28 (m, 2H), 6.84 (d, *J* = 7.8 Hz, 1H), 6.34 (s, 1H), 5.79 (s, 1H), 5.34 (s, 1H), 3.17 (s, 1H), 2.45 (s, 3H), 1.39 (s, 18H). ^13^C NMR (150 MHz, CDCl_3_) *δ* ppm 158.9, 153.5, 143.8, 135.6, 134.4, 133.61, 133.59, 132.4, 129.7, 128.9, 128.7, 128.6, 128.5, 128.0, 127.4, 126.9, 126.5, 126.4, 126.2, 121.2, 112.7, 111.4, 66.3, 49.9, 49.8, 30.6, 29.1, 20.7. HRMS (ESI): m/z [M + Na]^+^ calcd for C_38_H_39_N_3_NaO_3_S^+^: 640.2610; found 640.2614.

2-(2-chlorophenyl)-4-(3,5-di-tert-butyl-4-hydroxyphenyl)-1-tosyl-1,4-dihydroquinoline-3,3(2H)-dicarbonitrile (3b). Compound 3b was synthesized in a manner of the general procedure for the synthesis of 3. Yield 91%, white powder solid, >20:1 dr, m. p. 254–255 °C. ^1^H NMR (400 MHz, CDCl_3_) *δ* ppm 7.88 (d, *J =* 8.0 Hz, 1H), 7.59–7.54 (m, 2H), 7.53–7.46 (m, 3H), 7.42 (s, 1H), 7.35–7.24 (m, 5H), 6.89 (d, *J =* 7.6 Hz, 1H), 6.36 (s, 2H), 5.34 (s, 1H), 3.10 (s, 1H), 2.46 (s, 3H), 1.40 (s, 18H). ^13^C NMR (100 MHz, CDCl_3_) *δ* ppm 154.6, 145.1, 136.7, 135.6, 134.7, 134.2, 133.05, 133.02, 130.6, 130.3, 130.1, 129.9, 129.8, 128.5, 127.9, 127.6, 127.4, 122.1, 112.8, 112.2, 61.7, 51.6, 49.3, 34.3, 30.2, 21.8. HRMS (ESI): m/z [M + Na]^+^ calcd for C_38_H_38_ClN_3_NaO_3_S^+^: 674.2220; found 674.2215.

2-(2-bromophenyl)-4-(3,5-di-tert-butyl-4-hydroxyphenyl)-1-tosyl-1,4-dihydroquinoline-3,3(2H)-dicarbonitrile (3c). Compound 3c was synthesized in a manner of the general procedure for the synthesis of 3. Yield 71%, white powder solid, >20:1 dr, m. p. 241–242 °C. ^1^H NMR (400 MHz, CDCl_3_) *δ* ppm 7.87 (d, *J* = 8.4 Hz, 1H), 7.66 (dd, *J* = 8.0, 1.2 Hz, 1H), 7.57–7.53 (m, 2H), 7.51–7.49 (m, 2H), 7.42–7.37 (m, 2H), 7.34–7.30 (m, 3H), 7.26 (td, *J* = 8.0, 2.0 Hz, 1H), 6.89 (d, *J* = 7.6 Hz, 1H), 6.34 (s, 1H), 6.33 (s, 2H), 5.34 (s, 1H), 3.08 (s, 1H), 2.47 (s, 3H), 1.40 (s, 18H). ^13^C NMR (100 MHz, CDCl_3_) *δ* ppm 154.6, 145.0, 136.7, 136.2, 135.6, 134.2, 133.3, 133.1, 130.9, 130.7, 130.1, 129.8, 128.5, 128.0, 127.9, 127.60, 127.5, 123.4, 122.1, 112.8, 112.1, 63.8, 51.7, 49.3, 34.3, 30.2, 21.8. HRMS (ESI): m/z [M + Na]^+^ calcd for C_38_H_38_BrN_3_NaO_3_S^+^: 718.1715; found 718.1721.

4-(3,5-di-tert-butyl-4-hydroxyphenyl)-2-(2-iodophenyl)-1-tosyl-1,4-dihydroquinoline-3,3(2H)-dicarbonitrile (3d). Compound 3d was synthesized in a manner of the general procedure for the synthesis of 3. Yield 77%, white powder solid, >20:1 dr, m. p. 247–248°C. ^1^H NMR (400 MHz, CDCl_3_) *δ* ppm 7.95 (dd, *J* = 8.0, 1.2 Hz, 1H), 7.87 (dd, *J* = 8.1, 1.2 Hz, 1H), 7.67–7.48 (m, 4H), 7.46–7.38 (m, 2H), 7.34 (d, *J* = 8.0 Hz, 3H), 7.09 (td, *J* = 7.6, 1.6 Hz, 1H), 6.88 (d, *J* = 8.0 Hz, 1H), 6.33 (s, 1H), 6.15 (s, 1H), 5.33 (s, 1H), 3.06 (s, 1H), 2.47 (s, 3H), 1.40 (s, 18H). ^13^C NMR (100 MHz, CDCl_3_) *δ* ppm 154.6, 145.0, 140.3, 139.2, 136.7, 135.7, 134.2, 133.2, 131.0, 130.4, 130.0, 129.8, 128.8, 128.5, 127.9, 127.7, 127.5, 122.1, 112.8, 112.1, 99.4, 67.5, 51.6, 49.4, 34.3, 30.2, 21.8. HRMS (ESI): m/z [M + Na]^+^ calcd for C_38_H_38_IN_3_NaO_3_S^+^: 766.1576; found 766.1585.

4-(3,5-di-tert-butyl-4-hydroxyphenyl)-2-(3-fluorophenyl)-1-tosyl-1,4-dihydroquinoline-3,3(2H)-dicarbonitrile (3e). Compound 3e was synthesized in a manner of the general procedure for the synthesis of 3. Yield 89%, white powder solid, >20:1 dr, m. p. 215–216 °C. ^1^H NMR (400 MHz, CDCl_3_) *δ* ppm 7.96 (d, *J* = 8.0 Hz, 1H), 7.55 (td, *J* = 7.6, 1.2 Hz, 1H), 7.47–7.38 (m, 5H), 7.34–7.30 (m, 4H), 7.18–7.05 (m, 1H), 6.86 (d, *J* = 8.0 Hz, 1H), 6.34 (s, 1H), 5.79 (s, 1H), 5.35 (s, 1H), 3.13 (s, 1H), 2.45 (s, 3H), 1.40 (s, 18H). ^13^C NMR (100 MHz, CDCl_3_) *δ* ppm 164.1 (d, *J*_CF_ = 246.7 Hz), 154.6, 145.1, 139.2 (d, *J*_CF_ = 6.8 Hz), 136.8, 135.1, 134.4, 133.2, 130.8 (d, *J*_CF_ = 8.1 Hz), 130.0, 129.9, 128.4, 128.1, 127.6, 127.4, 123.0, 122.9 (d, *J*_CF_ = 3.1 Hz), 116.8 (d, *J*_CF_ = 21.1 Hz), 114.6 (d, *J*_CF_ = 23.1 Hz), 113.6, 112.2, 66.7, 50.9, 50.5, 34.3, 30.2, 21.8. HRMS (ESI): m/z [M + Na]^+^ calcd for C_38_H_38_FN_3_NaO_3_S^+^: 658.2516; found 658.2512.

2-(3-bromophenyl)-4-(3,5-di-tert-butyl-4-hydroxyphenyl)-1-tosyl-1,4-dihydroquinoline-3,3(2H)-dicarbonitrile (3f). Compound 3f was synthesized in a manner of the general procedure for the synthesis of 3. Yield 93%, white powder solid, >20:1 dr, m. p. 223–224°C. ^1^H NMR (400 MHz, CDCl_3_) *δ* ppm 7.94 (dd, *J* = 8.0, 1.2 Hz, 1H), 7.70 (d, *J* = 2.0 Hz, 1H), 7.64–7.47 (m, 3H), 7.42 (dd, *J* = 6.4, 1.6 Hz, 3H), 7.37–7.28 (m, 4H), 6.85 (d, *J* = 7.6 Hz, 1H), 6.34 (s, 1H), 5.72 (s, 1H), 5.35 (s, 1H), 3.13 (s, 1H), 2.45 (s, 3H), 1.40 (s, 18H). ^13^C NMR (100 MHz, CDCl_3_) *δ* ppm 154.6, 145.1, 138.8, 136.8, 135.1, 134.3, 133.1, 132.8, 130.6, 130.3, 130.0, 129.9, 128.4, 128.1, 127.6, 127.4, 126.0, 123.1, 122.1, 113.5, 112.2, 66.6, 50.9, 50.6, 34.7, 30.2, 21.8. HRMS (ESI): m/z [M + Na]^+^ calcd for C_38_H_38_BrN_3_NaO_3_S^+^: 718.1715; found 718.1720.

2-(4-chlorophenyl)-4-(3,5-di-tert-butyl-4-hydroxyphenyl)-1-tosyl-1,4-dihydroquinoline-3,3(2H)-dicarbonitrile (3g). Compound 3g was synthesized in a manner of the general procedure for the synthesis of 3. Yield 95%, white powder solid, >20:1 dr, m. p. 217–218°C. ^1^H NMR (400 MHz, CDCl_3_) *δ* ppm 7.93 (d, *J* = 8.0 Hz, 1H), 7.63–7.51 (m, 3H), 7.42 (dd, *J* = 8.4, 3.6 Hz, 5H), 7.30 (t, *J* = 7.6 Hz, 3H), 6.84 (d, *J* = 7.6 Hz, 1H), 6.34 (s, 1H), 5.74 (s, 1H), 5.35 (s, 1H), 3.13 (s, 1H), 2.45 (s, 3H), 1.40 (s, 18H). ^13^C NMR (100 MHz, CDCl_3_) *δ* ppm 171.2, 154.6, 145.1, 136.8, 135.6, 135.3, 135.2, 134.3, 133.2, 130.0, 129.9, 129.3, 128.7, 128.3, 128.1, 127.6, 127.4, 123.9, 122.1, 113.5, 112.3, 66.8, 60.4, 50.6, 34.5, 30.2, 21.7. HRMS (ESI): m/z [M + Na]^+^ calcd for C_38_H_38_ClN_3_NaO_3_S^+^: 674.2220; found 674.2223.

2-(4-bromophenyl)-4-(3,5-di-tert-butyl-4-hydroxyphenyl)-1-tosyl-1,4-dihydroquinoline-3,3(2H)-dicarbonitrile (3h). Compound 3h was synthesized in a manner of the general procedure for the synthesis of 3. Yield 95%, white powder solid, >20:1 dr, m. p. 235–236°C. ^1^H NMR (400 MHz, CDCl_3_) *δ* ppm 7.93 (d, *J* = 8.0 Hz, 1H), 7.59 (d, *J* = 8.0 Hz, 2H), 7.54 (t, *J* = 7.6 Hz, 1H), 7.48 (d, *J* = 8.4 Hz, 2H), 7.42 (d, *J* = 8.0 Hz, 3H), 7.30 (t, *J* = 7.6 Hz, 3H), 6.84 (d, *J* = 7.6 Hz, 1H), 6.34 (s, 1H), 5.72 (s, 1H), 5.35 (s, 1H), 3.13 (s, 1H), 2.45 (s, 3H), 1.40 (s, 18H). ^13^C NMR (100 MHz, CDCl_3_) *δ* ppm 154.6, 145.1, 136.8, 135.8, 135.1, 134.3, 133.2, 132.3, 130.0, 129.9, 129.0, 128.3, 128.1, 127.6, 127.4, 123.9, 122.1, 113.5, 112.3, 66.9, 50.9, 50.5, 34.5, 30.2, 21.8. HRMS (ESI): m/z [M + Na]^+^ calcd for C_38_H_38_BrN_3_NaO_3_S^+^: 718.1715; found 718.1709.

4-(3,5-di-tert-butyl-4-hydroxyphenyl)-2-(4-ethynylphenyl)-1-tosyl-1,4-dihydroquinoline-3,3(2H)-dicarbonitrile (3i). Compound 3i was synthesized in a manner of the general procedure for the synthesis of 3. Yield 92%, white powder solid, >20:1 dr, m. p. 248–249°C. ^1^H NMR (400 MHz, CDCl_3_) *δ* ppm 7.94 (d, *J* = 7.2 Hz, 1H), 7.62–7.50 (m, 5H), 7.43–7.41 (m, 3H), 7.35–7.28 (m, 3H), 6.85 (d, *J* = 7.6 Hz, 1H), 6.35 (s, 1H), 5.77 (s, 1H), 5.35 (s, 1H), 3.15 (s, 1H), 3.12 (s, 1H), 2.45 (s, 3H), 1.40 (s, 18H). ^13^C NMR (100 MHz, CDCl_3_) *δ* ppm 154.6, 145.1, 137.2, 136.8, 135.2, 134.4, 133.3, 132.8, 130.0, 129.8, 128.4, 128.7, 127.6, 127.4, 127.3, 123.5, 122.1, 113.5, 112.3, 82.9, 78.4, 67.0, 51.0, 50.5, 34.5, 30.2, 21.8. HRMS (ESI): m/z [M + Na]^+^ calcd for C_40_H_39_N_3_NaO_3_S^+^: 664.2610; found 664.2614.

4-(3,5-di-tert-butyl-4-hydroxyphenyl)-2-(o-tolyl)-1-tosyl-1,4-dihydroquinoline-3,3(2H)-dicarbonitrile (3j). Compound 3j was synthesized in a manner of the general procedure for the synthesis of 3. Yield 96%, white powder solid, >20:1 dr, m. p. 233–234°C. ^1^H NMR (400 MHz, CDCl_3_) *δ* ppm 7.81 (dd, *J* = 8.0, 1.2 Hz, 1H), 7.52 (td, *J* = 8.0, 1.6 Hz, 1H), 7.47–7.36 (m, 3H), 7.36–7.20 (m, 7H), 6.92 (dd, *J* = 7.6, 1.2 Hz, 1H), 6.41 (s, 1H), 6.13 (s, 1H), 5.34 (s, 1H), 3.25 (s, 1H), 2.75 (s, 3H), 2.45 (s, 3H), 1.41 (s, 18H). ^13^C NMR (100 MHz, CDCl_3_) *δ* ppm 154.6, 144.8, 136.6, 136.0, 135.6, 135.2, 134.9, 133.3, 130.9, 129.9, 129.7, 129.3, 129.0, 128.1, 127.8, 127.5, 127.3, 126.5, 122.2, 113.3, 112.9, 77.3, 77.0, 76.7, 62.1, 51.6, 50.0, 34.7, 30.2, 21.8, 21.7, 19.5. HRMS (ESI): m/z [M + Na]^+^ calcd for C_39_H_41_N_3_NaO_3_S^+^: 654.2766; found 654.2762.

4-(3,5-di-tert-butyl-4-hydroxyphenyl)-2-(2-methoxyphenyl)-1-tosyl-1,4-dihydroquinoline-3,3(2H)-dicarbonitrile (3k). Compound 3k was synthesized in a manner of the general procedure for the synthesis of 3. Yield 94%, white powder solid, >20:1 dr, m. p. 198–199°C. ^1^H NMR (400 MHz, CDCl_3_) *δ* ppm 7.97 (d, *J* = 8.0 Hz, 1H), 7.67–7.41 (m, 5H), 7.36–7.27 (m, 4H), 6.99 (t, *J* = 8.0 Hz, 2H), 6.94 (d, *J* = 8.4 Hz, 1H), 6.46 (s, 1H), 6.36 (s, 1H), 5.32 (s, 1H), 3.93 (s, 3H), 3.17 (s, 1H), 2.45 (s, 3H), 1.40 (s, 18H). ^13^C NMR (100 MHz, CDCl_3_) *δ* ppm 155.9, 154.5, 144.7, 136.3, 136.0, 135.5, 132.8, 130.4, 130.0, 129.5, 128.9, 128.0, 127.9, 127.6, 127.4, 126.2, 122.1, 121.0, 114.3, 113.2, 110.5, 60.6, 55.0, 51.1, 50.1, 34.6, 30.2, 21.8. HRMS (ESI): m/z [M + Na]^+^ calcd for C_39_H_41_N_3_NaO_4_S^+^: 670.2715; found 670.2718.

4-(3,5-di-tert-butyl-4-hydroxyphenyl)-2-(p-tolyl)-1-tosyl-1,4-dihydroquinoline-3,3(2H)-dicarbonitrile (3l)**.** Compound 3l was synthesized in a manner of the general procedure for the synthesis of 3. Yield 93%, white powder solid, >20:1 dr, m. p. 223–224°C. ^1^H NMR (400 MHz, CDCl_3_) *δ* ppm ^1^H NMR (400 MHz, CDCl_3_) δ 7.85 (d, *J* = 8.0 Hz, 1H), 7.45 (t, *J* = 7.6 Hz, 1H), 7.40–7.34 (m, 5H), 7.24–7.19 (m, 3H), 7.17 (d, *J* = 8.2 Hz, 2H), 6.75 (d, *J* = 7.6 Hz, 1H), 6.26 (s, 1H), 5.67 (s, 1H), 5.25 (s, 1H), 3.07 (s, 1H), 2.37 (s, 3H), 2.29 (s, 3H), 1.32 (s, 18H). ^13^C NMR (100 MHz, CDCl_3_) *δ* ppm 154.5, 144.8, 139.4, 136.7, 135.5, 134.7, 133.8, 133.4, 129.9, 129.7, 128.4, 127.9, 127.5, 127.4, 127.1, 124.0, 122.4, 113.7, 112.6, 67.3, 50.95, 50.92, 34.4, 30.2, 21.7, 21.3. HRMS (ESI): m/z [M + Na]^+^ calcd for C_39_H_41_N_3_NaO_3_S^+^: 654.2766; found 654.2773.

4-(3,5-di-tert-butyl-4-hydroxyphenyl)-2-(4-methoxyphenyl)-1-tosyl-1,4-dihydroquinoline-3,3(2H)-dicarbonitrile (3m). Compound 3m was synthesized in a manner of the general procedure for the synthesis of 3. Yield 88%, white powder solid, >20:1 dr, m. p. 210–211°C. ^1^H NMR (400 MHz, CDCl_3_) *δ* ppm 7.92 (dd, *J* = 8.0, 1.2 Hz, 1H), 7.54–7.50 (m, 3H), 7.43 (d, *J* = 8.0 Hz, 3H), 7.32–7.27 (m, 3H), 6.96 (d, *J* = 8.8 Hz, 2H), 6.83 (dt, *J* = 8.0, 1.6 Hz, 1H), 6.35 (s, 1H), 5.75 (s, 1H), 5.34 (s, 1H), 3.82 (s, 3H), 3.16 (s, 1H), 2.45 (s, 3H), 1.40 (s, 18H).^13^C NMR (100 MHz, CDCl_3_) *δ* ppm 160.4, 154.5, 144.8, 136.7, 135.5, 134.7, 133.5, 129.9, 129.7, 128.8, 128.6, 128.4, 127.9, 127.5, 127.4, 122.4, 114.4, 113.8, 112.6, 67.1, 55.3, 51.1, 50.9, 34.9, 30.2, 21.8. HRMS (ESI): m/z [M + Na]^+^ calcd for C_39_H_41_N_3_NaO_4_S^+^: 670.2715; found 670.2721.

4-(3,5-di-tert-butyl-4-hydroxyphenyl)-2-(3,4-dimethoxyphenyl)-1-tosyl-1,4-dihydroquinoline-3,3(2H)-dicarbonitrile (3n). Compound 3n was synthesized in a manner of the general procedure for the synthesis of 3. Yield 62%, white powder solid, >20:1 dr, m. p. 202–203°C. ^1^H NMR (400 MHz, CDCl_3_) *δ* ppm 7.92 (t, *J =* 1.6 Hz, 1H), 7.52 (td, *J =* 7.6, 1.6 Hz, 1H), 7.43 (d, *J =* 8.0 Hz, 3H), 7.30 (t, *J =* 7.2 Hz, 3H), 7.18 (dd, *J =* 8.0, 2.0 Hz, 1H), 7.08 (s, 1H), 6.92 (d, *J =* 8.4 Hz, 1H), 6.85 (d, *J =* 7.6 Hz, 1H), 6.36 (s, 1H), 5.76 (s, 1H), 5.34 (s, 1H), 3.89 (s, 3H), 3.87 (s, 3H), 3.17 (s, 1H), 2.45 (s, 3H), 1.40 (s, 18H). ^13^C NMR (100 MHz, CDCl_3_) δ 154.5, 149.9, 149.2, 144.9, 136.7, 135.5, 134.7, 133.4, 129.9, 129.7, 129.1, 128.1, 127.9, 127.6, 127.4, 122.3, 119.9, 113.9, 112.6, 111.3, 110.1, 67.2, 55.9, 55.8, 51.2, 50.9, 34.5, 30.2, 21.7. HRMS (ESI): m/z [M + Na]^+^ calcd for C_40_H_43_N_3_NaO_5_S^+^: 700.2821; found 700.2828.

4-(3,5-di-tert-butyl-4-hydroxyphenyl)-2-(2,4-dichlorophenyl)-1-tosyl-1,4-dihydroquinoline-3,3(2H)-dicarbonitrile (3o). Compound 3o was synthesized in a manner of the general procedure for the synthesis of 3. Yield 91%, white powder solid, >20:1 dr, m. p. 250–251°C. ^1^H NMR (400 MHz, Chloroform-*d*) *δ* ppm 7.91 (dd, *J* = 8.0, 1.2 Hz, 1H), 7.57 (td, *J* = 7.6, 1.6 Hz, 1H), 7.52 (d, *J* = 2.8 Hz, 1H), 7.46 (d, *J* = 8.4 Hz, 2H), 7.42 (d, *J* = 8.8 Hz, 2H), 7.39–7.30 (m, 4H), 6.90 (d, *J* = 7.6 Hz, 1H), 6.34 (s, 1H), 6.27 (s, 1H), 5.35 (s, 1H), 3.04 (s, 1H), 2.47 (s, 3H), 1.40 (s, 18H). ^13^C NMR (100 MHz, CDCl_3_) *δ* ppm 154.7, 145.2, 136.3, 135.3, 133.9, 133.4, 132.7, 131.3, 131.0, 130.8, 130.3, 130.1, 130.0, 128.5, 128.1, 127.6, 127.5, 121.9, 112.5, 111.9, 61.4, 51.6, 49.0, 34.7, 30.2, 21.8. HRMS (ESI): m/z [M + Na]^+^ calcd for C_38_H_37_Cl_2_N_3_NaO_3_S^+^: 708.1830; found 708.1841.

4-(3,5-di-tert-butyl-4-hydroxyphenyl)-2-(2,6-dichlorophenyl)-1-tosyl-1,4-dihydroquinoline-3,3(2H)-dicarbonitrile (3p). Compound 3p was synthesized in a manner of the general procedure for the synthesis of 3. Yield 89%, white powder solid, >20:1 dr, m. p. 303–304°C. ^1^H NMR (400 MHz, CDCl_3_) δ 7.85 (d, *J* = 8.0 Hz, 1H), 7.45 (t, *J* = 7.8 Hz, 1H), 7.43–7.24 (m, 5H), 7.22–7.20 (m, 3H), 7.17 (d, *J* = 8.4 Hz, 2H), 6.75 (d, *J* = 7.7 Hz, 1H), 6.26 (s, 1H), 5.67 (s, 1H), 5.25 (s, 1H), 3.07 (s, 1H), 2.37 (s, 3H), 2.29 (s, 3H), 1.32 (s, 18H).^13^C NMR (100 MHz, CDCl_3_) *δ* ppm 154.7, 145.1, 136.6, 136.55, 136.47, 136.0, 133.5, 131.8, 131.5, 131.0, 130.1, 130.0, 129.9, 129.4, 129.3, 128.3, 127.7, 127.4, 127.2, 124.3, 122.1, 113.0, 112.1, 62.5, 52.0, 47.1, 34.7, 34.2, 30.3, 30.1, 21.8. HRMS (ESI): m/z [M + Na]^+^ calcd for C_38_H_37_Cl_2_N_3_NaO_3_S^+^: 708.1830; found 708.1841.

2-(2-chloro-5-fluorophenyl)-4-(3,5-di-tert-butyl-4-hydroxyphenyl)-1-tosyl-1,4-dihydro-quinoline-3,3(2H)-dicarbonitrile (3q). Compound 3q was synthesized in a manner of the general procedure for the synthesis of 3. Yield 93%, white powder solid, >20:1 dr, m. p. 232–233°C. ^1^H NMR (400 MHz, CDCl_3_) *δ* ppm 7.91 (d, *J* = 8.0 Hz, 1H), 7.57 (t, *J* = 8.0 Hz, 1H), 7.51–7.42 (m, 3H), 7.41–7.32 (m, 4H), 7.29 (dt, *J* = 9.2, 2.4 Hz, 1H), 7.08 (td, *J* = 8.0, 3.2 Hz, 1H), 6.90 (d, *J* = 7.6 Hz, 1H), 6.34 (s, 1H), 6.29 (s, 1H), 5.36 (s, 1H), 3.06 (s, 1H), 2.47 (s, 3H), 1.40 (s, 18H). ^13^C NMR (100 MHz, CDCl_3_) *δ* ppm 162.5 (d, *J*_CF_ = 249.5 Hz), 154.7, 145.2, 136.6 (d, *J*_CF_ = 7.0 Hz), 135.3, 133.9, 132.8, 131.3 (d, *J*_CF_ = 7.7 Hz), 130.1, 130.0, 128.5, 128.1, 128.0 (d, *J*_CF_ = 3.5 Hz), 127.6, 127.5, 124.0, 121.9, 118.2 (d, *J*_CF_ = 23.3 Hz), 117.4 (d, *J*_CF_ = 24.4 Hz), 112.5, 112.0, 61.6, 51.6, 49.0, 34.6, 30.2, 21.8. HRMS (ESI): m/z [M + Na]^+^ calcd for C_38_H_37_ClFN_3_NaO_3_S^+^: 692.2126; found 692.2131.

2-(5-bromo-2-fluorophenyl)-4-(3,5-di-tert-butyl-4-hydroxyphenyl)-1-tosyl-1,4-dihydro-quinoline-3,3(2H)-dicarbonitrile (3r). Compound 3r was synthesized in a manner of the general procedure for the synthesis of 3. Yield 91%, white powder solid, >20:1 dr, m. p. 206–207°C. ^1^H NMR (400 MHz, CDCl_3_) *δ* ppm 7.92 (d, *J* = 8.0 Hz, 1H), 7.65 (dd, *J* = 6.0, 2.4 Hz, 1H), 7.57 (t, *J* = 8.0 Hz, 1H), 7.42 (d, *J* = 8.0 Hz, 3H), 7.32 (d, *J* = 8.0 Hz, 3H), 7.07 (t, *J* = 8.8 Hz, 1H), 6.89 (d, *J* = 7.6 Hz, 1H), 6.34 (s, 1H), 6.05 (s, 1H), 5.35 (s, 1H), 3.06 (s, 1H), 2.46 (s, 3H), 1.40 (s, 18H). ^13^C NMR (150 MHz, CDCl_3_) *δ* ppm 154.8, 145.4, 136.8 (d, *J*_CF_ = 12.9 Hz), 135.2, 134.4 (d, *J*_CF_ = 7.5 Hz), 134.1, 132.8, 132.4, 130.2, 130.1, 128.6, 128.2, 128.1, 127.7, 127.6, 124.1, 122.0, 117.8, 117.4, 112.5, 59.3, 51.3, 49.6, 34.7, 34.3, 30.3, 30.2, 21.9. HRMS (ESI): m/z [M + Na]^+^ calcd for C_38_H_37_BrFN_3_NaO_3_S^+^: 736.1621; found 736.1615.

4-(3,5-di-tert-butyl-4-hydroxyphenyl)-2-(naphthalen-1-yl)-1-tosyl-1,4-dihydroquinoline-3,3(2H)-dicarbonitrile (3s). Compound 3s was synthesized in a manner of the general procedure for the synthesis of 3. Yield 79%, white powder solid, >20:1 dr, m. p. 255–256°C. ^1^H NMR (400 MHz, CDCl_3_) *δ* ppm 8.43 (d, *J* = 8.4 Hz, 1H), 8.00–7.83 (m, 3H), 7.74 (d, *J* = 7.2 Hz, 1H), 7.67 (td, *J* = 8.8, 1.6 Hz, 1H), 7.60–7.51 (m, 3H), 7.48 (t, *J* = 8.0 Hz 3H), 7.35 (td, *J* = 7.6, 1.2 Hz, 1H), 7.28 (d, *J* = 8.0 Hz, 2H), 6.98 (d, *J* = 7.8 Hz, 1H), 6.81 (s, 1H), 6.50 (s, 1H), 5.34 (s, 1H), 3.45 (s, 1H), 2.44 (s, 3H), 1.40 (s, 18H). ^13^C NMR (100 MHz, CDCl_3_) *δ* ppm 153.6, 143.8, 135.6, 135.1, 133.8, 132.7, 132.2, 132.1, 129.0, 128.9, 128.7, 128.1, 127.2, 126.8, 126.6, 126.4, 126.4, 126.0, 125.1, 124.2, 121.2, 121.1, 112.3, 111.7, 60.0, 50.8, 49.5, 33.4, 29.2, 20.7. HRMS (ESI): m/z [M + Na]^+^ calcd for C_42_H_41_N_3_NaO_3_S^+^: 690.2766; found 690.2769.

4-(3,5-di-tert-butyl-4-hydroxyphenyl)-2-(furan-2-yl)-1-tosyl-1,4-dihydroquinoline-3,3(2H)-dicarbonitrile (3t). Compound 3t was synthesized in a manner of the general procedure for the synthesis of 3. Yield 55%, white powder solid, >20:1 dr, m. p. 179–180°C. ^1^H NMR (400 MHz, CDCl_3_) *δ* ppm 7.67 (dd, *J* = 8.1, 1.3 Hz, 1H), 7.39–7.36 (m, 2H), 7.31 (td, *J* = 7.8, 1.6 Hz, 1H), 7.22 (d, *J* = 8.1 Hz, 2H), 7.15 (td, *J* = 7.6, 1.2 Hz, 1H), 6.60 (s, 2H), 6.50 (d, *J* = 7.8 Hz, 1H), 5.22 (s, 2H), 5.16 (s, 1H), 4.12 (q, *J* = 7.2 Hz, 1H), 2.97 (s, 1H), 2.46 (s, 3H), 2.04 (s, 1H), 1.37 (s, 18H). ^13^C NMR (100 MHz, CDCl_3_) *δ* ppm 153.2, 152.1, 145.5, 137.6, 135.9, 134.4, 133.5, 129.84, 129.77, 127.9, 127.4, 127.3, 126.9, 126.7, 125.7, 118.4, 60.4, 50.1, 41.8, 34.3, 30.2, 21.7. HRMS (ESI): m/z [M + Na]^+^ calcd for C_36_H_37_N_3_NaO_4_S^+^: 630.2402; found 630.2409.

4-(3,5-di-tert-butyl-4-hydroxyphenyl)-2-(thiophen-2-yl)-1-tosyl-1,4-dihydroquinoline-3,3(2H)-dicarbonitrile (3u). Compound 3u was synthesized in a manner of the general procedure for the synthesis of 3. Yield 65%, white powder solid, >20:1 dr, m. p. 238–239°C. ^1^H NMR (400 MHz, CDCl_3_) *δ* ppm 7.89 (d, *J* = 8.0 Hz, 1H), 7.51 (td, *J* = 7.6, 1.6 Hz, 1H), 7.44–7.42 (m, 3H), 7.40–7.34 (m, 2H), 7.33–7.28 (m, 3H), 7.07 (dd, *J* = 5.2, 3.6 Hz, 1H), 6.84 (d, *J* = 7.6 Hz, 1H), 6.32 (s, 1H), 6.13 (s, 1H), 5.34 (s, 1H), 3.12 (s, 1H), 2.45 (s, 3H), 1.40 (s, 18H). ^13^C NMR (100 MHz, CDCl_3_) *δ* ppm 154.6, 145.1, 140.4, 136.7, 134.6, 134.5, 133.3, 130.0, 129.7, 128.8, 128.2, 127.7, 127.6, 127.4, 126.5, 126.3, 122.1, 113.7, 112.1, 63.8, 51.3, 50.6, 34.6, 30.2, 21.6. HRMS (ESI): m/z [M + Na]^+^ calcd for C_36_H_37_N_3_NaO_3_S_2_^+^: 646.2174; found 646.2171.

7-bromo-4-(3,5-di-tert-butyl-4-hydroxyphenyl)-2-phenyl-1-tosyl-1,4-dihydroquinoline-3,3(2H)-dicarbonitrile (3v). Compound 3v was synthesized in a manner of the general procedure for the synthesis of 3. Yield 93%, white powder solid, >20:1 dr, m. p. 225–226°C. ^1^H NMR (400 MHz, CDCl_3_) *δ* ppm 7.74 (d, *J* = 8.5 Hz, 1H), 7.59 (dd, *J* = 8.2, 2.4 Hz, 1H), 7.51–7.45 (m, 2H), 7.41–7.29 (m, 6H), 7.26 (d, *J* = 8.0 Hz, 2H), 6.96 (s, 1H), 6.28 (s, 1H), 5.69 (s, 1H), 5.31 (s, 1H), 3.04 (s, 1H), 2.39 (s, 3H), 1.33 (s, 18H). ^13^C NMR (100 MHz, CDCl_3_) *δ* ppm 154.9, 145.2, 136.9, 136.3, 135.1, 134.6, 134.4, 132.9, 130.8, 130.1, 129.9, 129.7, 129.1, 127.4, 127.2, 121.8, 121.4, 113.4, 112.2, 67.2, 50.9, 50.7, 34.3, 30.2, 21.8. HRMS (ESI): m/z [M + Na]^+^ calcd for C_38_H_38_BrN_3_NaO_3_S^+^: 718.1715; found 718.1721.

6-chloro-4-(3,5-di-tert-butyl-4-hydroxyphenyl)-2-phenyl-1-tosyl-1,4-dihydroquinoline-3,3(2H)-dicarbonitrile (3w). Compound 3w was synthesized in a manner of the general procedure for the synthesis of 3. Yield 87%, white powder solid, >20:1 dr, m. p. 250–251°C. ^1^H NMR (400 MHz, CDCl_3_) *δ* ppm 7.88 (d, *J* = 8.8 Hz, 1H), 7.62–7.53 (m, 2H), 7.51 (dd, *J* = 8.4, 2.4 Hz, 1H), 7.50–7.36 (m, 6H), 7.34–7.32 (m, 2H), 6.87 (d, *J* = 2.4 Hz, 1H),6.34 (s, 1H), 5.77 (s, 1H), 5.38 (s, 1H), 3.12 (s, 1H), 2.47 (s, 3H), 1.41 (s, 18H). ^13^C NMR (100 MHz, CDCl_3_) *δ* ppm 154.8, 145.2, 136.9, 136.3, 135.0, 134.4, 134.1, 133.9, 130.1, 129.9, 129.7, 129.6, 129.1, 127.9, 127.4, 127.2, 121.5, 113.4, 112.2, 67.2, 60.4, 50.8, 34.4, 30.2, 21.8. HRMS (ESI): m/z [M + Na]^+^ calcd for C_38_H_38_ClN_3_NaO_3_S^+^: 674.2220; found 674.2223.

4-(3,5-di-tert-butyl-4-hydroxyphenyl)-2-phenyl-1-(phenylsulfonyl)-1,4-dihydroquinoline-3,3(2H)-dicarbonitrile (3y). Compound 3y was synthesized in a manner of the general procedure for the synthesis of 3. Yield 90%, white powder solid, >20:1 dr, m. p. 211–212°C. ^1^H NMR (400 MHz, CDCl_3_) *δ* ppm 7.95 (d, *J* = 8.0 Hz, 1H), 7.70 (tt, *J* = 6.8, 2.0 Hz, 1H), 7.63–7.58 (m, 2H), 7.57–7.49 (m, 5H), 7.48–7.37 (m, 4H), 7.32 (t, *J* = 7.6 Hz, 1H), 6.82 (d, *J* = 8.0 Hz, 1H), 6.23 (s, 1H), 5.79 (s, 1H), 5.33 (s, 1H), 2.96 (s, 1H), 1.39 (s, 18H). ^13^C NMR (100 MHz, CDCl_3_) *δ* ppm 154.5, 137.4, 136.7, 136.6, 135.3, 133.8, 133.6, 129.8, 129.6, 129.4, 129.1, 128.5, 128.1, 127.5, 127.3, 127.2, 123.9, 122.1, 113.7, 112.4, 67.4, 50.9, 50.7, 34.5, 30.2. HRMS (ESI): m/z [M + Na]^+^ calcd for C_37_H_37_N_3_NaO_3_S^+^: 626.2453; found 626.2457.

3-benzoyl-4-(3,5-di-tert-butyl-4-hydroxyphenyl)-2-phenyl-1-tosyl-1,2,3,4-tetrahydroquinoline-3-carbonitrile (3z). Compound 3z was synthesized in a manner of the general procedure for the synthesis of 3. Yield 42%, white powder solid, >20:1 dr, m. p. 269–270°C. ^1^H NMR (400 MHz, CDCl_3_) *δ* ppm 8.04 (dd, *J* = 8.0, 1.2 Hz, 1H), 7.60–7.54 (m, 2H), 7.50 (dd, *J* = 8.4, 7.6 Hz, 1H), 7.46–7.30 (m, 7H), 7.28–7.26 (m, 1H), 7.25–7.20 (m, 1H), 7.16 (d, *J* = 2.4 Hz, 1H), 7.04–7.00 (m, 2H), 6.81 (dd, *J* = 6.4, 1.6 Hz, 1H), 6.69–6.55 (m, 2H), 6.27 (s, 1H), 6.13 (d, *J* = 2.4 Hz, 1H), 5.11 (s, 1H), 3.18 (s, 1H), 2.45 (s, 3H), 1.41 (s, 9H), 1.01 (s, 9H). ^13^C NMR (150 MHz, CDCl_3_) *δ* ppm 196.0, 153.8, 144.3, 138.9, 137.5, 136.4, 136.0, 136.0, 135.2, 134.8, 132.1, 129.6, 129.0, 128.8, 128.7, 128.6, 128.4, 127.9, 127.6, 127.3, 127.1, 125.2, 122.7, 118.6, 67.9, 67.7, 53.3, 34.2, 34.2, 30.2, 30.0, 21.8. HRMS (ESI): m/z [M + Na]^+^ calcd for C_44_H_44_N_2_NaO_4_S^+^: 719.2919; found 719.2925.

Ethyl-3-cyano-4-(3,5-di-tert-butyl-4-hydroxyphenyl)-2-phenyl-1-tosyl-1,2,3,4-tetrahydro-quinoline-3-carboxylate (3aa). Compound 3aa was synthesized in a manner of the general procedure for the synthesis of 3. Yield 90%, white powder solid, >20:1 dr, m. p. 255–256°C. ^1^H NMR (400 MHz, CDCl_3_) *δ* ppm 7.99 (d, *J* = 8.0 Hz, 1H), 7.58–7.43 (m, 4H), 7.36–7.35 (m, 4H), 7.34–7.28 (m, 1H), 7.28–7.24 (m, 3H), 6.89 (d, *J* = 8.0 Hz, 1H), 6.13 (s, 1H), 6.10 (s, 1H), 5.23 (s, 1H), 3.91–3.71 (m, 2H), 3.02 (s, 1H), 2.42 (s, 3H), 1.36 (s, 18H), 0.73 (t, *J* = 7.6 Hz, 3H). ^13^C NMR (100 MHz, CDCl_3_) *δ* ppm 166.2, 154.0, 144.2, 138.6, 136.1, 135.9, 135.8, 134.8, 134.7, 129.5, 128.79, 128.77, 128.6, 128.5, 127.9, 127.7, 127.3, 127.0, 126.7, 125.2, 122.9, 116.0, 66.1, 64.7, 63.2, 50.9, 34.5, 34.1, 30.3, 30.1, 21.7, 13.3. HRMS (ESI): m/z [M + Na]^+^ calcd for C_40_H_44_N_2_NaO_5_S^+^: 687.2869; found 687.2873.

Ethyl-4-(3,5-di-tert-butyl-4-hydroxyphenyl)-3-nitro-2-phenyl-1-tosyl-1,2,3,4-tetrahydro-quinoline-3-carboxylate (3bb). Compound 3bb was synthesized in a manner of the general procedure for the synthesis of 3. Yield 92%, white powder solid, >20:1 dr, m. p. 166–167°C. ^1^H NMR (600 MHz, CDCl_3_) *δ* ppm 7.92 (d, *J* = 7.8 Hz, 1H), 7.56 (d, *J* = 8.4 Hz, 2H), 7.45 (t, *J* = 7.8 Hz, 1H), 7.31–7.20 (m, 8H), 7.15 (s, 1H), 6.95 (d, *J* = 8.4 Hz, 1H), 6.56 (s, 2H), 5.20 (s, 1H), 3.35 (dd, *J* = 10.8, 7.2 Hz, 1H), 3.28 (s, 1H), 3.18 (dd, *J* = 10.8, 7.2 Hz, 1H), 2.39 (s, 3H), 1.34 (s, 18H), 0.56 (t, *J* = 7.2 Hz, 3H). ^13^C NMR (150 MHz, CDCl_3_) *δ* ppm 163.7, 153.9, 144.3, 139.4, 136.1, 135.9, 135.6, 134.6, 129.8, 128.4, 128.4, 128.1, 127.7, 127.5, 127.4, 126.9, 123.2, 106.0, 65.4, 62.2, 52.8, 34.3, 30.3, 21.9, 12.9. HRMS (ESI): m/z [M + Na]^+^ calcd for C_39_H_44_N_2_NaO_7_S^+^: 707.2767; found 707.2772.

4'-(3,5-di-tert-butyl-4-hydroxyphenyl)-1,3-dioxo-1′-tosyl-1,1′,3,4′-tetrahydro-3′H-spiro[indene-2,2′-quinoline]-3′,3′-dicarbonitrile (5). Compound 5 was synthesized in a similar manner of the general procedure for the synthesis of 3. Yield 90%, white powder solid, >20:1 dr, m. p. 239–240°C. ^1^H NMR (400 MHz, CDCl_3_) *δ* ppm 8.17–7.13 (m, 2H), 8.06–7.91 (m, 4H), 7.68 (d, *J* = 8.0 Hz, 1H), 7.35–7.32 (m, 3H), 7.20 (td, *J* = 8.4, 1.6 Hz, 1H), 7.10 (s, 1H), 7.01 (t, *J* = 7.6 Hz, 1H), 6.94 (d, *J* = 7.6 Hz, 1H), 5.38 (s, 1H), 5.14 (s, 1H), 2.40 (s, 3H), 1.45 (s, 9H), 1.38 (s, 9H). ^13^C NMR (100 MHz, CDCl_3_) *δ* ppm 195.4, 191.1, 154.9, 145.4, 139.0, 138.9, 137.4, 137.2, 136.9, 136.4, 135.8, 130.1, 129.3, 129.0, 128.4, 124.9, 124.7, 124.3, 123.9, 123.3, 119.5, 110.5, 110.3, 68.7, 47.4, 45.6, 34.7, 34.4, 30.3, 21.7. HRMS (ESI): m/z [M + Na]^+^ calcd for C_40_H_37_N_3_NaO_5_S^+^: 694.2352; found 694.2359.

4'-(3,5-di-tert-butyl-4-hydroxyphenyl)-2-oxo-1′-tosyl-1′,4′-dihydro-3′H-spiro[indoline-3,2′-quinoline]-3′,3′-dicarbonitrile (7). Compound 7 was synthesized in a similar manner of the general procedure for the synthesis of 3. Yield 62%, red powder solid, >20:1 dr, m. p. 201–202°C. ^1^H NMR (600 MHz, CDCl_3_) *δ* ppm 8.11 (d, *J* = 7.8 Hz, 1H), 7.63 (d, *J* = 8.4 Hz, 2H), 7.47 (dd, *J* = 7.8, 1.8 Hz, 1H), 7.36–7.30 (m, 1H), 7.30–7.26 (m, 1H), 7.19 (d, *J* = 8.4 Hz, 2H), 7.16 (dd, *J* = 7.8, 1.8 Hz, 1H), 7.13 (dd, *J* = 7.8, 1.2 Hz, 1H), 7.06 (td, *J* = 7.8, 1.2 Hz, 1H), 6.82 (m, 2H), 6.76 (s, 1H), 6.52 (s, 1H), 6.37 (d, *J* = 8.4 Hz, 1H), 5.32 (s, 1H), 2.36 (s, 3H), 1.33 (s, 18H). ^13^C NMR (150 MHz, CDCl_3_) *δ* ppm 163.2, 154.1, 148.7, 146.0, 144.2, 137.1, 136.9, 136.7, 135.0, 131.4, 129.9, 129.6, 128.9, 127.1, 126.8, 126.6, 126.5, 125.0, 124.5, 123.8, 118.6, 113.0, 112.4, 110.7, 82.9, 55.7, 34.5, 30.2, 21.7. HRMS (ESI): m/z [M + Na]^+^ calcd for C_39_H_38_N_7_NaO_4_S^+^: 681.2511; found 681.2514.

Procedure for the synthesis of 8: The reaction was carried out with 1a (0.1 mmol), NaBH_4_ (10.0 eq.) in anhydrous MeOH (2 ml) at room temperature for several hours. Upon the consumption of substrate **1** (monitored by TLC), the reaction was quenched with aqueous NaHCO_3_, and extraction was carried out with CH_2_Cl_2_. The organic layer was dried over Na_2_SO_4_ and concentrated. Then, the reside was directly purified by flash column chromatography (PE/EA = 10:1 to 8:1) to provide the desired product **8**.

3-(aminomethyl)-4-(3,5-di-tert-butyl-4-hydroxyphenyl)-2-phenyl-1-tosyl-1,2,3,4-tetrahydro-quinoline-3-carbonitrile (8). Yield 72%, white powder solid, 10:1 dr, m. p. 235–236°C. ^1^H NMR (600 MHz, CDCl_3_) *δ* ppm 7.82 (dd, *J* = 8.4, 1.0 Hz, 1H), 7.38 (d, *J* = 8.4 Hz, 3H), 7.35 (d, *J* = 7.2 Hz, 3H), 7.28 (t, *J* = 7.8 Hz, 2H), 7.26–7.19 (m, 2H), 7.13 (d, *J* = 8.4 Hz, 3H), 6.65 (d, *J* = 7.8 Hz, 1H), 5.80 (s, 1H), 5.19 (s, 1H), 2.82 (s, 1H), 2.58 (dd, *J* = 28.8 Hz, 13.8 Hz, 2H), 2.33 (s, 3H), 1.31 (s, 18H), 0.78 (s, 2H).^13^C NMR (150 MHz, CDCl_3_) *δ* ppm 153.8, 143.9, 139.5, 137.1, 136.7, 136.1, 135.7, 129.3, 128.4, 128.4, 128.3, 128.2, 127.9, 127.8, 127.2, 126.8, 124.4, 120.2, 62.9, 56.6, 47.0, 43.6, 34.5, 30.4, 21.8. HRMS (ESI): m/z [M + H]^+^ calcd for C_38_H_44_N_3_O_3_S^+^: 622.3103; found 622.3107.

Procedure for the synthesis of 9: The reaction was carried out with 1a (0.1 mmol), AlCl_3_ (6.0 eq.) in anhydrous Toluene (2 ml) at room temperature. Upon the end of the reaction, the reaction mixture was quenched with aqueous NaHCO_3_, and extraction was carried out with CH_2_Cl_2_. The organic layer was dried over Na_2_SO_4_ and concentrated. Then, the reside was directly purified by flash column chromatography. This condition led to a mess reaction and could not offer target product **9**.

Procedure for the synthesis of 10: condition A: the reaction was carried out with 1a (0.1 mmol), HCl (1N) in anhydrous MeOH (2 ml) at room temperature for several hours; condition B: the reaction was carried out with 1a (0.1 mmol), KOH (1N) in anhydrous EtOH (2 ml) at room temperature for several hours; condition C: the reaction was carried out with 1a (0.1 mmol), Mg (1 eq.) and NH_4_Cl (1.2 eq.) in anhydrous MeOH (2 ml) at room temperature for several hours. The condition A and C led to no reaction, and condition B led to a mess reaction.

## Data Availability

The datasets presented in this study can be found in online repositories. The names of the repository/repositories and accession number(s) can be found in the article/Supplementary Material.
