# Supplementary material for: Highly Diastereoselective Synthesis of Tetrahydroquinoline Derivatives via [4 + 2] Annulation of Ortho-Tosylaminophenyl-Substituted Para-Quinone Methides and Cyanoalkenes
Source: Front Chem. 2021 Nov 3;9:764866. doi: 10.3389/fchem.2021.764866 (PMC8595915; doi:10.3389/fchem.2021.764866)

checkCIF/PLATON report

Structure factors have been supplied for datablock(s) 1

THIS REPORT IS FOR GUIDANCE ONLY. IF USED AS PART OF A REVIEW PROCEDURE FOR PUBLICATION, IT SHOULD NOT REPLACE THE EXPERTISE OF AN EXPERIENCED CRYSTALLOGRAPHIC REFEREE.

No syntax errors found. CIF dictionary Interpreting this report

Datablock: 1

```
Bond precision:  C-C = 0.0047 A          Wavelength=0.71073

Cell:           a=21.6637(17)    b=16.6690(13)      c=21.9890(18)
                alpha=90         beta=112.221(1)    gamma=90
Temperature:    273 K

                Calculated                 Reported
Volume          7350.8(10)                 7350.8(10)
Space group     P 21/n                     P 1 21/n 1
Hall group      -P 2yn                     -P 2yn
Moiety formula  C40 H44 N2 O5 S            C40 H44 N2 O5 S
Sum formula     C40 H44 N2 O5 S            C40 H44 N2 O5 S
Mr              664.83                     664.83
Dx,g cm-3       1.202                      1.201
Z               8                          8
Mu (mm-1)       0.133                      0.133
F000            2832.0                     2832.0
F000’           2834.19
h,k,lmax        25,19,26                   25,19,26
Nref            12944                      12912
Tmin,Tmax                                  0.640,0.746
Tmin’

Correction method= # Reported T Limits: Tmin=0.640 Tmax=0.746
AbsCorr = MULTI-SCAN

Data completeness= 0.998          Theta(max)= 25.000

R(reflections)= 0.0510( 6065)     wR2(reflections)= 0.1262( 12912)

S = 0.896                  Npar= 923
```

The following ALERTS were generated. Each ALERT has the format
test-name_ALERT_alert-type_alert-level.
Click on the hyperlinks for more details of the test.

Alert level B

```
PLAT420_ALERT_2_B D-H Bond Without Acceptor  O6       --H6       .     Please Check
PLAT420_ALERT_2_B D-H Bond Without Acceptor  O7       --H7       .     Please Check
PLAT910_ALERT_3_B Missing # of FCF Reflection(s) Below Theta(Min).         11 Note
```

Alert level C

```
PLAT026_ALERT_3_C Ratio Observed / Unique Reflections (too) Low ..        47% Check
PLAT053_ALERT_1_C Minimum Crystal Dimension Missing (or Error) ...     Please Check
PLAT054_ALERT_1_C Medium  Crystal Dimension Missing (or Error) ...     Please Check
PLAT055_ALERT_1_C Maximum Crystal Dimension Missing (or Error) ...     Please Check
PLAT220_ALERT_2_C NonSolvent   Resd 1  C   Ueq(max)/Ueq(min) Range        3.3 Ratio
PLAT220_ALERT_2_C NonSolvent   Resd 2  C   Ueq(max)/Ueq(min) Range        3.5 Ratio
PLAT222_ALERT_3_C NonSolvent Resd 1  H   Uiso(max)/Uiso(min) Range        4.1 Ratio
PLAT222_ALERT_3_C NonSolvent Resd 2  H   Uiso(max)/Uiso(min) Range        4.3 Ratio
PLAT230_ALERT_2_C Hirshfeld Test Diff for    S2       --O5       .        6.0 s.u.
PLAT230_ALERT_2_C Hirshfeld Test Diff for    S2       --O8       .        7.0 s.u.
PLAT230_ALERT_2_C Hirshfeld Test Diff for    S1       --O4       .        6.5 s.u.
PLAT234_ALERT_4_C Large Hirshfeld Difference C21      --C19      .       0.17 Ang.
PLAT234_ALERT_4_C Large Hirshfeld Difference C37      --C79      .       0.16 Ang.
PLAT234_ALERT_4_C Large Hirshfeld Difference C37      --C79A     .       0.16 Ang.
PLAT242_ALERT_2_C Low    'MainMol' Ueq as Compared to Neighbors of        C21 Check
PLAT242_ALERT_2_C Low    'MainMol' Ueq as Compared to Neighbors of        C43 Check
PLAT242_ALERT_2_C Low    'MainMol' Ueq as Compared to Neighbors of         C3 Check
PLAT242_ALERT_2_C Low    'MainMol' Ueq as Compared to Neighbors of        C25 Check
PLAT242_ALERT_2_C Low    'MainMol' Ueq as Compared to Neighbors of        C55 Check
PLAT340_ALERT_3_C Low Bond Precision on  C-C Bonds ...............    0.00468 Ang.
PLAT906_ALERT_3_C Large K Value in the Analysis of Variance ......      3.870 Check
PLAT911_ALERT_3_C Missing FCF Refl Between Thmin & STh/L=    0.595         21 Report
```

Alert level G

```
PLAT003_ALERT_2_G Number of Uiso or Uij Restrained non-H Atoms ...         10 Report
PLAT007_ALERT_5_G Number of Unrefined Donor-H Atoms ..............          2 Report
PLAT186_ALERT_4_G The CIF-Embedded .res File Contains ISOR Records          3 Report
PLAT187_ALERT_4_G The CIF-Embedded .res File Contains RIGU Records          3 Report
PLAT199_ALERT_1_G Reported _cell_measurement_temperature ..... (K)        273 Check
PLAT200_ALERT_1_G Reported   _diffrn_ambient_temperature ..... (K)        273 Check
PLAT300_ALERT_4_G Atom Site Occupancy of C79        Constrained at        0.5 Check
PLAT300_ALERT_4_G Atom Site Occupancy of C79A       Constrained at        0.5 Check
PLAT300_ALERT_4_G Atom Site Occupancy of H37A       Constrained at        0.5 Check
PLAT300_ALERT_4_G Atom Site Occupancy of H37B       Constrained at        0.5 Check
PLAT300_ALERT_4_G Atom Site Occupancy of H37C       Constrained at        0.5 Check
PLAT300_ALERT_4_G Atom Site Occupancy of H37D       Constrained at        0.5 Check
PLAT300_ALERT_4_G Atom Site Occupancy of H79A       Constrained at        0.5 Check
PLAT300_ALERT_4_G Atom Site Occupancy of H79B       Constrained at        0.5 Check
PLAT300_ALERT_4_G Atom Site Occupancy of H79C       Constrained at        0.5 Check
PLAT300_ALERT_4_G Atom Site Occupancy of H79D       Constrained at        0.5 Check
PLAT300_ALERT_4_G Atom Site Occupancy of H79E       Constrained at        0.5 Check
PLAT300_ALERT_4_G Atom Site Occupancy of H79F       Constrained at        0.5 Check
PLAT301_ALERT_3_G Main Residue  Disorder ..............(Resd  1  )         8% Note
PLAT412_ALERT_2_G Short Intra XH3 .. XHn     H38      ..H39E     .       2.10 Ang.
                                                    x,y,z  =      1_555 Check
PLAT412_ALERT_2_G Short Intra XH3 .. XHn     H38      ..H39A     .       1.83 Ang.
                                                    x,y,z  =      1_555 Check
PLAT414_ALERT_2_G Short Intra D-H..H-X       H7       ..H19E             1.93 Ang.
                                                    x,y,z  =      1_555 Check
PLAT414_ALERT_2_G Short Intra D-H..H-X       H7       ..H77E             1.88 Ang.
                                                    x,y,z  =      1_555 Check
PLAT414_ALERT_2_G Short Intra D-H..H-X       H7       ..H19A             1.43 Ang.
                                                    x,y,z  =      1_555 Check
```

```
PLAT414_ALERT_2_G Short Intra D-H..H-X       H7       ..H19C            2.11 Ang.
                                                x,y,z  =      1_555 Check
PLAT793_ALERT_4_G Model has Chirality at C12         (Centro SPGR)          S Verify
PLAT793_ALERT_4_G Model has Chirality at C16         (Centro SPGR)          S Verify
PLAT793_ALERT_4_G Model has Chirality at C20         (Centro SPGR)          R Verify
PLAT793_ALERT_4_G Model has Chirality at C26         (Centro SPGR)          R Verify
PLAT793_ALERT_4_G Model has Chirality at C30         (Centro SPGR)          R Verify
PLAT793_ALERT_4_G Model has Chirality at C48         (Centro SPGR)          S Verify
PLAT860_ALERT_3_G Number of Least-Squares Restraints .............        118 Note
PLAT913_ALERT_3_G Missing # of Very Strong Reflections in FCF ....          2 Note
PLAT933_ALERT_2_G Number of OMIT Records in Embedded .res File ...          9 Note
PLAT941_ALERT_3_G Average HKL Measurement Multiplicity ...........        2.9 Low
PLAT978_ALERT_2_G Number C-C Bonds with Positive Residual Density.          0 Info
```

```
  0 ALERT level A = Most likely a serious problem - resolve or explain
  3 ALERT level B = A potentially serious problem, consider carefully
 22 ALERT level C = Check. Ensure it is not caused by an omission or oversight
 36 ALERT level G = General information/check it is not something unexpected

  5 ALERT type 1 CIF construction/syntax error, inconsistent or missing data
 21 ALERT type 2 Indicator that the structure model may be wrong or deficient
 11 ALERT type 3 Indicator that the structure quality may be low
 23 ALERT type 4 Improvement, methodology, query or suggestion
  1 ALERT type 5 Informative message, check
```

It is advisable to attempt to resolve as many as possible of the alerts in all categories. Often the minor alerts point to easily fixed oversights, errors and omissions in your CIF or refinement strategy, so attention to these fine details can be worthwhile. In order to resolve some of the more serious problems it may be necessary to carry out additional measurements or structure refinements. However, the purpose of your study may justify the reported deviations and the more serious of these should normally be commented upon in the discussion or experimental section of a paper or in the "special_details" fields of the CIF. checkCIF was carefully designed to identify outliers and unusual parameters, but every test has its limitations and alerts that are not important in a particular case may appear. Conversely, the absence of alerts does not guarantee there are no aspects of the results needing attention. It is up to the individual to critically assess their own results and, if necessary, seek expert advice.

Publication of your CIF in IUCr journals

A basic structural check has been run on your CIF. These basic checks will be run on all CIFs submitted for publication in IUCr journals (*Acta Crystallographica*, *Journal of Applied Crystallography*, *Journal of Synchrotron Radiation*); however, if you intend to submit to *Acta Crystallographica Section C* or *E* or *IUCrData*, you should make sure that full publication checks are run on the final version of your CIF prior to submission.

Publication of your CIF in other journals

Please refer to the *Notes for Authors* of the relevant journal for any special instructions relating to CIF submission.

PLATON version of 13/07/2021; check.def file version of 13/07/2021

Datablock 1 - ellipsoid plot

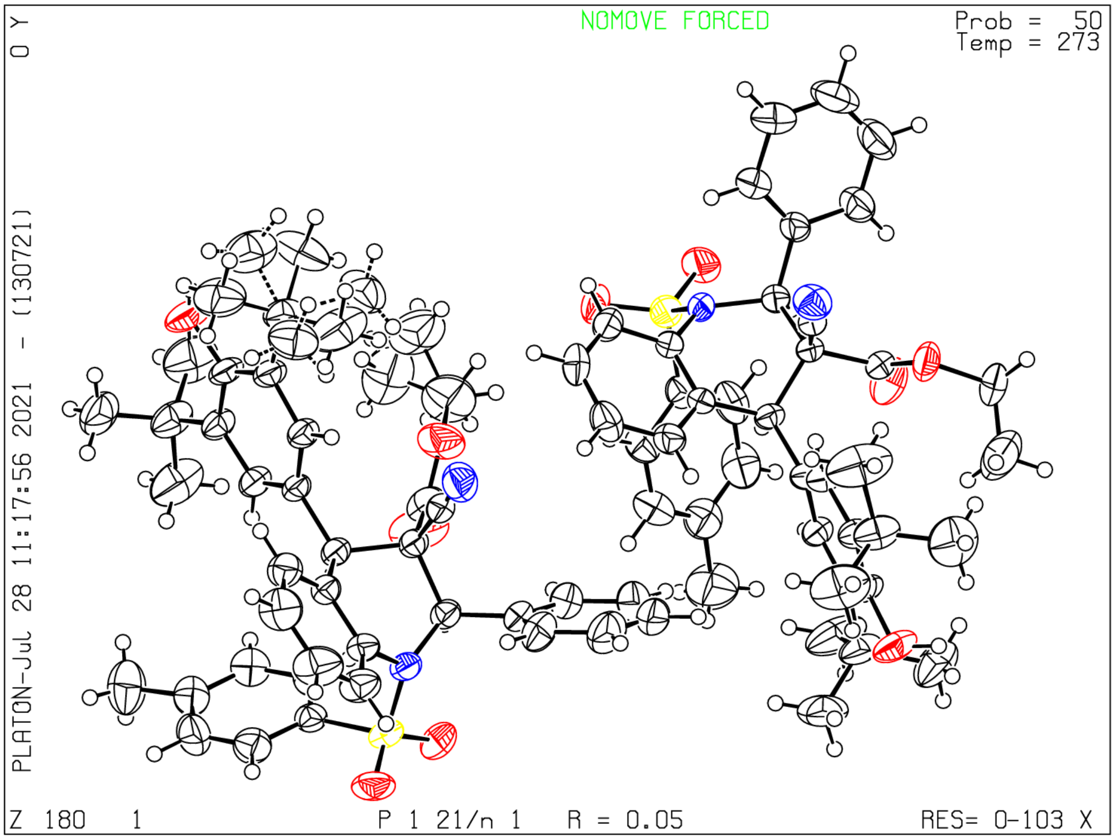

Supplement: Supplementary file 1 [file DataSheet2.PDF]
