# Supplementary material for: Highly Diastereoselective Synthesis of Tetrahydroquinoline Derivatives via [4 + 2] Annulation of Ortho-Tosylaminophenyl-Substituted Para-Quinone Methides and Cyanoalkenes
Source: Front Chem. 2021 Nov 3;9:764866. doi: 10.3389/fchem.2021.764866 (PMC8595915; doi:10.3389/fchem.2021.764866)
Supplement: Supplementary file 2 [file DataSheet3.PDF]

Highly Diastereoselective Synthesis of Tetrahydroquinoline Derivatives via [4 + 2] Annulation of *Ortho*-Tosylaminophenyl-Substituted *Para*-Quinone Methides and Cyanoalkenes

Taiwei Dong[1*], Peifeng Wei [1], Min Li[1], Feng Gao[1], Yuan Qin[2*]

[1] College of Pharmacy, Shanxi University of Chinese Medicine, Shiji Road, Qindu District, Xianyang, Shanxi 712046, China
[2] State Key Laboratory of Military Stomatology & National Clinical Research Center for Oral Diseases & Shanxi International Joint Research Center for Oral Diseases, Fourth Military Medical University, Xi'an, 710032, China

*** Correspondence:**
Taiwei Dong zhongxidong123@sina.com

Yuan Qin qbazinga@163.com

Supporting Material

Table of Contents

NMR spectra of products **3** 2

NMR spectra of products **5**, **7** and **8** 29

Crystal data of prodcut **3a** and **3z** 35

NMR spectra of products 3

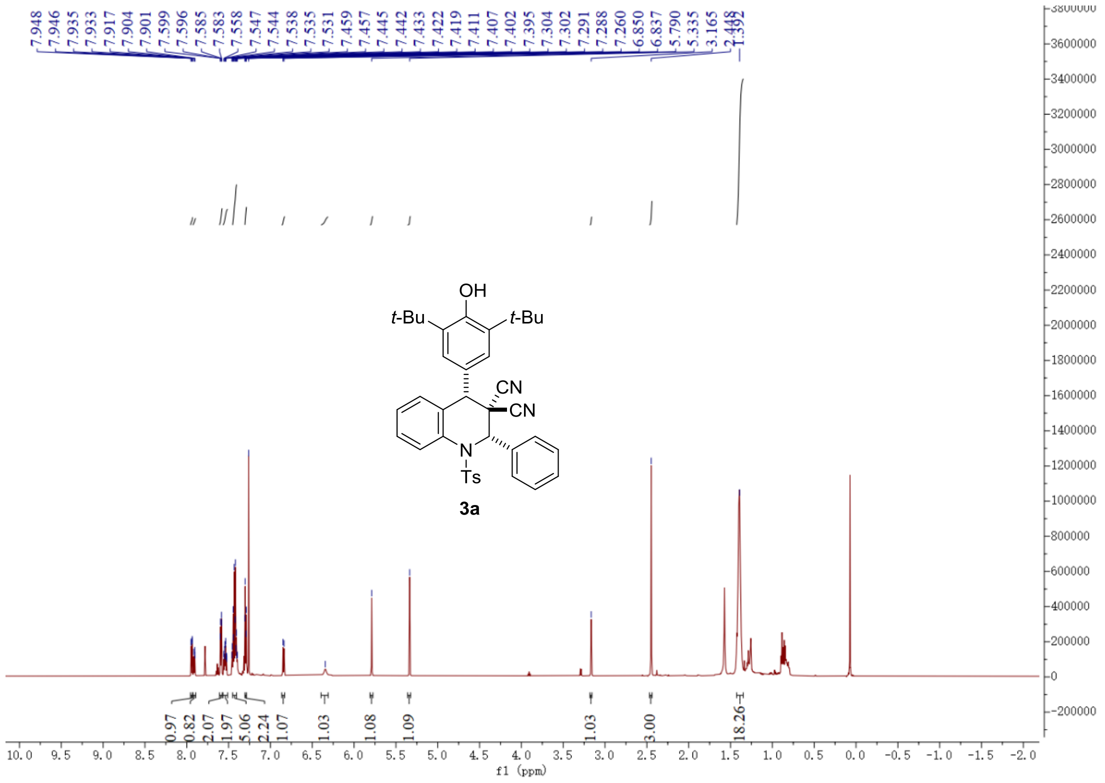

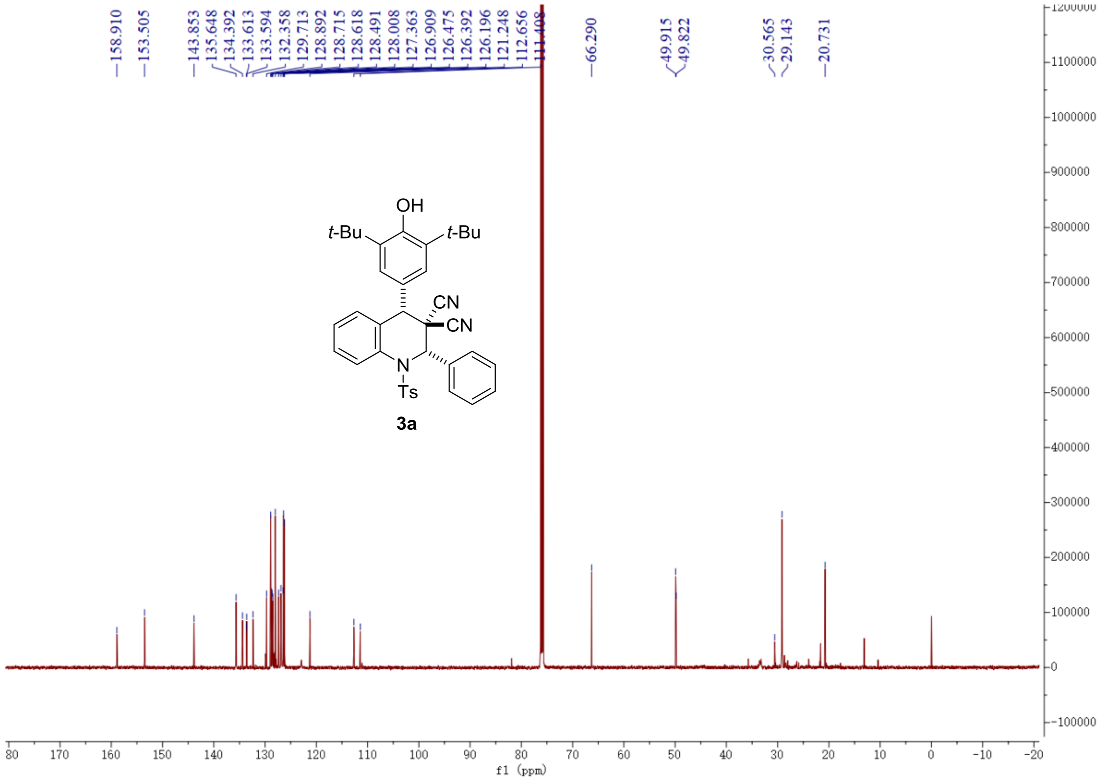

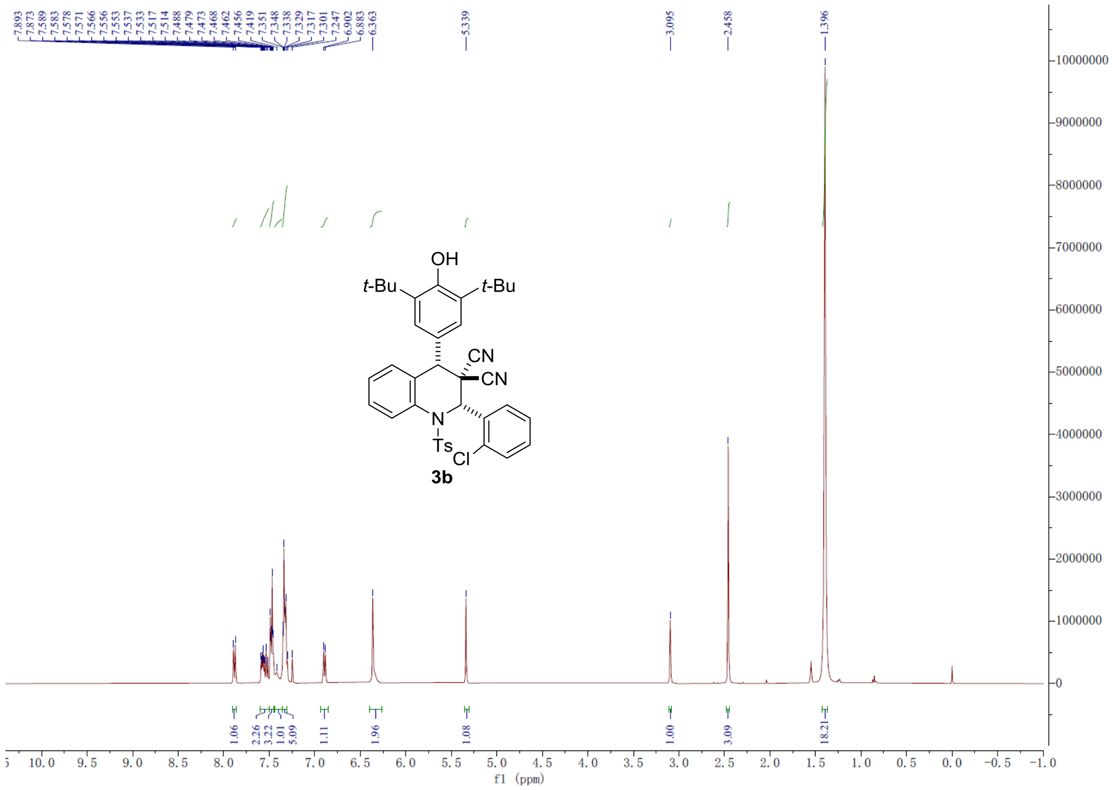
7.893
7.873
7.589
7.583
7.578
7.571
7.566
7.556
7.553
7.537
7.533
7.517
7.514
7.488
7.479
7.473
7.468
7.462
7.456
7.419
7.351
7.348
7.338
7.329
7.317
7.301
7.247
6.902
6.883
6.363
5.339
3.095
2.458
1.396
OH
t-Bu
t-Bu
CN
CN
N
Ts
Cl
3b
1.06
2.26
3.22
1.01
5.09
1.11
1.96
1.08
1.00
3.09
18.21
f1 (ppm)

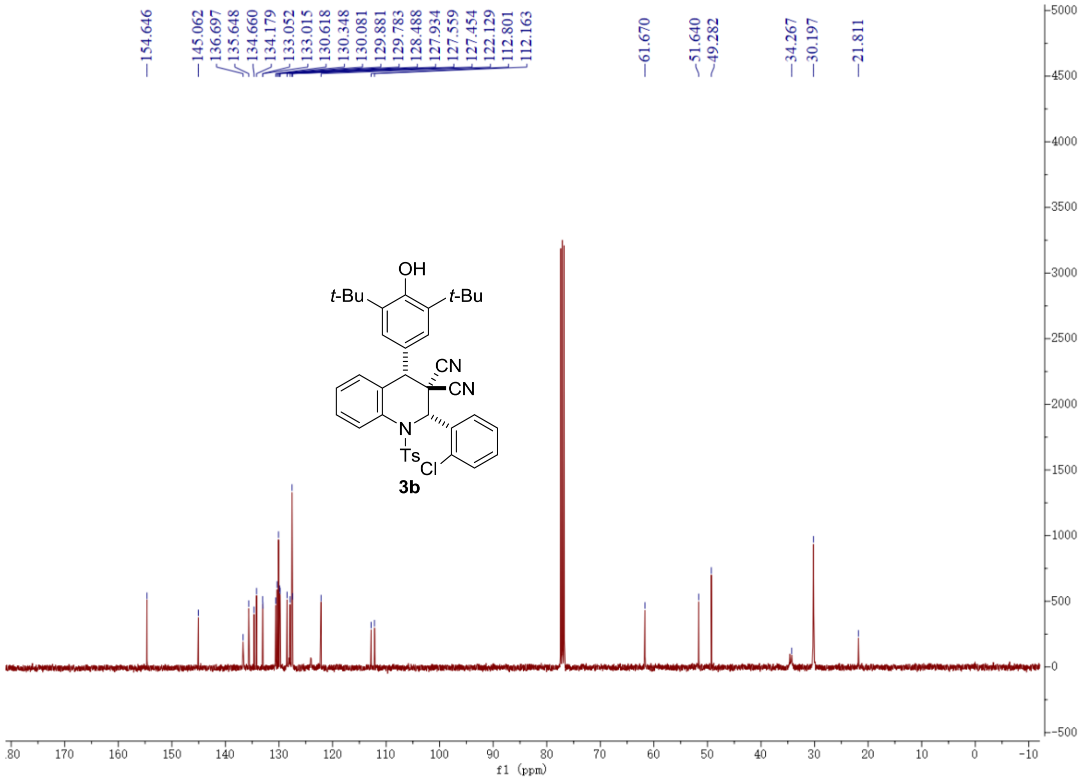
154.646
145.062
136.697
135.648
134.660
134.179
133.052
133.015
130.618
130.348
130.081
129.881
129.783
128.488
127.934
127.559
127.454
122.129
112.801
112.163
61.670
51.640
49.282
34.267
30.197
21.811
OH
t-Bu
t-Bu
CN
CN
N
Ts
Cl
3b
f1 (ppm)

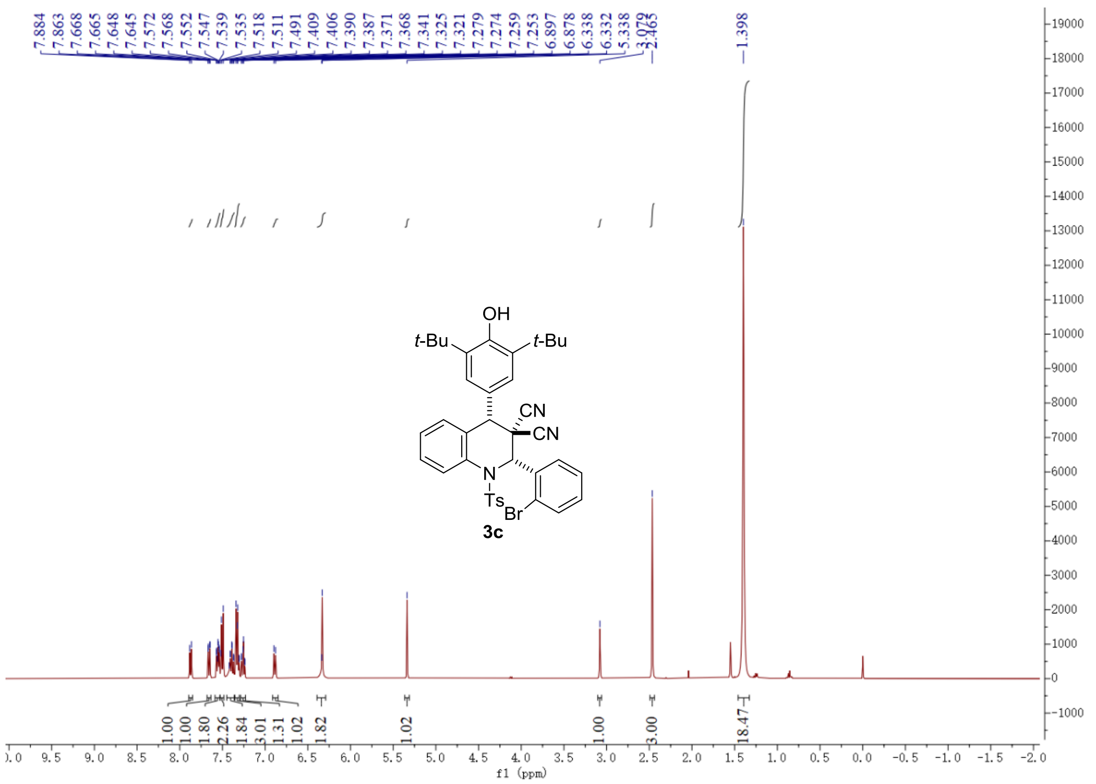

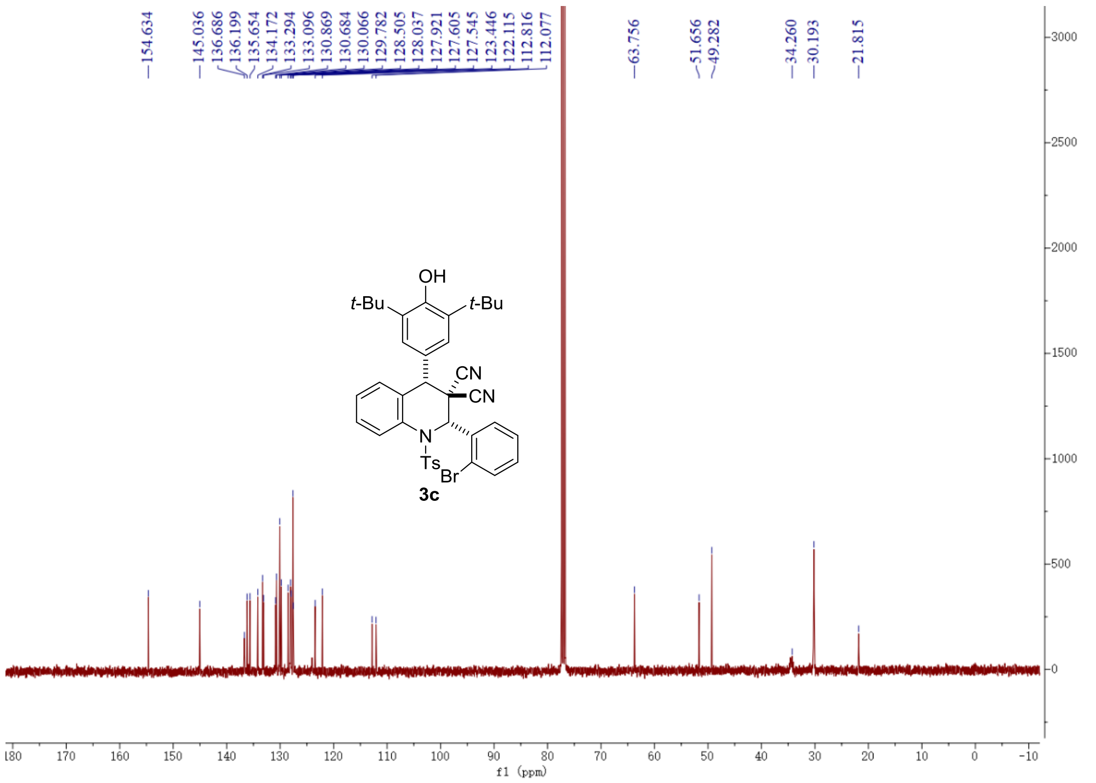

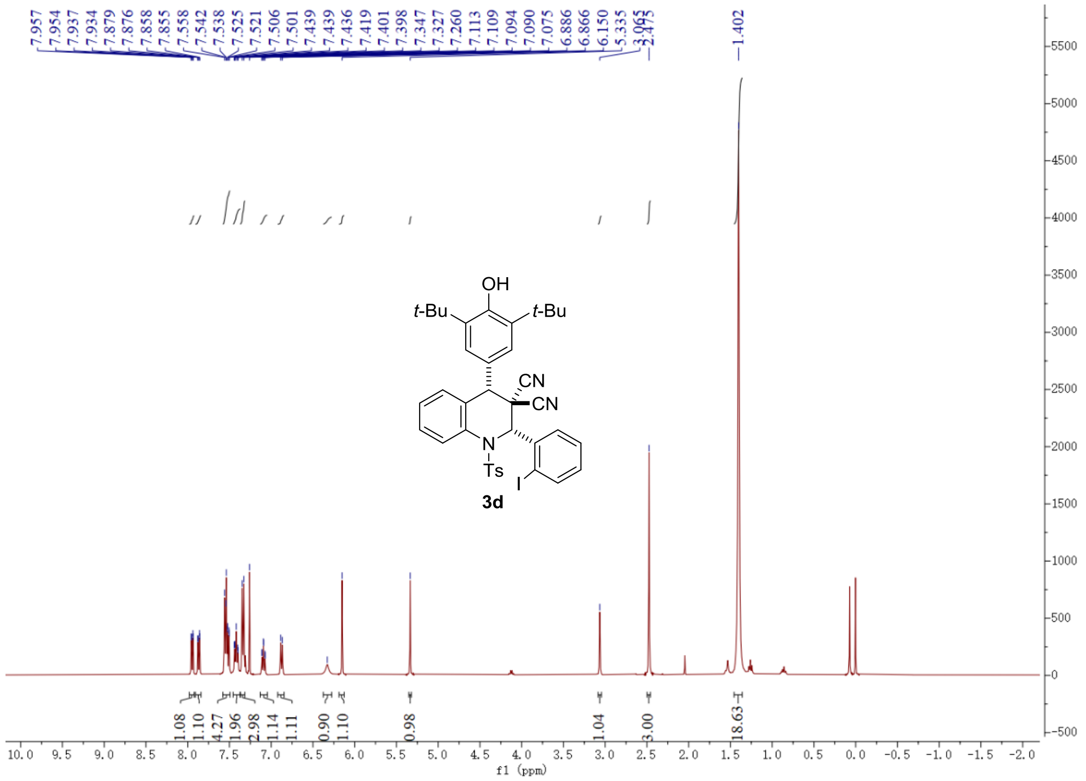

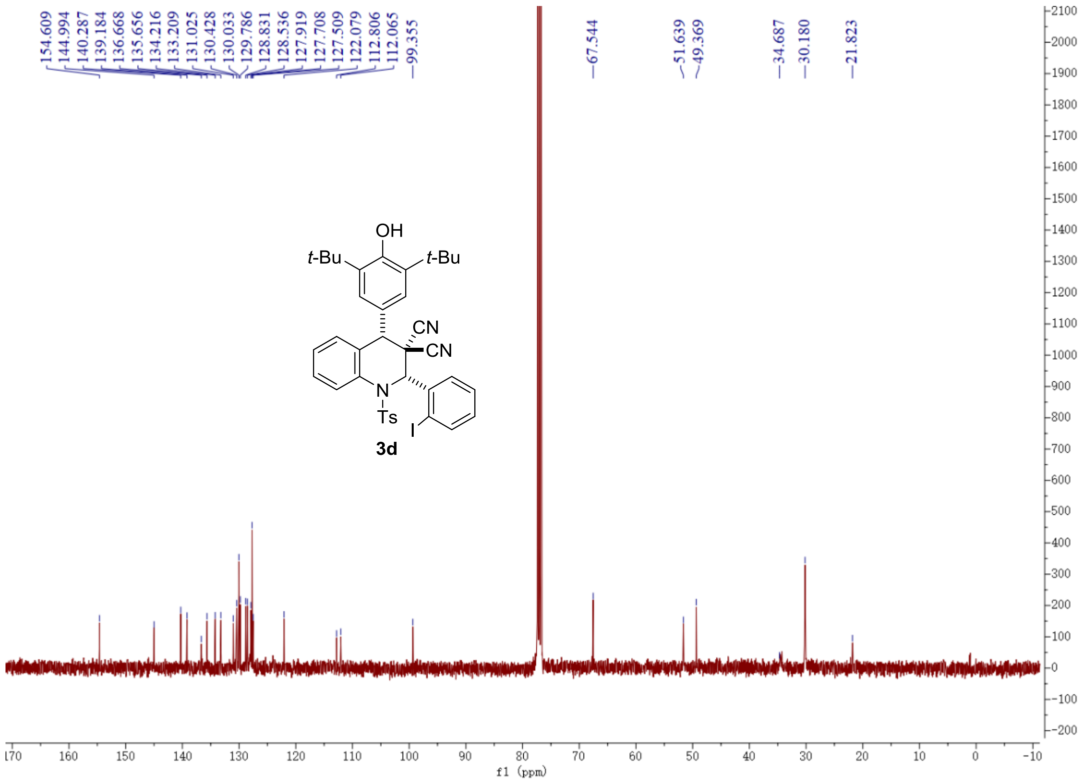

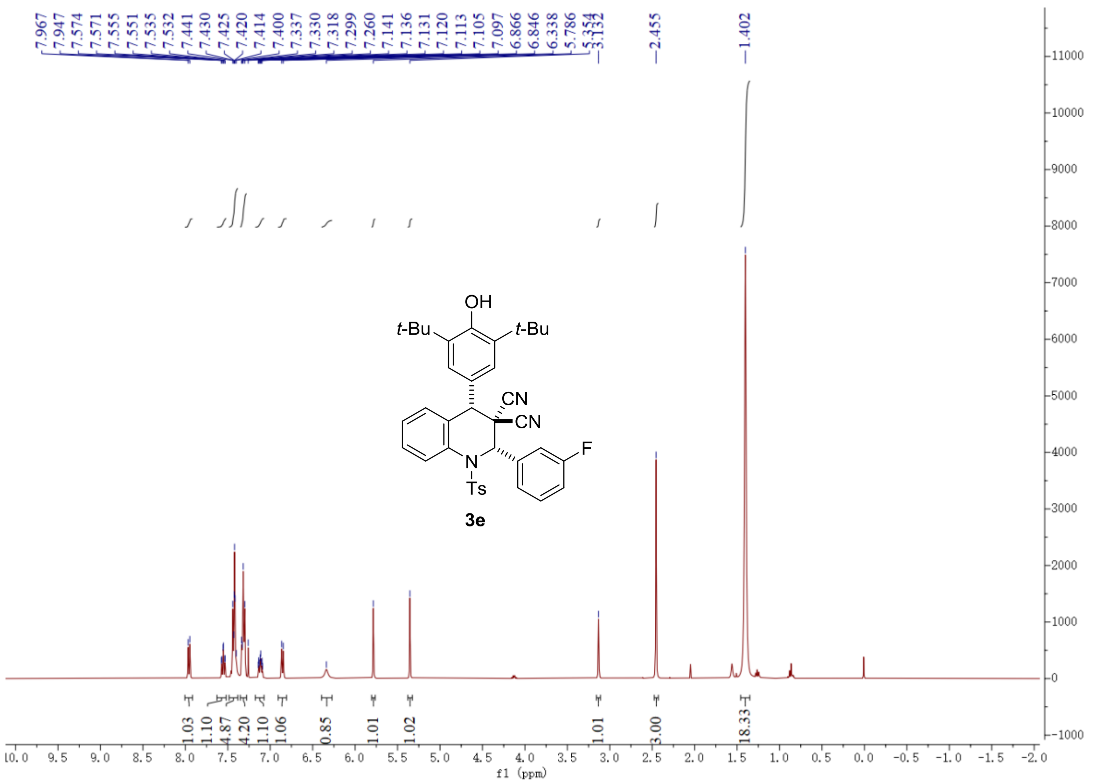

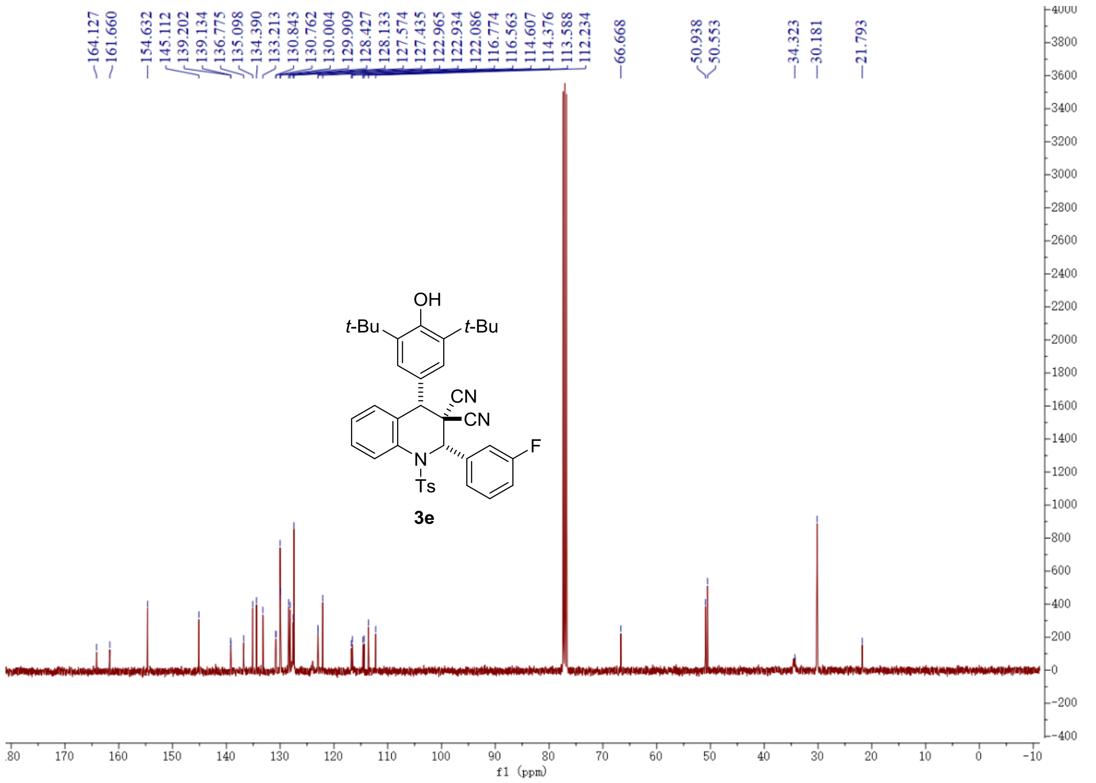

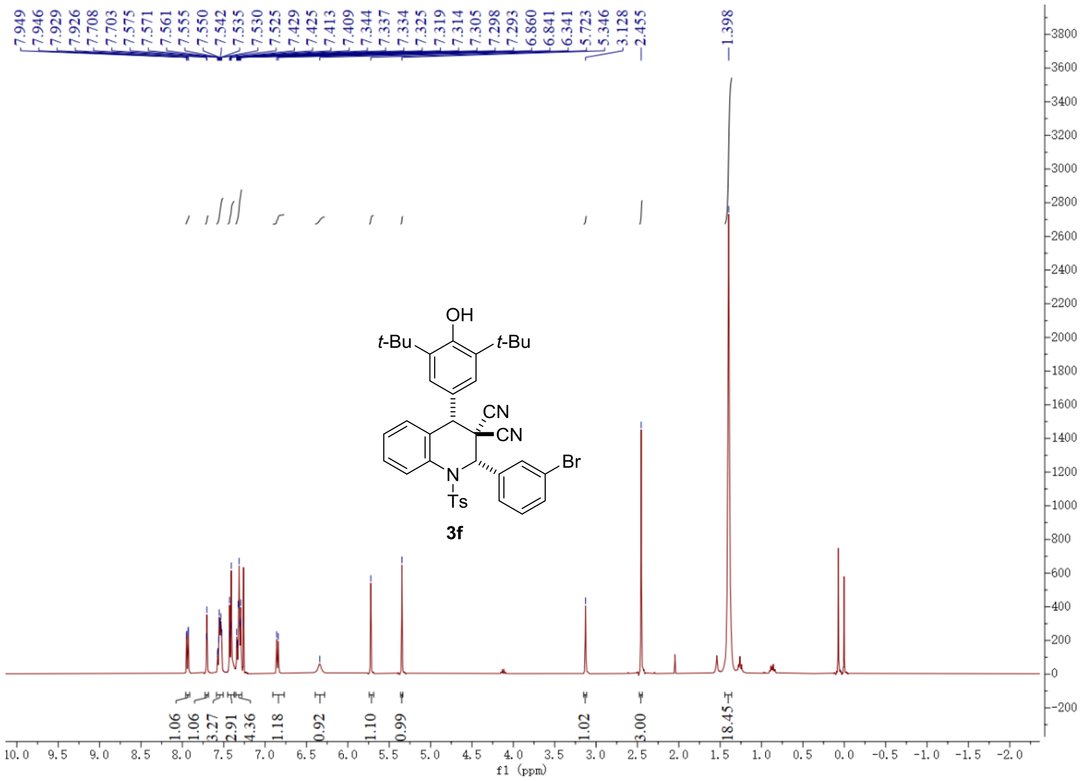

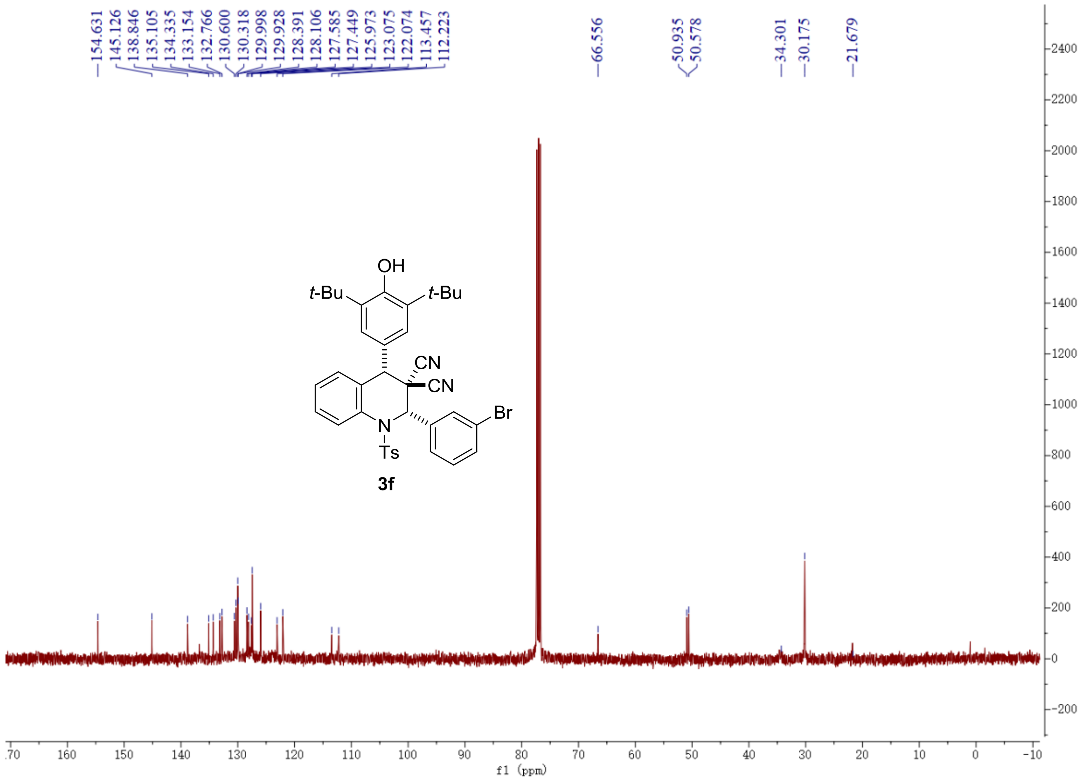

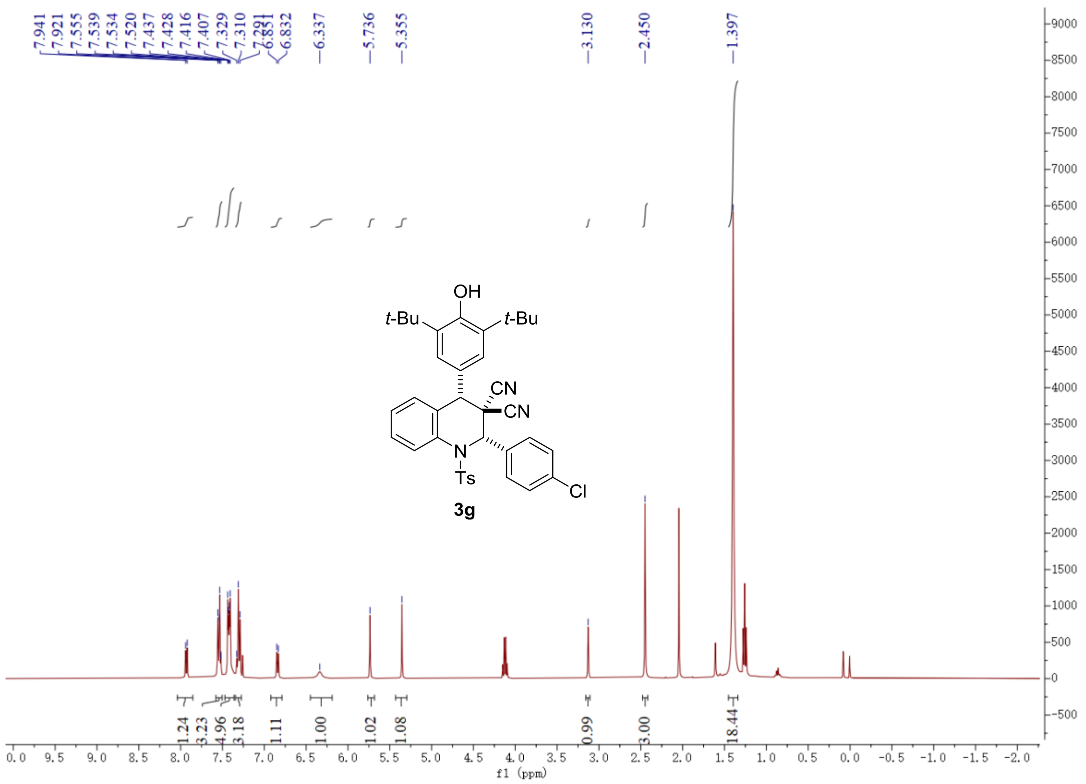

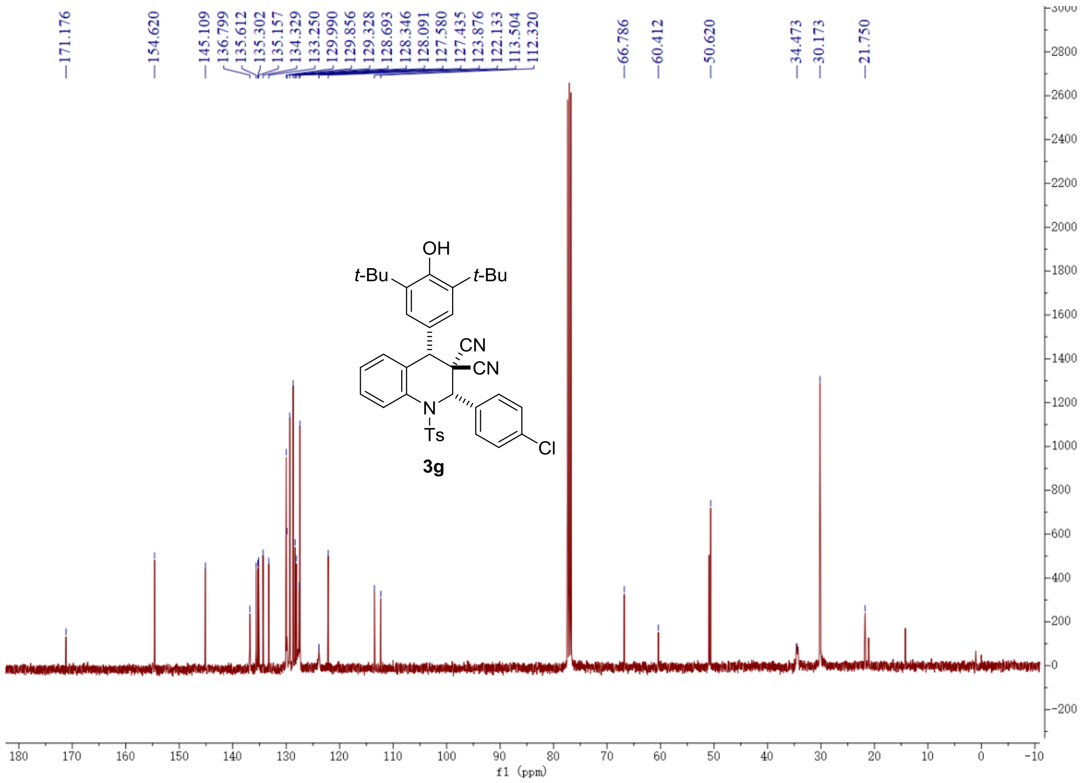

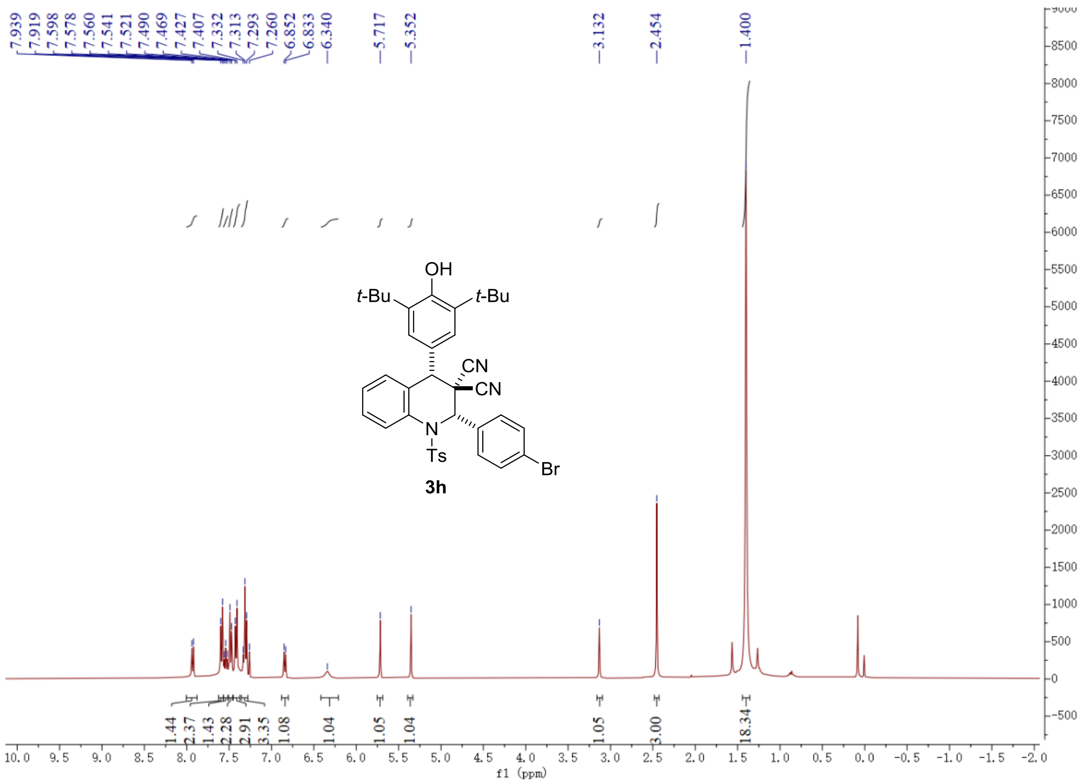
7.939
7.919
7.598
7.578
7.560
7.541
7.521
7.490
7.469
7.427
7.407
7.332
7.313
7.293
7.260
6.852
6.833
6.340
5.717
5.352
3.132
2.454
1.400
OH
t-Bu
t-Bu
CN
CN
N
Ts
Br
3h
1.44
2.37
1.43
2.28
2.91
3.35
1.08
1.04
1.05
1.04
1.05
3.00
18.34
f1 (ppm)

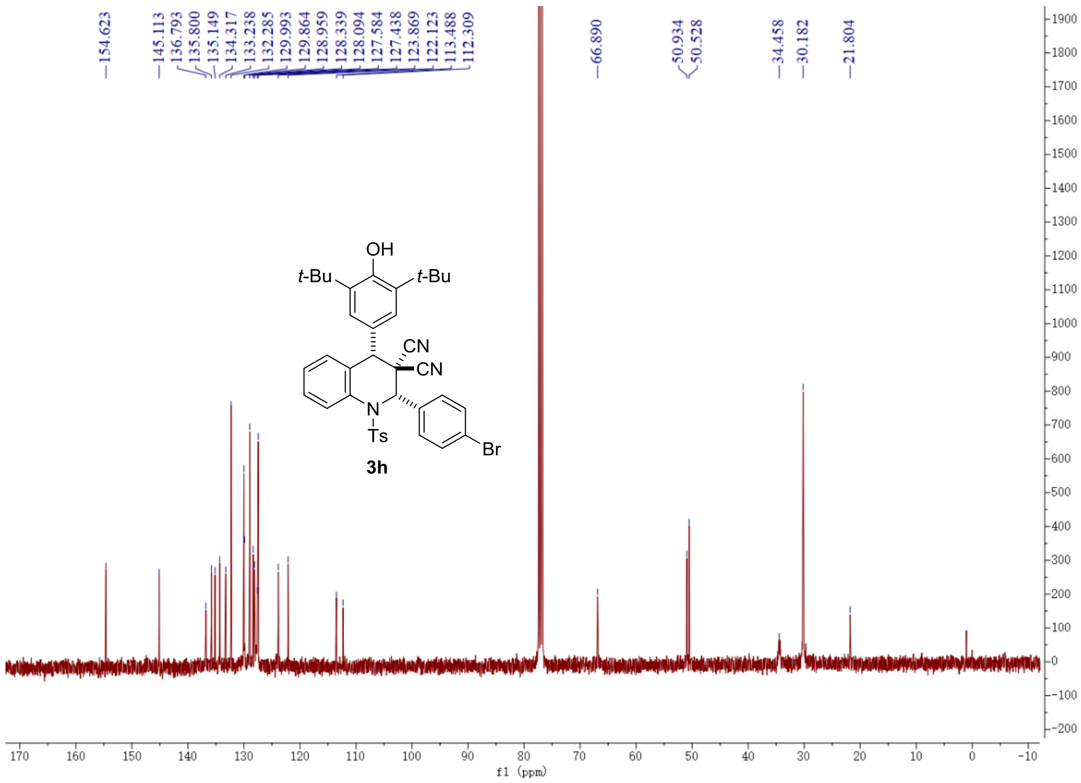
154.623
145.113
136.793
135.800
135.149
134.317
133.238
132.285
129.993
129.864
128.959
128.339
128.094
127.584
127.438
123.869
122.123
113.488
112.309
66.890
50.934
50.528
34.458
30.182
21.804
OH
t-Bu
t-Bu
CN
CN
N
Ts
Br
3h
f1 (ppm)

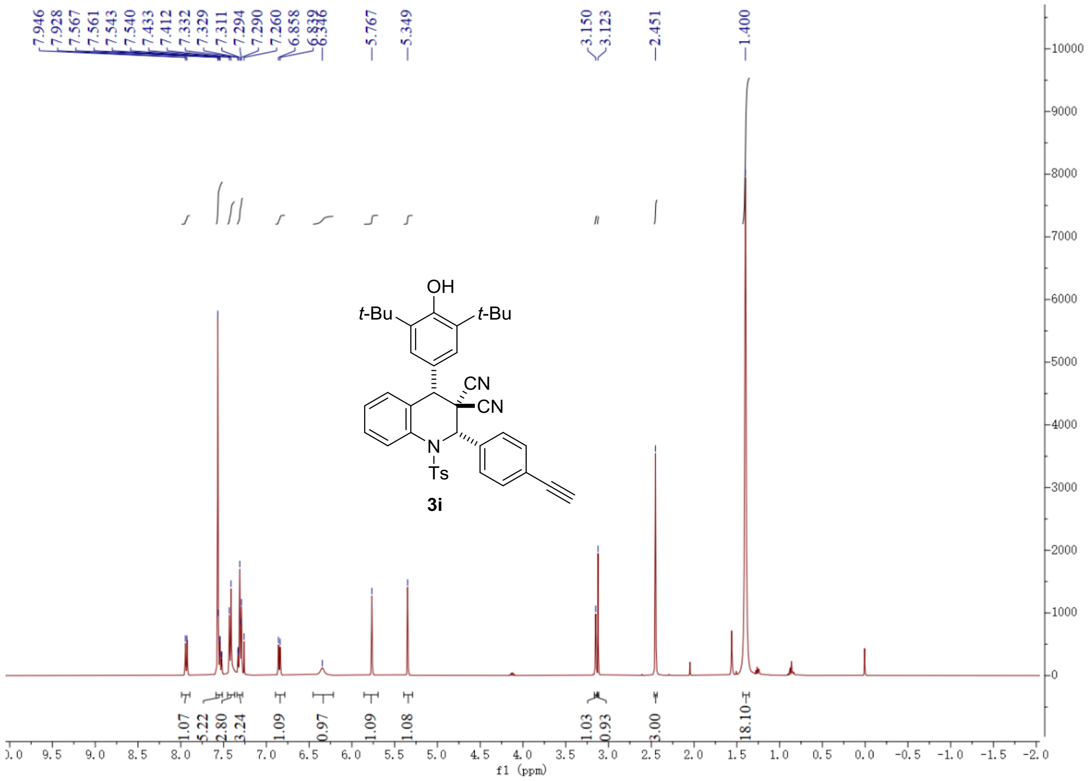
7.946
7.928
7.567
7.561
7.543
7.540
7.433
7.412
7.332
7.329
7.311
7.294
7.290
7.260
6.858
6.839
6.846
5.767
5.349
3.150
3.123
2.451
1.400
OH
t-Bu
t-Bu
CN
CN
N
Ts
3i
1.07
5.22
2.80
3.24
1.09
0.97
1.09
1.08
1.03
0.93
3.00
18.10
f1 (ppm)

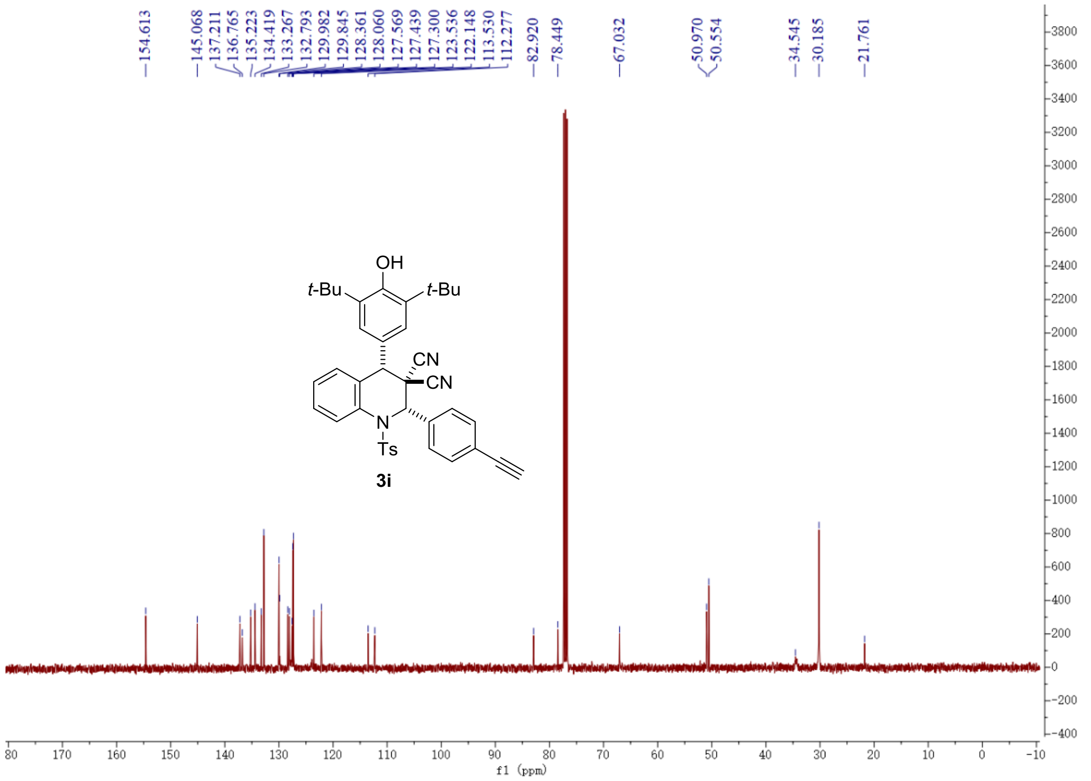
154.613
145.068
137.211
136.765
135.223
134.419
133.267
132.793
129.982
129.845
128.361
128.060
127.569
127.439
127.300
123.536
122.148
113.530
112.277
82.920
78.449
67.032
50.970
50.554
34.545
30.185
21.761
OH
t-Bu
t-Bu
CN
CN
N
Ts
3i
f1 (ppm)

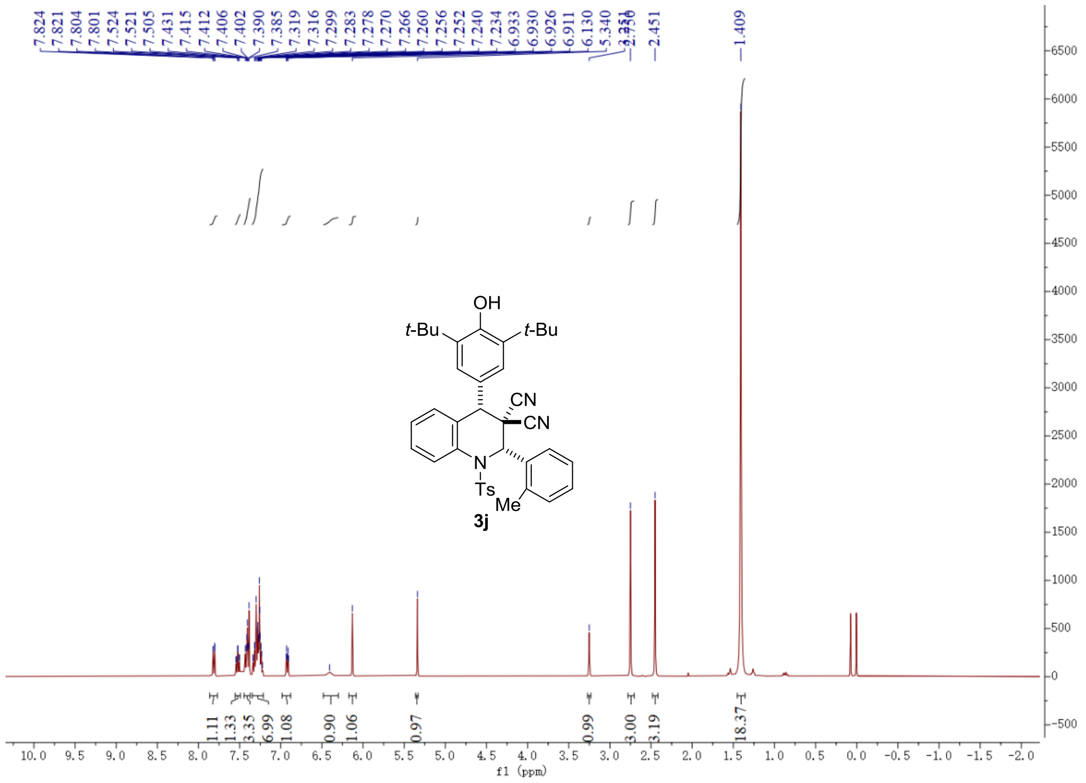

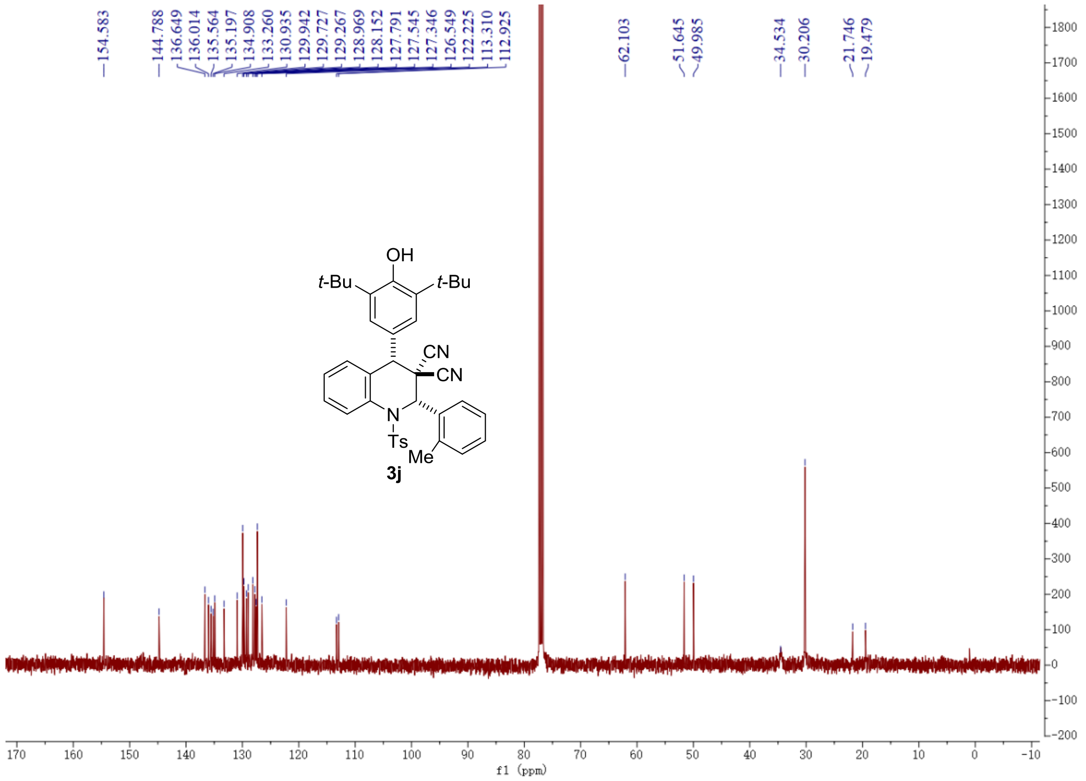

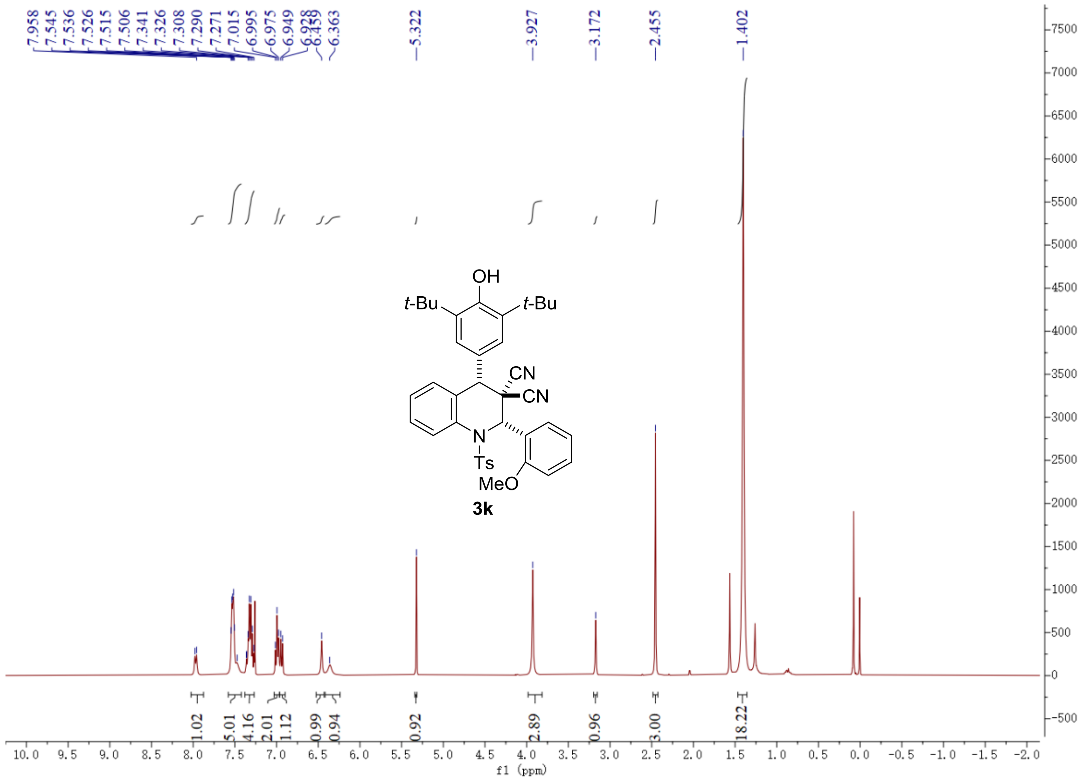
7.958
7.545
7.536
7.526
7.515
7.506
7.341
7.326
7.308
7.290
7.271
7.015
6.995
6.975
6.949
6.363
5.322
3.927
3.172
2.455
1.402
OH
t-Bu
t-Bu
CN
CN
N
Ts
MeO
3k
1.02
5.01
4.16
2.01
1.12
0.99
0.94
0.92
2.89
0.96
3.00
18.22
f1 (ppm)

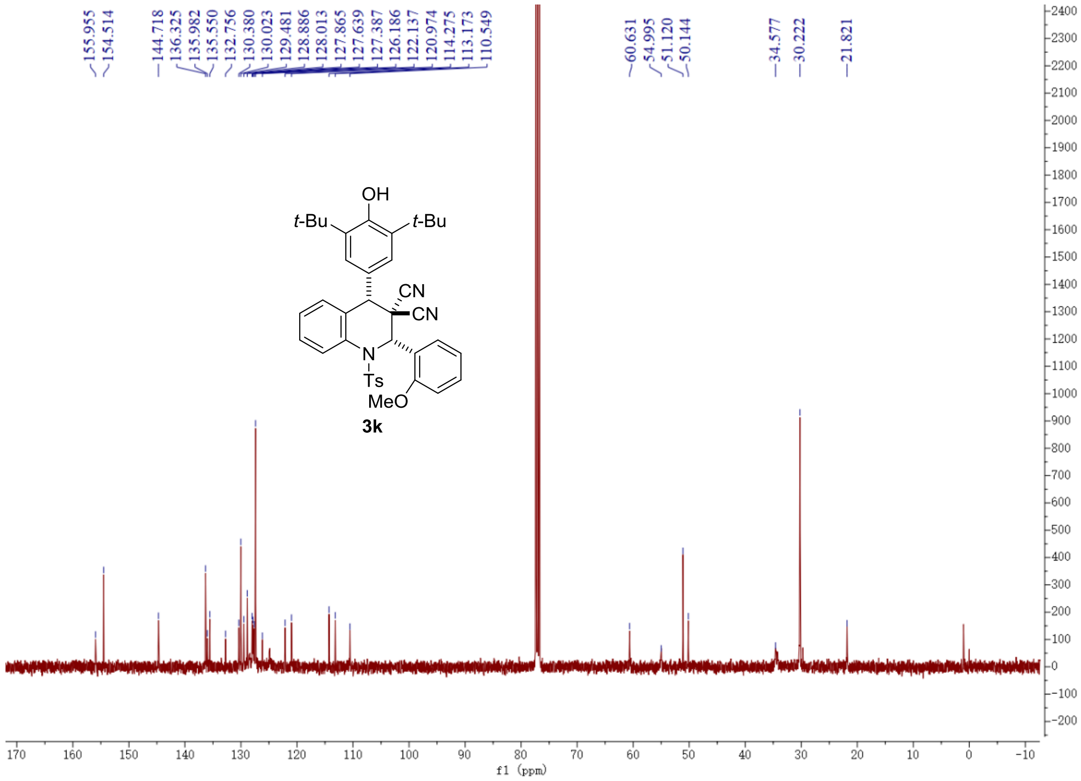
155.955
154.514
144.718
136.325
135.982
135.550
132.756
130.380
130.023
129.481
128.886
128.013
127.865
127.639
127.387
126.186
122.137
120.974
114.275
113.173
110.549
60.631
54.995
51.120
50.144
34.577
30.222
21.821
OH
t-Bu
t-Bu
CN
CN
N
Ts
MeO
3k
f1 (ppm)

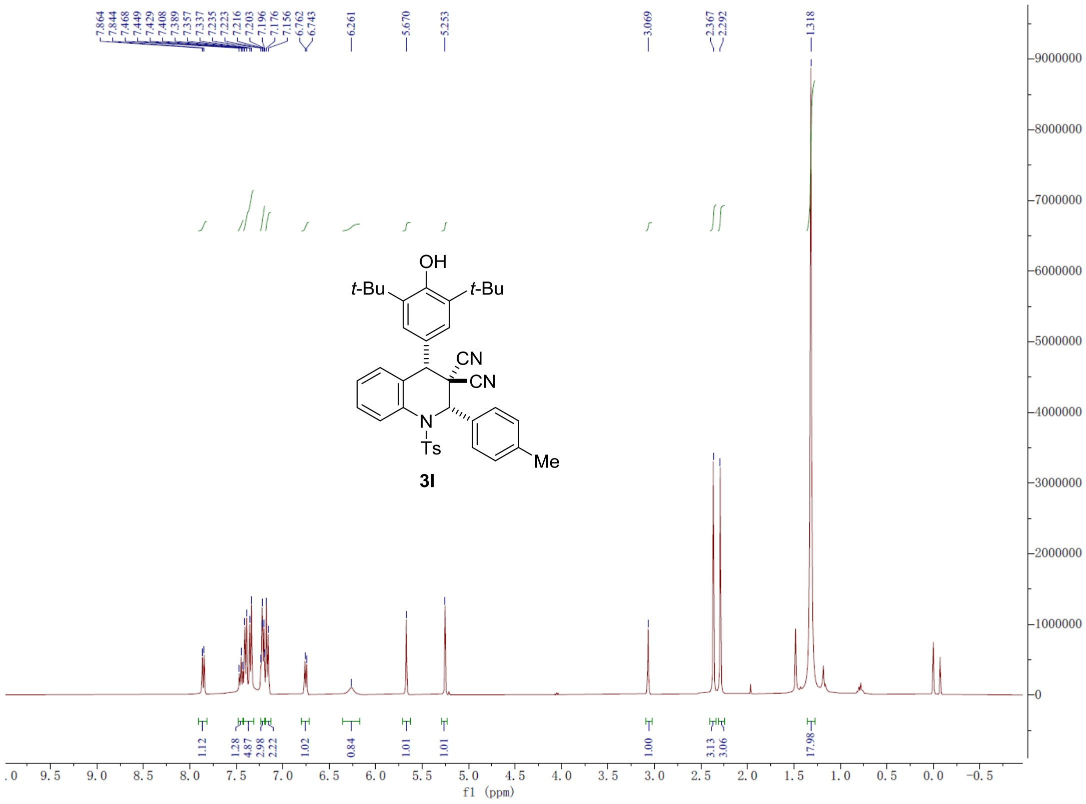

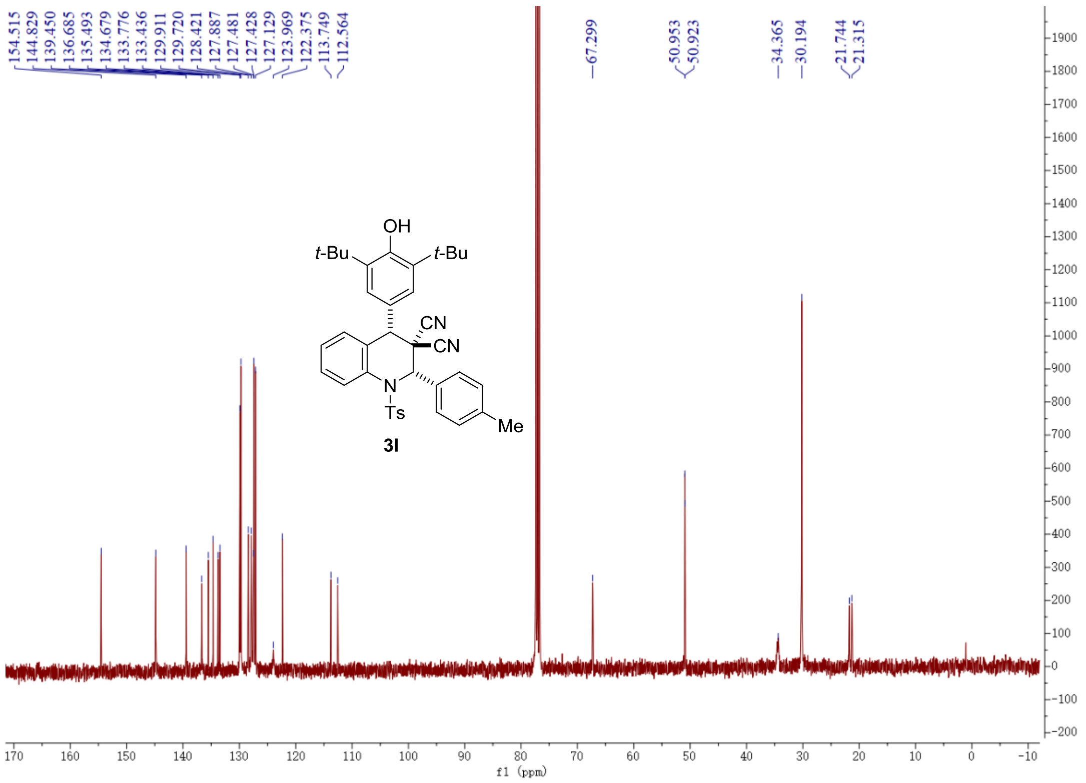

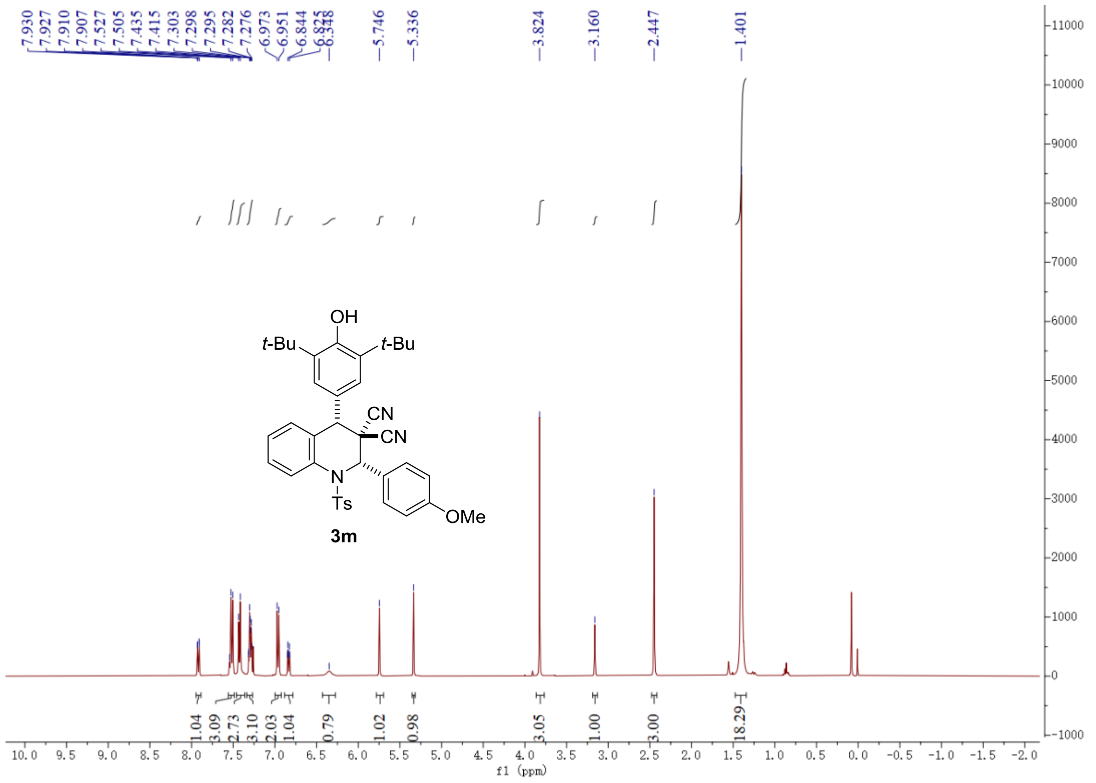
OH
t-Bu
t-Bu
CN
CN
N
Ts
OMe
3m
f1 (ppm)

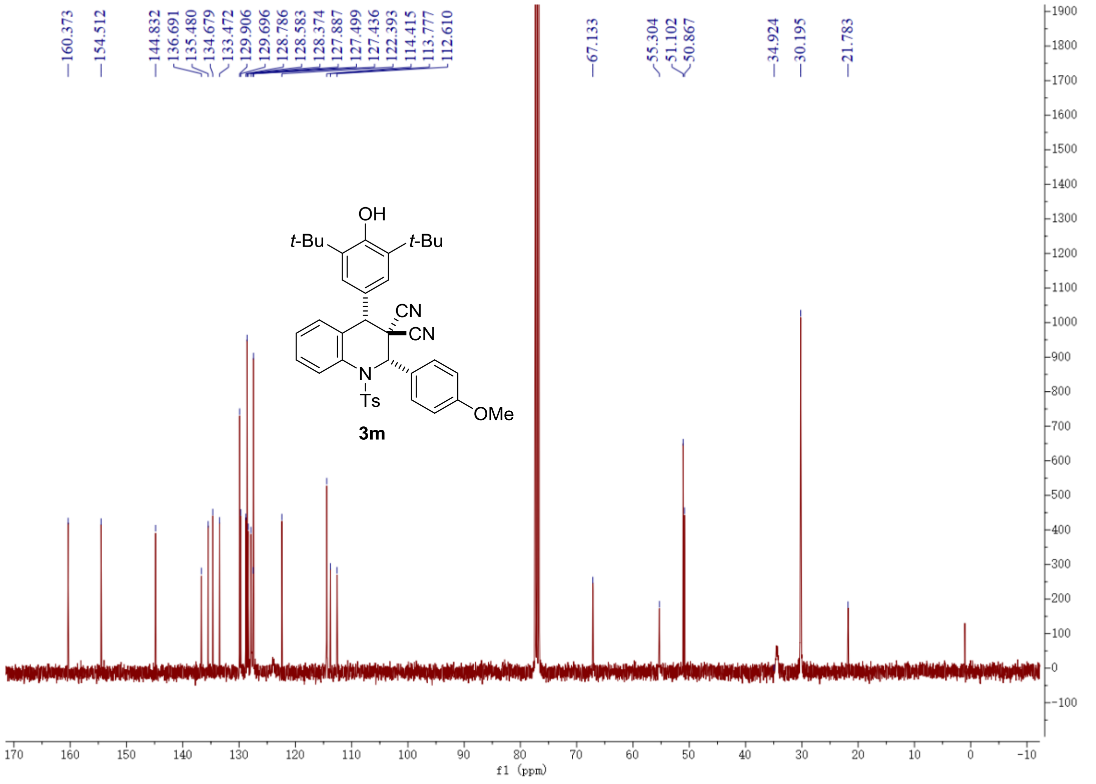
OH
t-Bu
t-Bu
CN
CN
N
Ts
OMe
3m
f1 (ppm)

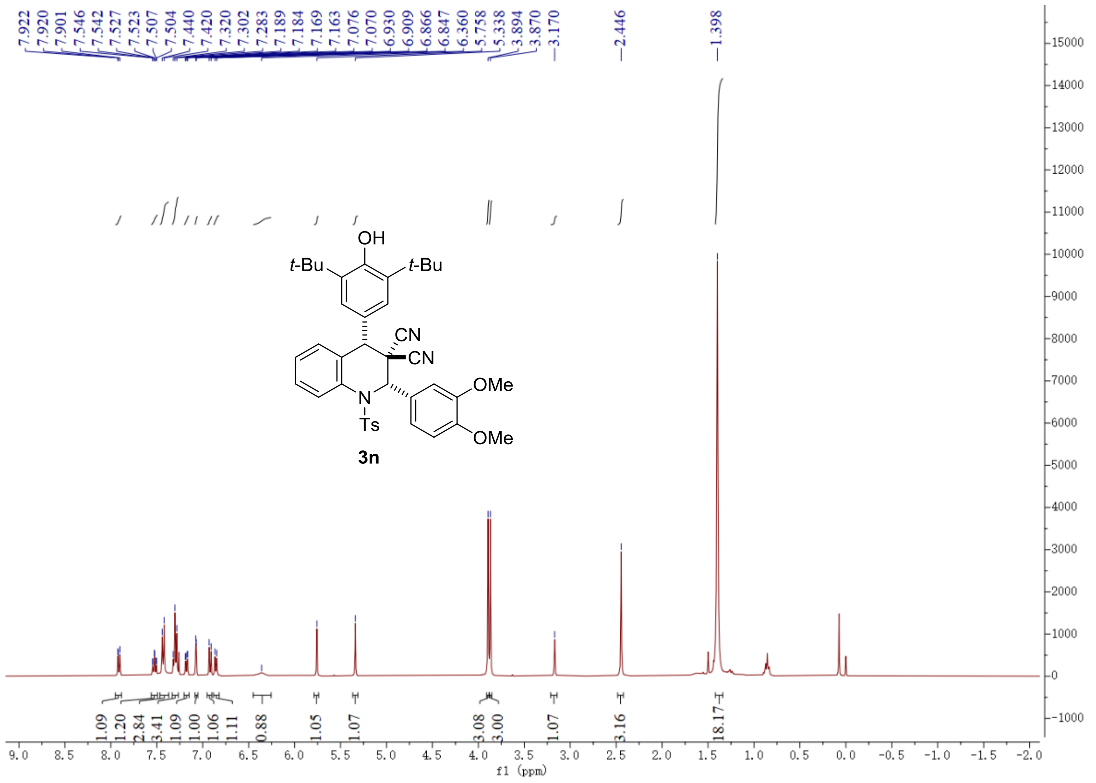
7.922
7.920
7.901
7.546
7.542
7.527
7.523
7.507
7.504
7.440
7.420
7.320
7.302
7.283
7.189
7.184
7.169
7.163
7.076
7.070
6.930
6.909
6.866
6.847
6.360
5.758
5.338
3.894
3.870
3.170
2.446
1.398
OH
t-Bu
t-Bu
CN
CN
N
Ts
OMe
OMe
3n
1.09
1.20
2.84
3.41
1.09
1.00
1.06
1.11
0.88
1.05
1.07
3.08
3.00
1.07
3.16
18.17
f1 (ppm)

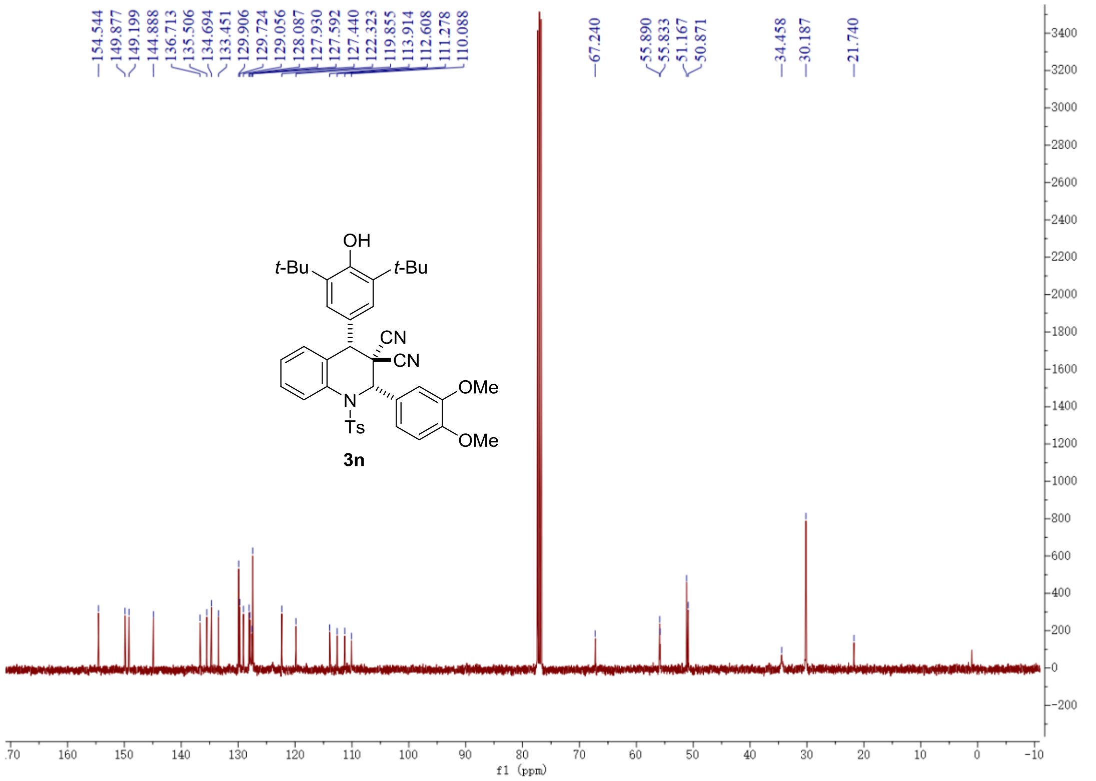
154.544
149.877
149.199
144.888
136.713
135.506
134.694
133.451
129.906
129.724
129.056
128.087
127.930
127.592
127.440
122.323
119.855
113.914
112.608
111.278
110.088
67.240
55.890
55.833
51.167
50.871
34.458
30.187
21.740
OH
t-Bu
t-Bu
CN
CN
N
Ts
OMe
OMe
3n
f1 (ppm)

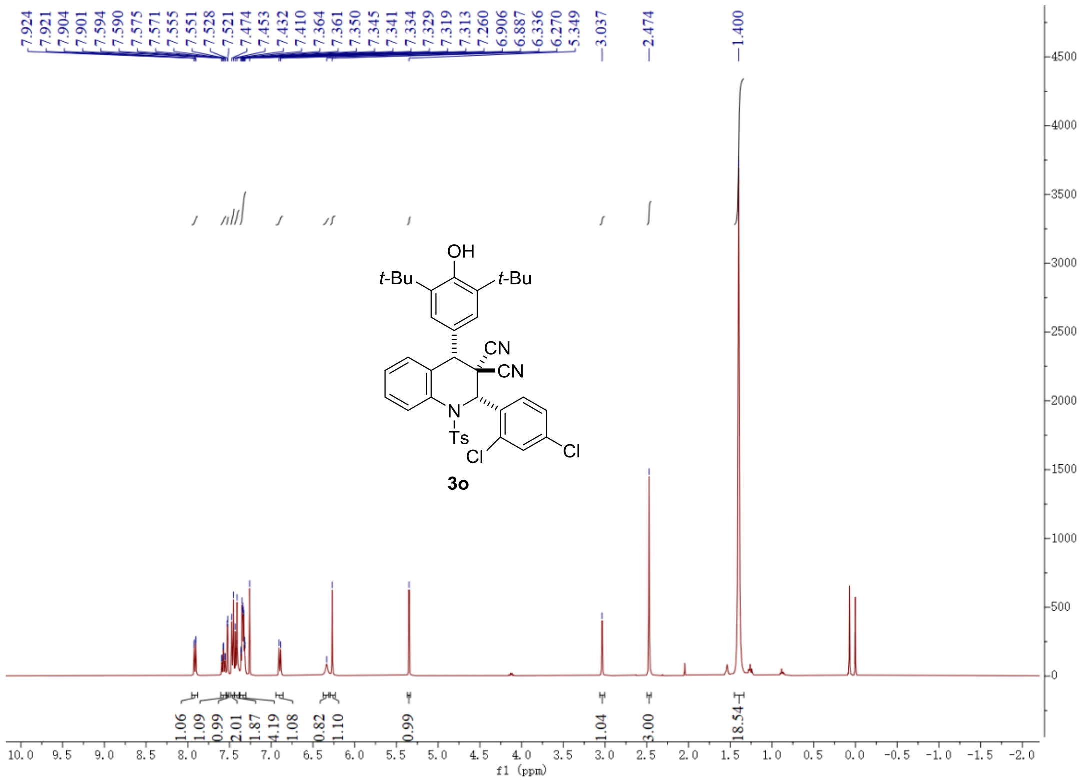
7.924 7.921 7.904 7.901 7.594 7.590 7.575 7.571 7.555 7.551 7.528 7.521 7.474 7.453 7.432 7.410 7.364 7.361 7.350 7.345 7.341 7.334 7.329 7.319 7.313 7.260 6.906 6.887 6.336 6.270 5.349
3.037
2.474
1.400
OH
t-Bu
t-Bu
CN
CN
N
Ts
Cl
Cl
3o
1.06 1.09 0.99 2.01 1.87 4.19 1.08 0.82 1.10 0.99 1.04 3.00 18.54
f1 (ppm)

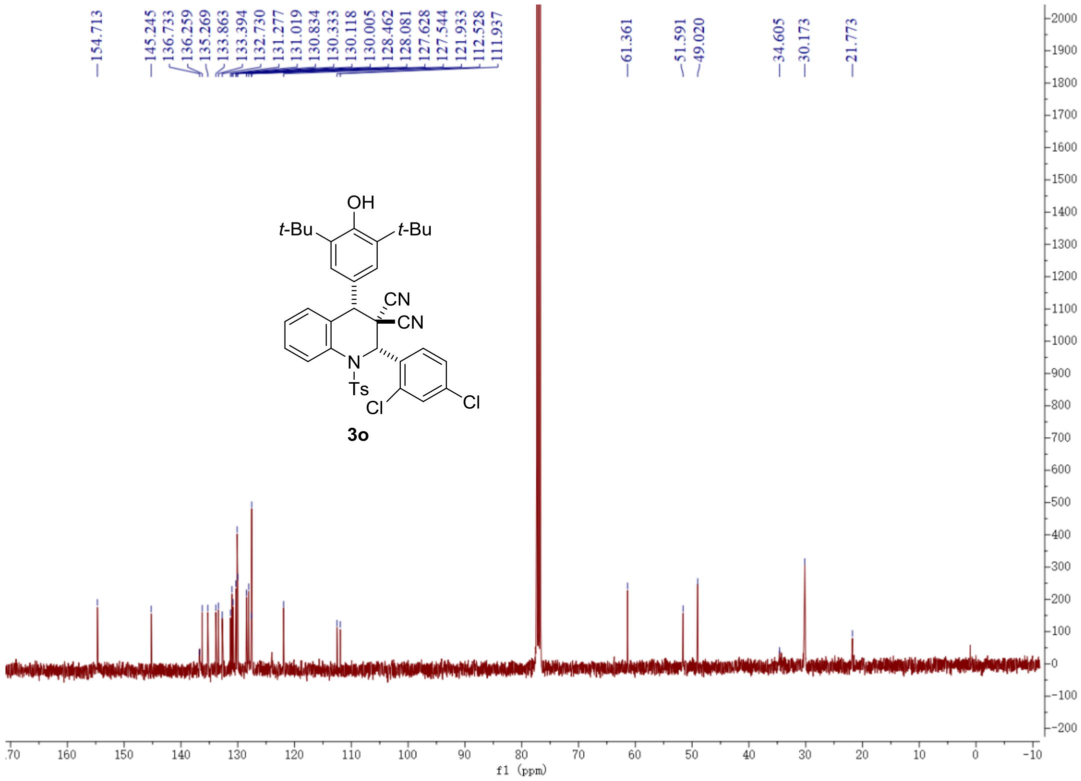
154.713
145.245 136.733 136.259 135.269 133.863 133.394 132.730 131.277 131.019 130.834 130.333 130.118 130.005 128.462 128.081 127.628 127.544 121.933 112.528 111.937
61.361
51.591 49.020
34.605 30.173
21.773
OH
t-Bu
t-Bu
CN
CN
N
Ts
Cl
Cl
3o
f1 (ppm)

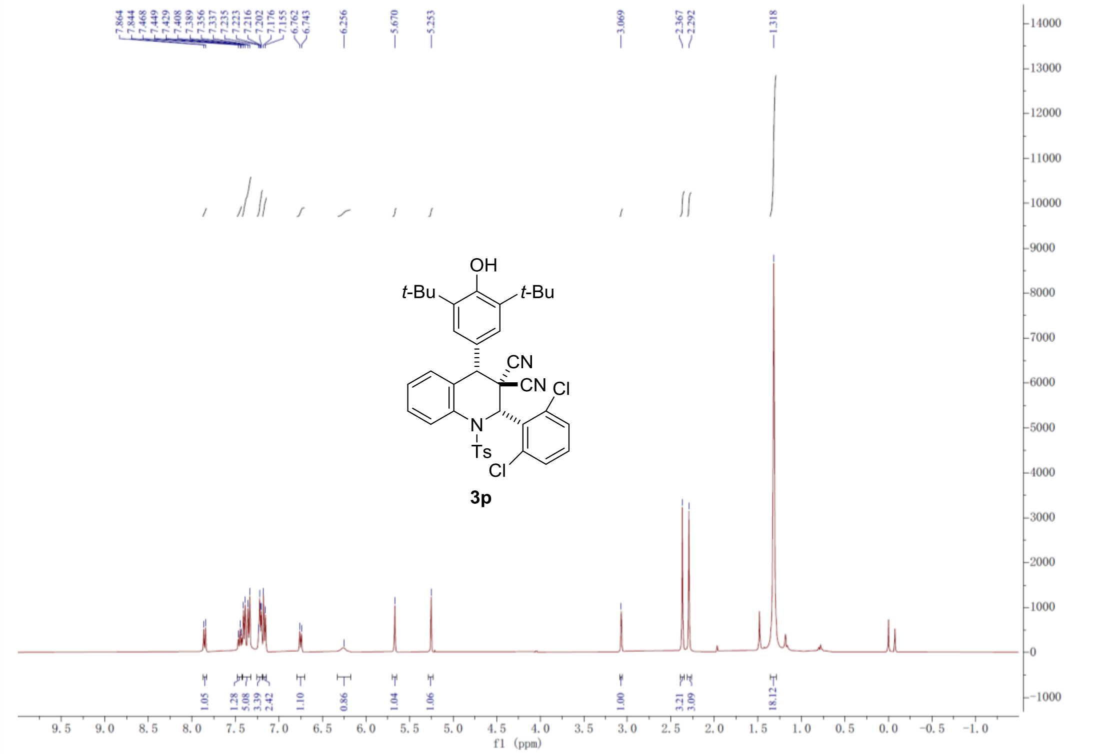

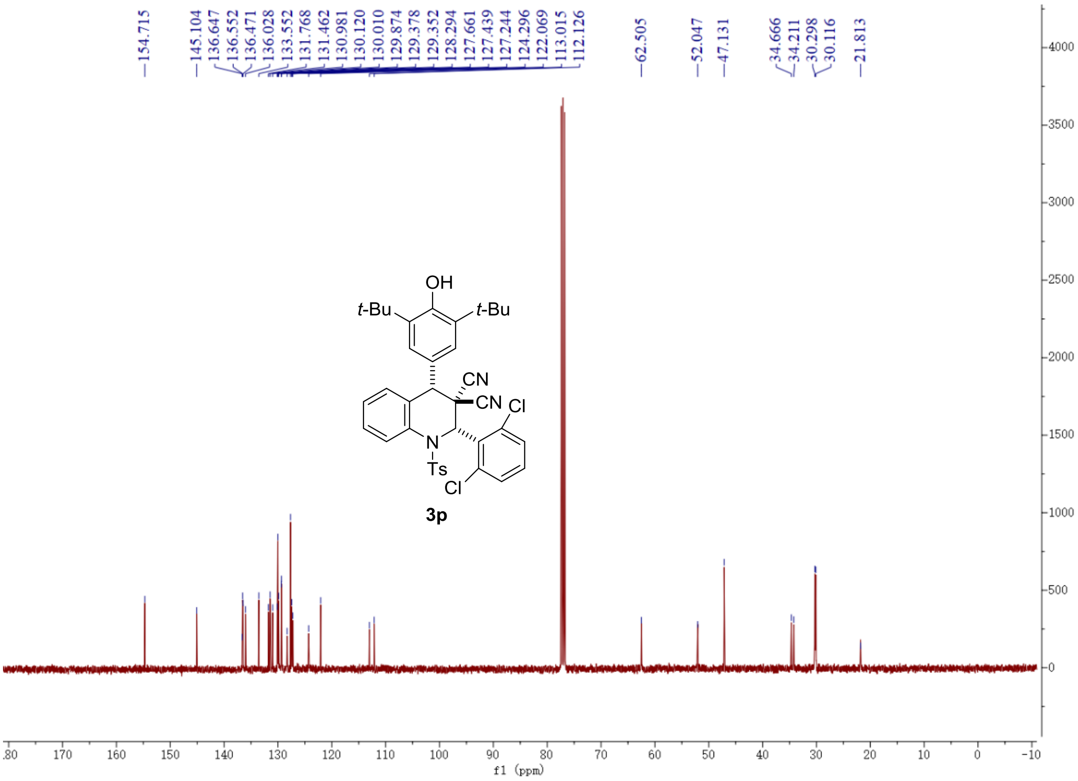

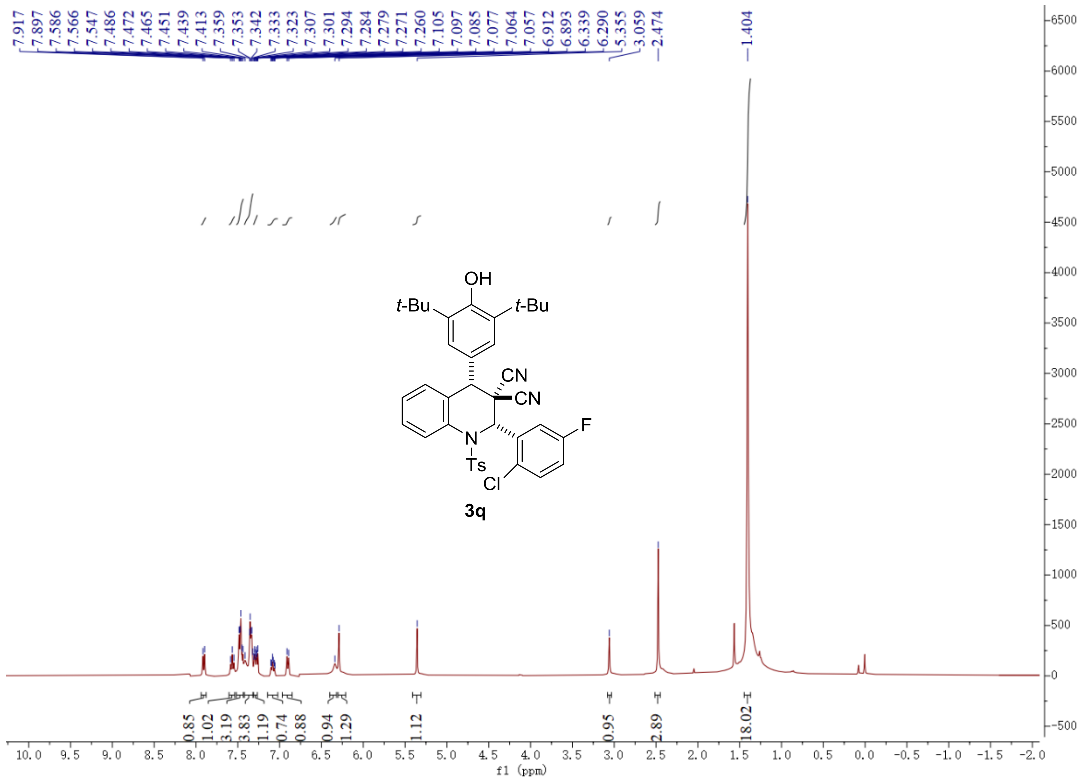
7.917 7.897 7.586 7.566 7.547 7.486 7.472 7.465 7.451 7.439 7.413 7.359 7.353 7.342 7.333 7.323 7.307 7.301 7.294 7.284 7.279 7.271 7.260 7.105 7.097 7.085 7.077 7.064 7.057 6.912 6.893 6.339 6.290 5.355 3.059 2.474
1.404
OH
t-Bu
t-Bu
CN
CN
F
N
Ts
Cl
3q
0.85 1.02 3.19 3.83 1.19 0.74 0.88 0.94 1.29 1.12 0.95 2.89 18.02
f1 (ppm)

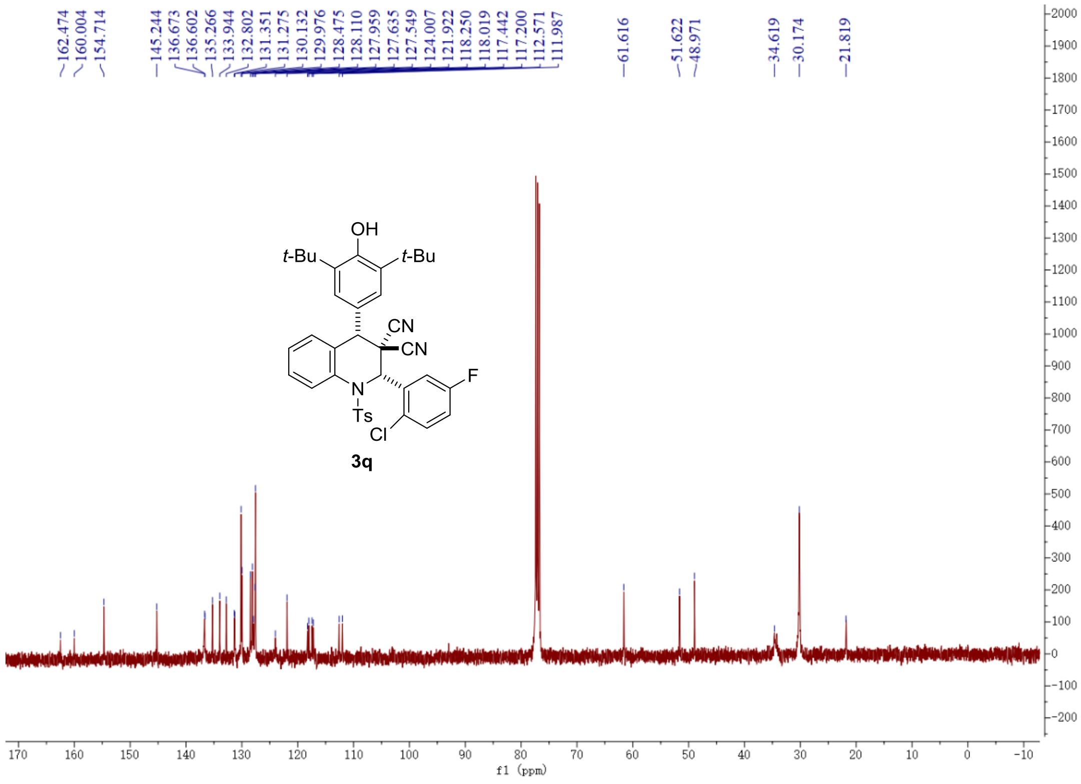
162.474 160.004 154.714
145.244 136.673 136.602 135.266 133.944 132.802 131.351 131.275 130.132 129.976 128.475 128.110 127.959 127.635 127.549 124.007 121.922 118.250 118.019 117.442 117.200 112.571 111.987
61.616
51.622 48.971
34.619 30.174
21.819
OH
t-Bu
t-Bu
CN
CN
F
N
Ts
Cl
3q
f1 (ppm)

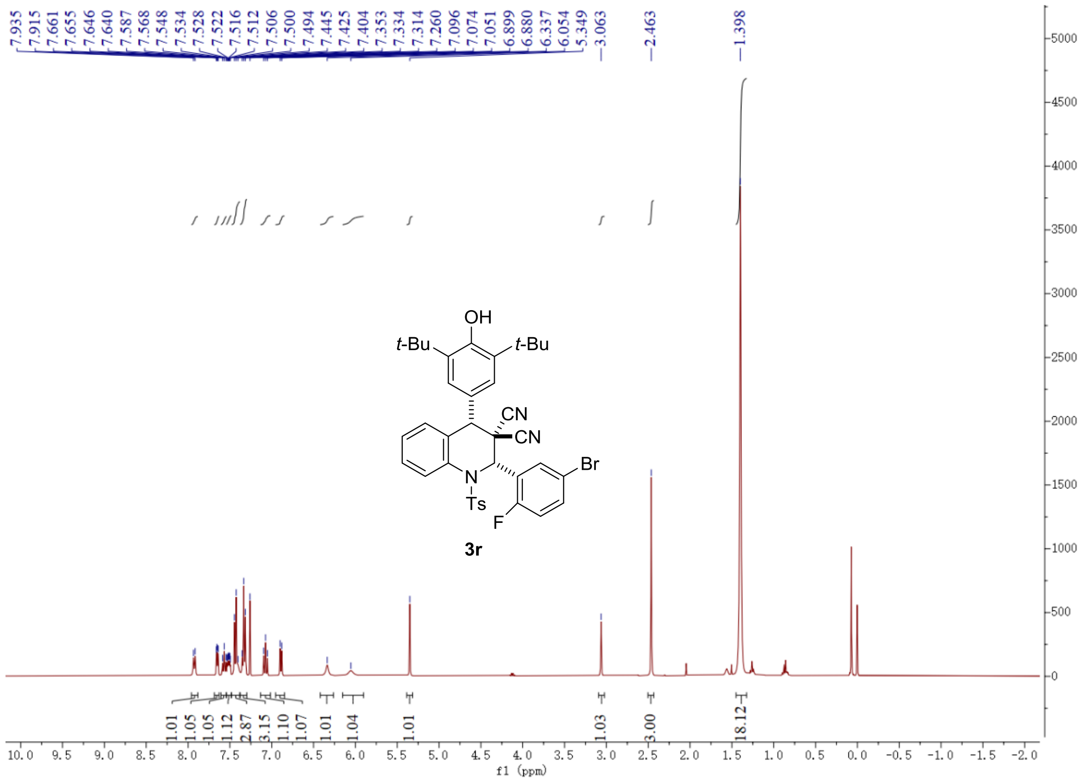
7.935
7.915
7.661
7.655
7.646
7.640
7.587
7.568
7.548
7.534
7.528
7.522
7.516
7.512
7.506
7.500
7.494
7.445
7.425
7.404
7.353
7.334
7.314
7.260
7.096
7.074
7.051
6.899
6.880
6.337
6.054
5.349
3.063
2.463
1.398
OH
t-Bu
t-Bu
CN
CN
N
Ts
Br
F
3r
1.01
1.05
1.05
1.12
2.87
3.15
1.10
1.07
1.01
1.04
1.01
1.03
3.00
18.12
f1 (ppm)

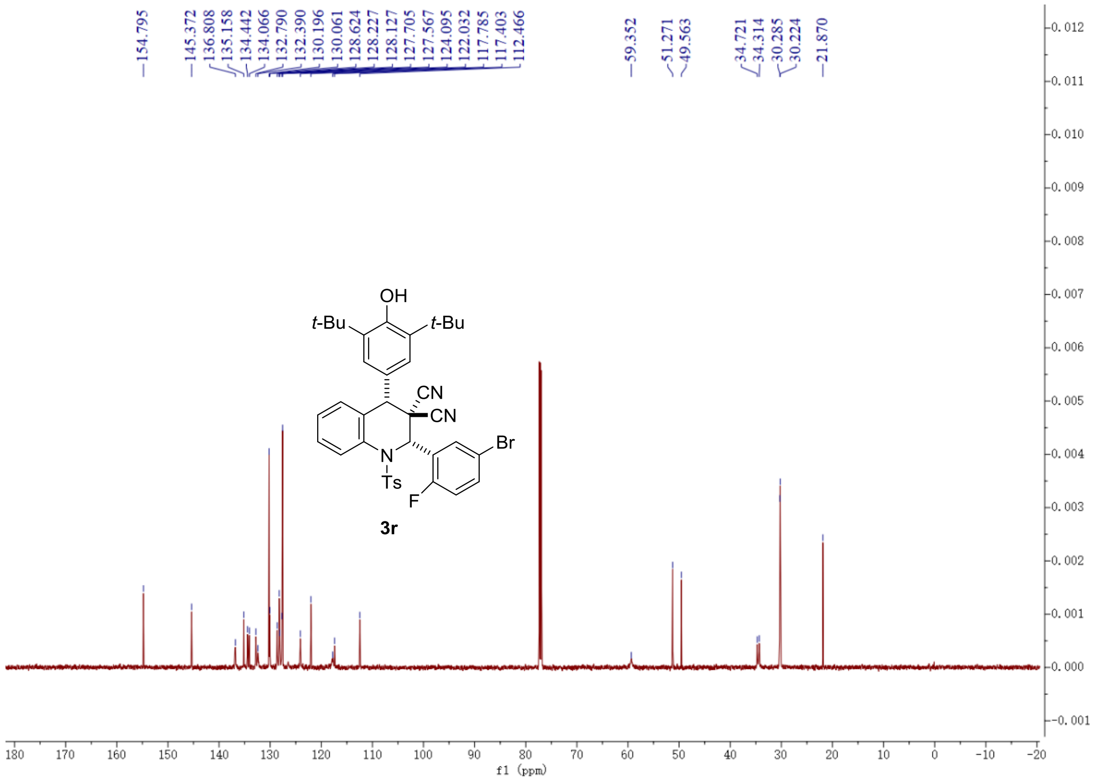
154.795
145.372
136.808
135.158
134.442
134.066
132.790
132.390
130.196
130.061
128.624
128.227
128.127
127.705
127.567
124.095
122.032
117.785
117.403
112.466
59.352
51.271
49.563
34.721
34.314
30.285
30.224
21.870
OH
t-Bu
t-Bu
CN
CN
N
Ts
Br
F
3r
f1 (ppm)

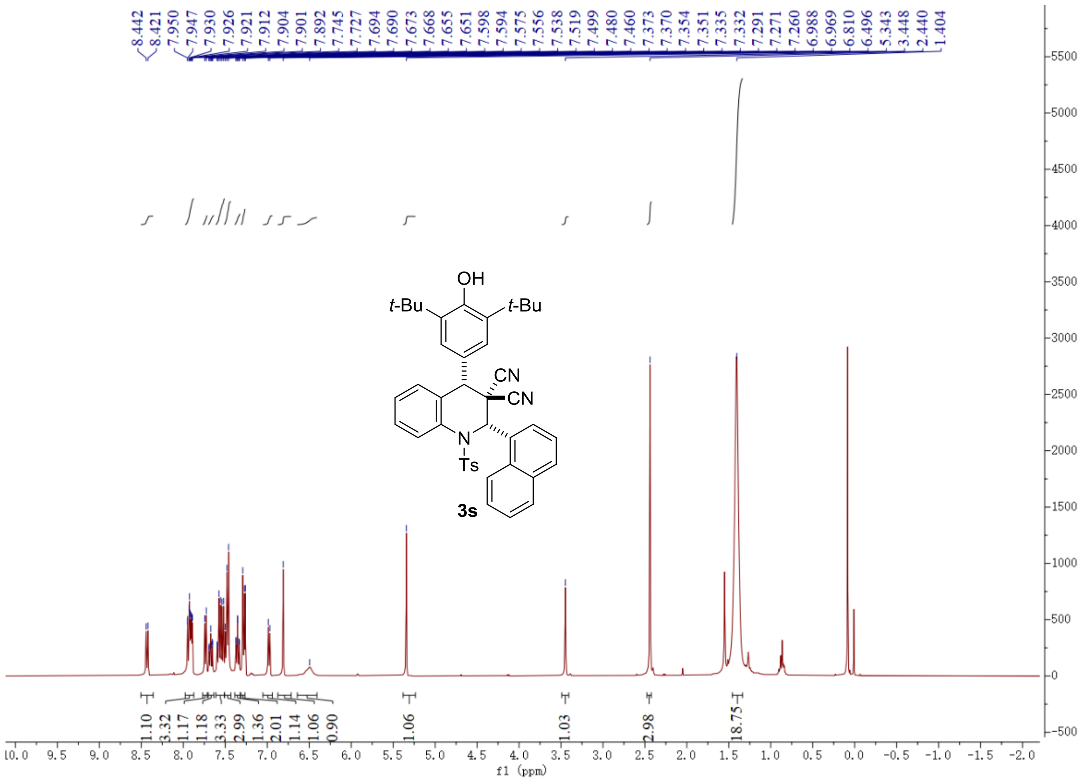

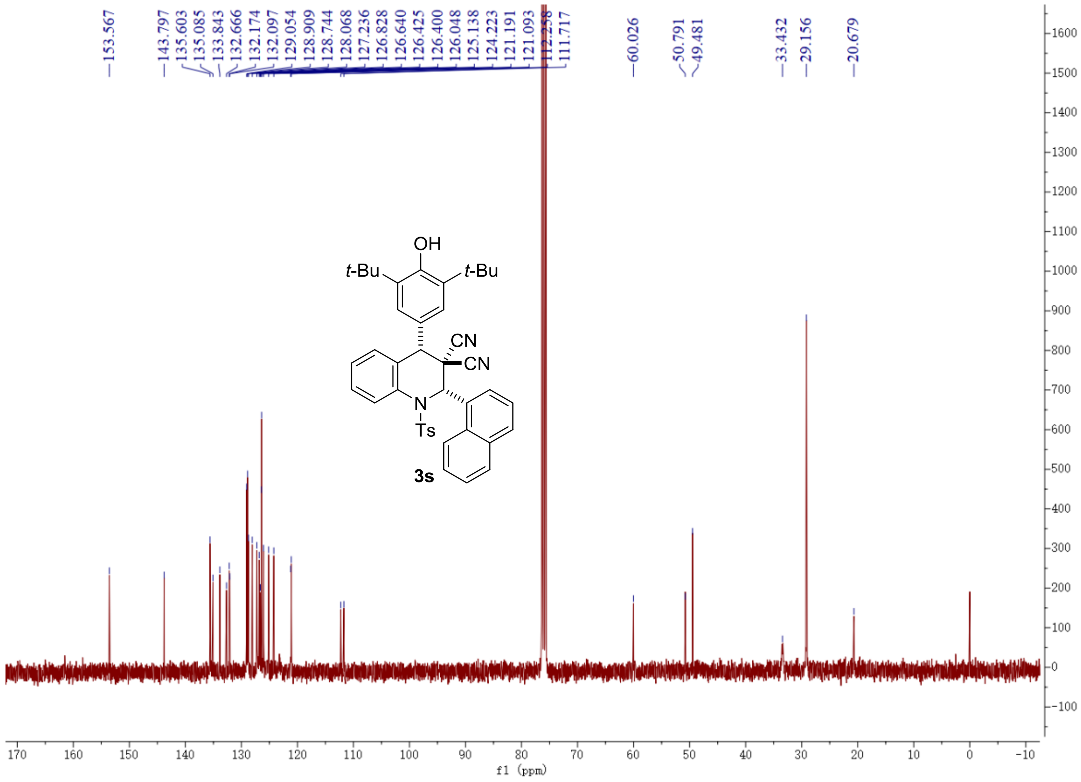

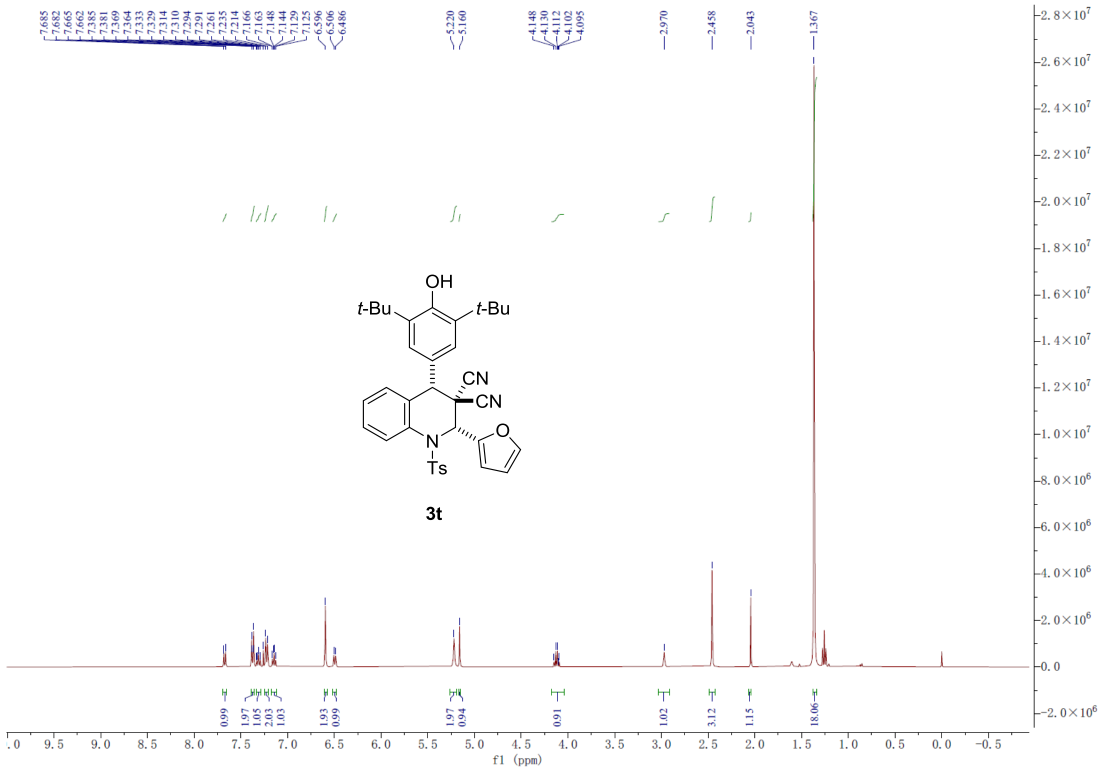
7.685
7.682
7.665
7.662
7.385
7.381
7.369
7.364
7.333
7.329
7.314
7.310
7.294
7.291
7.261
7.235
7.214
7.166
7.163
7.148
7.144
7.129
7.125
6.596
6.506
6.486
5.220
5.160
4.148
4.130
4.112
4.102
4.095
2.970
2.458
2.043
1.367
OH
t-Bu
t-Bu
CN
CN
N
Ts
O
3t
0.99
1.97
1.05
2.03
1.03
1.93
0.99
1.97
0.94
0.91
1.02
3.12
1.15
18.06
f1 (ppm)

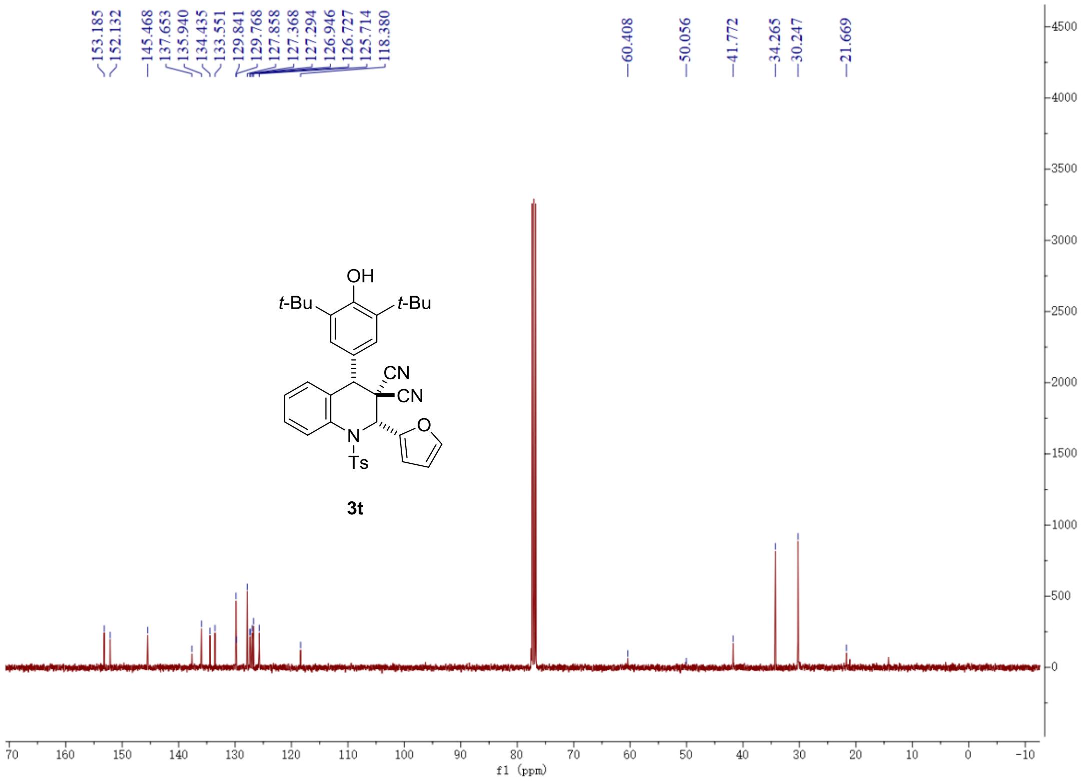
153.185
152.132
145.468
137.653
135.940
134.435
133.551
129.841
129.768
127.858
127.368
127.294
126.946
126.727
125.714
118.380
60.408
50.056
41.772
34.265
30.247
21.669
OH
t-Bu
t-Bu
CN
CN
N
Ts
O
3t
f1 (ppm)

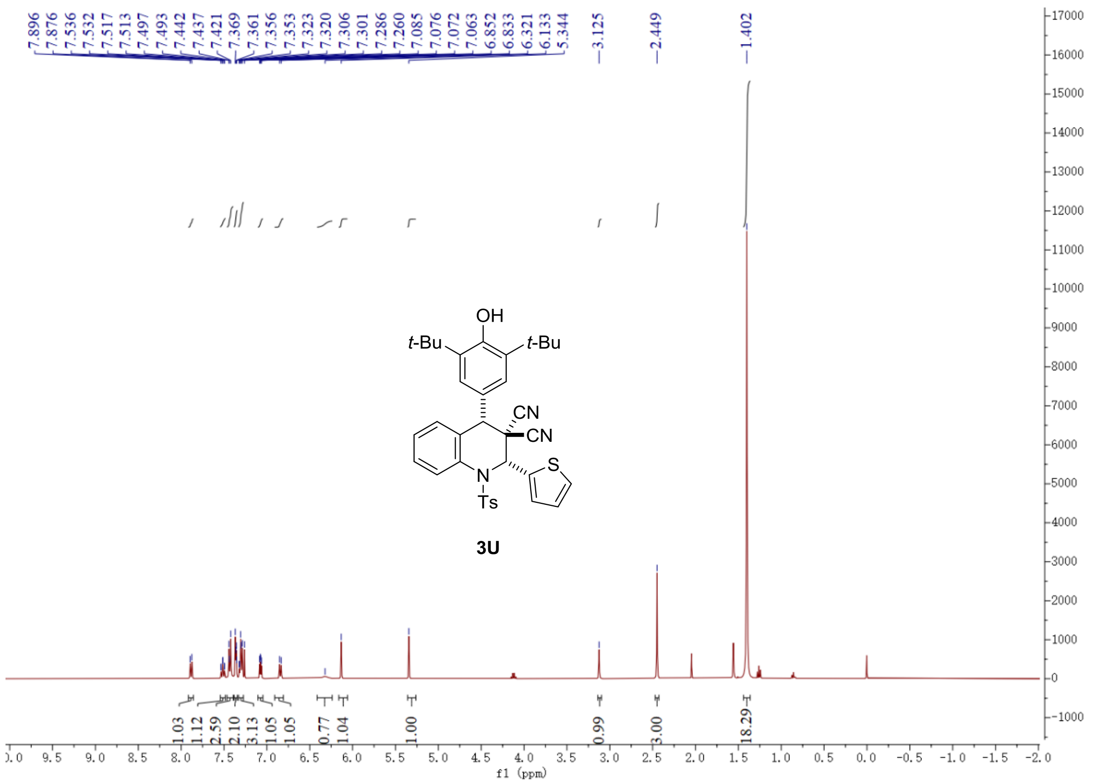

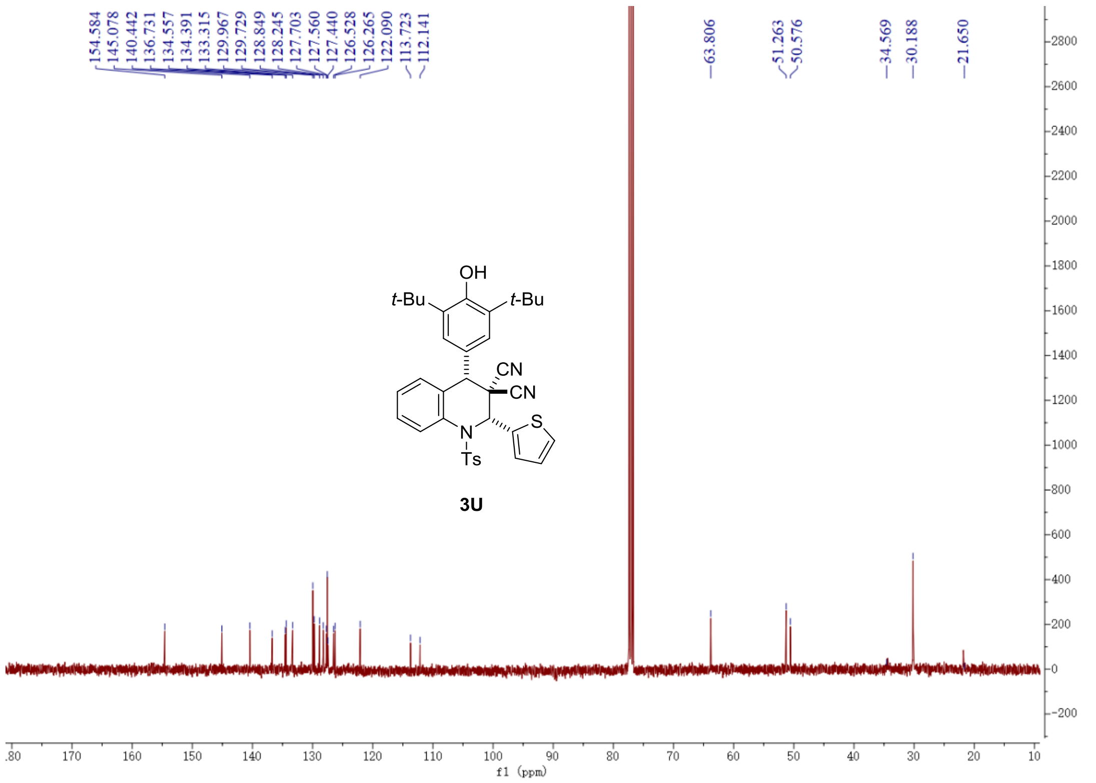

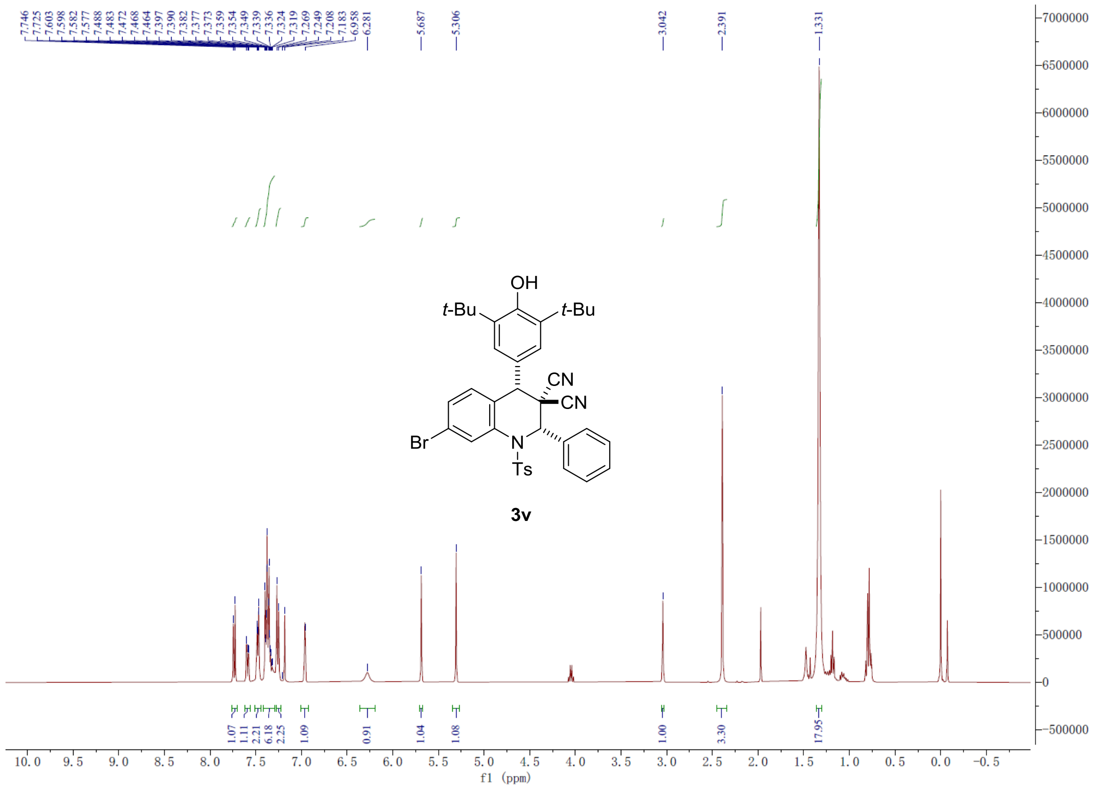
7.746
7.725
7.603
7.598
7.582
7.577
7.488
7.483
7.468
7.464
7.397
7.390
7.382
7.377
7.373
7.359
7.354
7.349
7.339
7.336
7.324
7.319
7.269
7.249
7.208
7.183
6.958
6.281
5.687
5.306
3.042
2.391
1.331
OH
t-Bu
t-Bu
CN
CN
Br
N
Ts
3v
1.07
1.11
2.21
6.18
2.25
1.09
0.91
1.04
1.08
1.00
3.30
17.95
f1 (ppm)

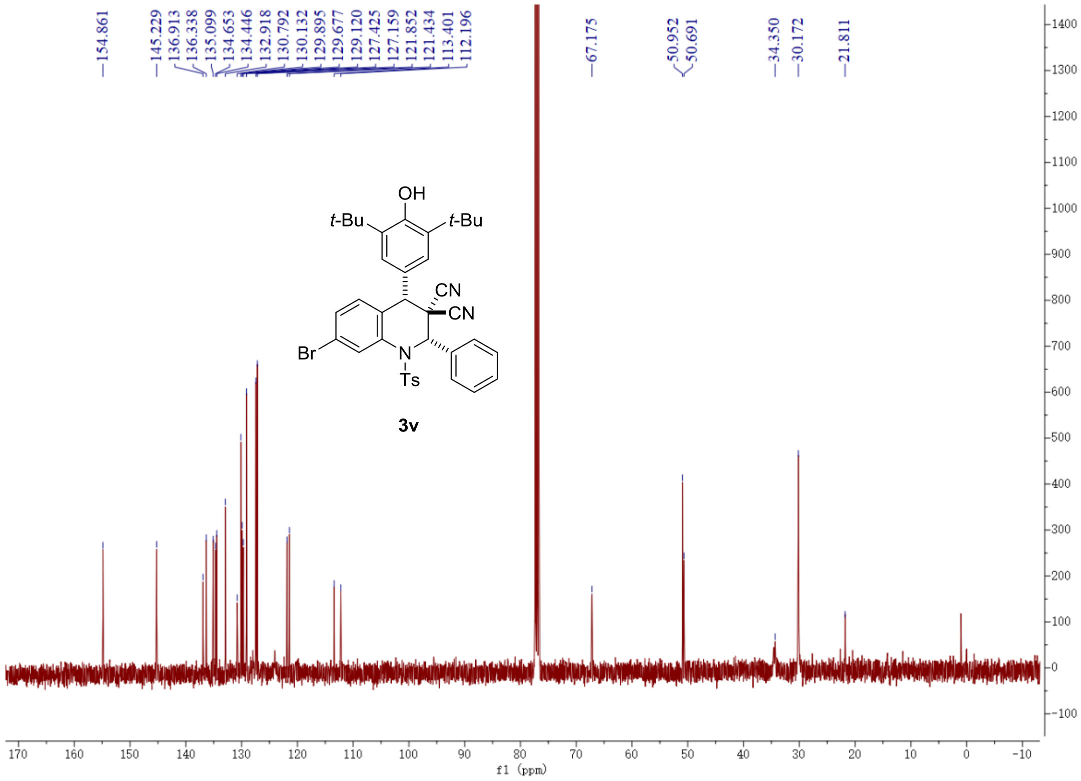
154.861
145.229
136.913
136.338
135.099
134.653
134.446
132.918
130.792
130.132
129.895
129.677
129.120
127.425
127.159
121.852
121.434
113.401
112.196
67.175
50.952
50.691
34.350
30.172
21.811
OH
t-Bu
t-Bu
CN
CN
Br
N
Ts
3v
f1 (ppm)

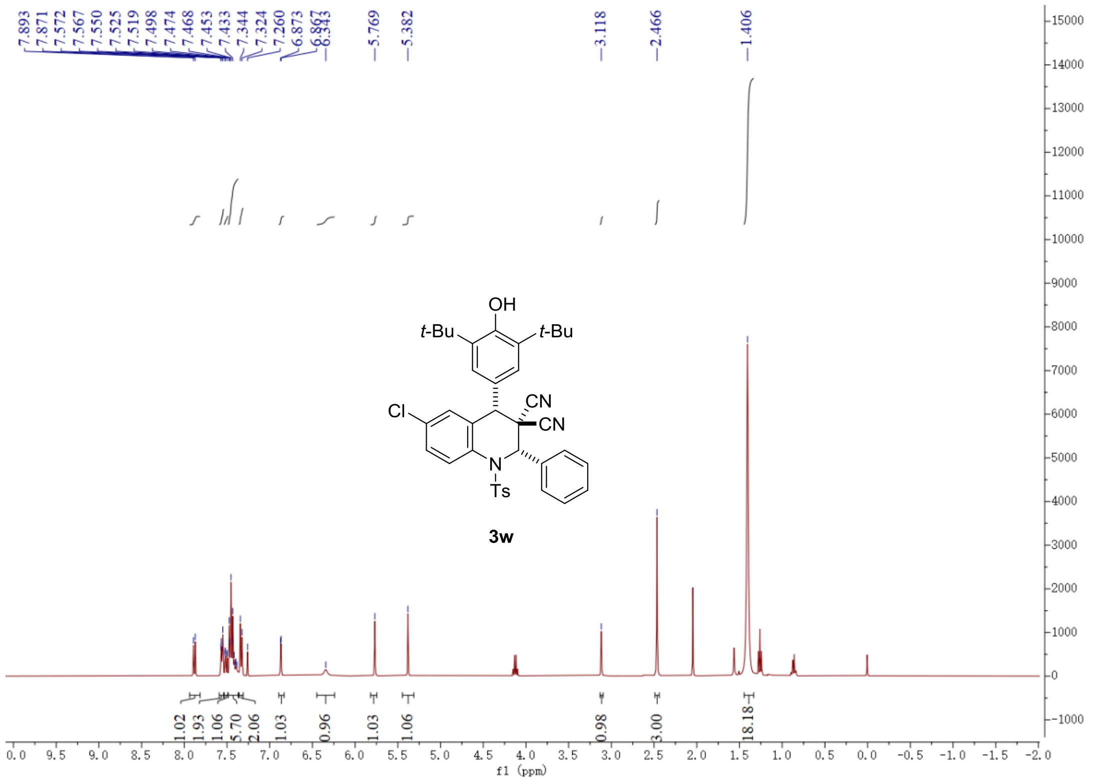
7.893
7.871
7.572
7.567
7.550
7.525
7.519
7.498
7.474
7.468
7.453
7.433
7.344
7.324
7.260
6.873
6.343
5.769
5.382
3.118
2.466
1.406
OH
t-Bu
t-Bu
Cl
CN
CN
N
Ts
3w
1.02
1.93
1.06
5.70
2.06
1.03
0.96
1.03
1.06
0.98
3.00
18.18
f1 (ppm)

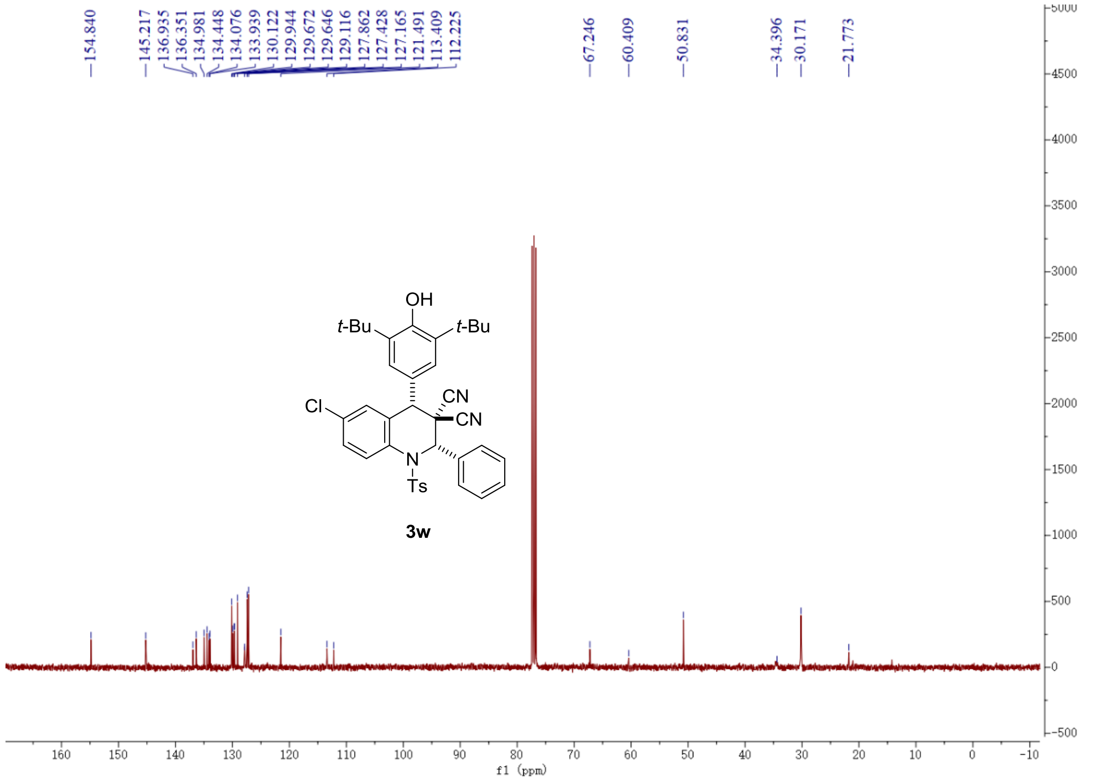
154.840
145.217
136.935
136.351
134.981
134.448
134.076
133.939
130.122
129.944
129.672
129.646
129.116
127.862
127.428
127.165
121.491
113.409
112.225
67.246
60.409
50.831
34.396
30.171
21.773
OH
t-Bu
t-Bu
Cl
CN
CN
N
Ts
3w
f1 (ppm)

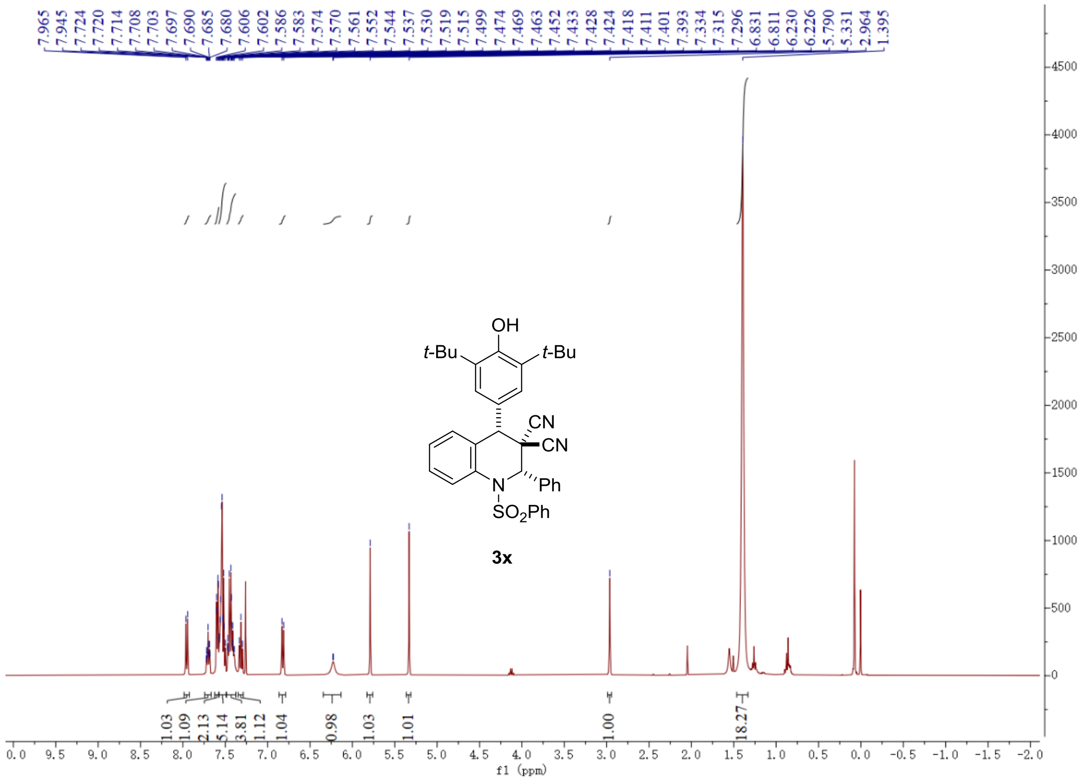
OH
t-Bu
t-Bu
CN
CN
N
Ph
SO2Ph
3x
f1 (ppm)

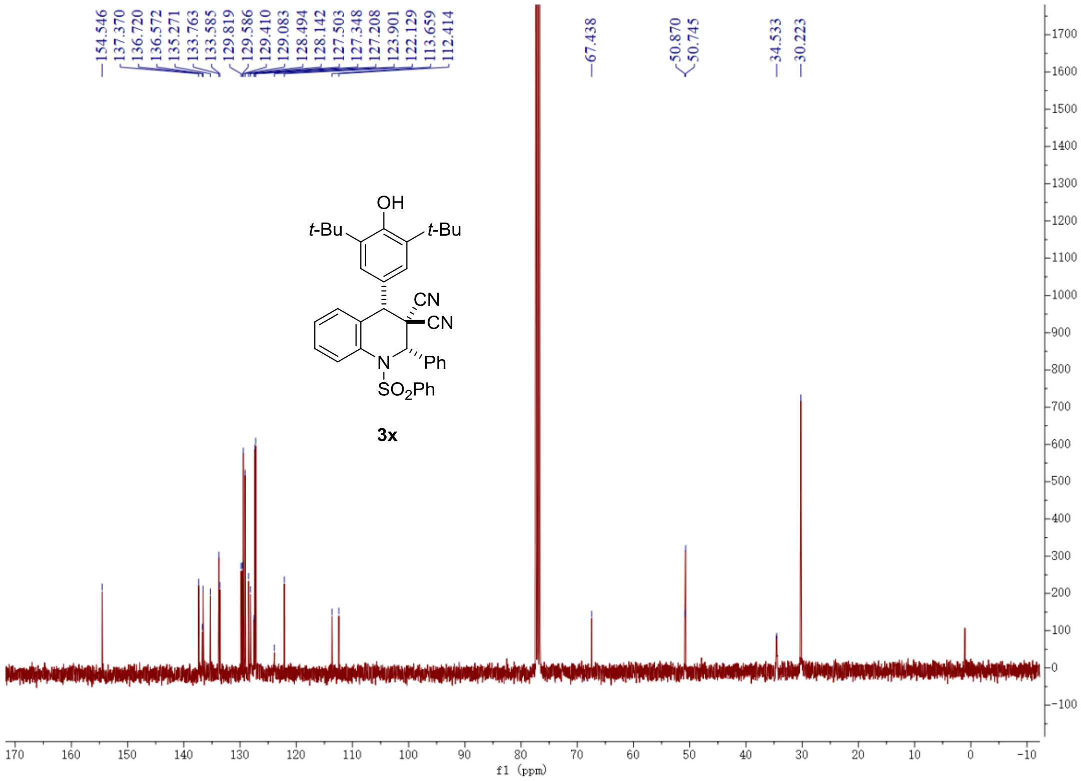
OH
t-Bu
t-Bu
CN
CN
N
Ph
SO2Ph
3x
f1 (ppm)

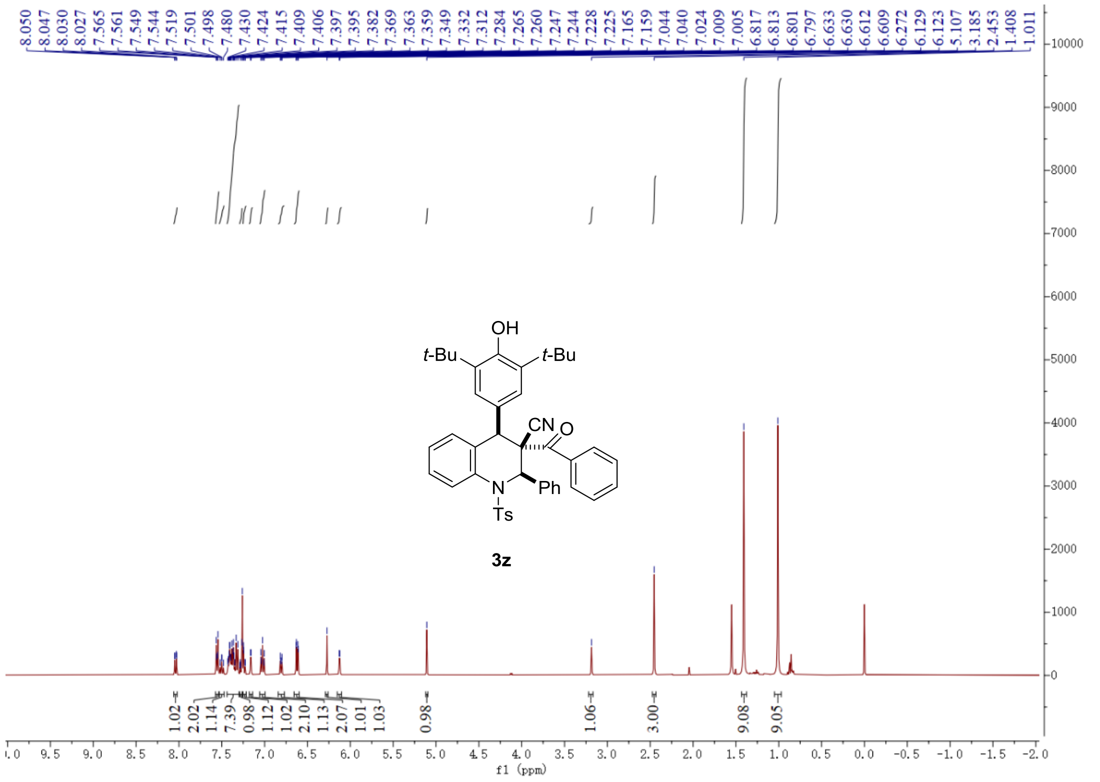

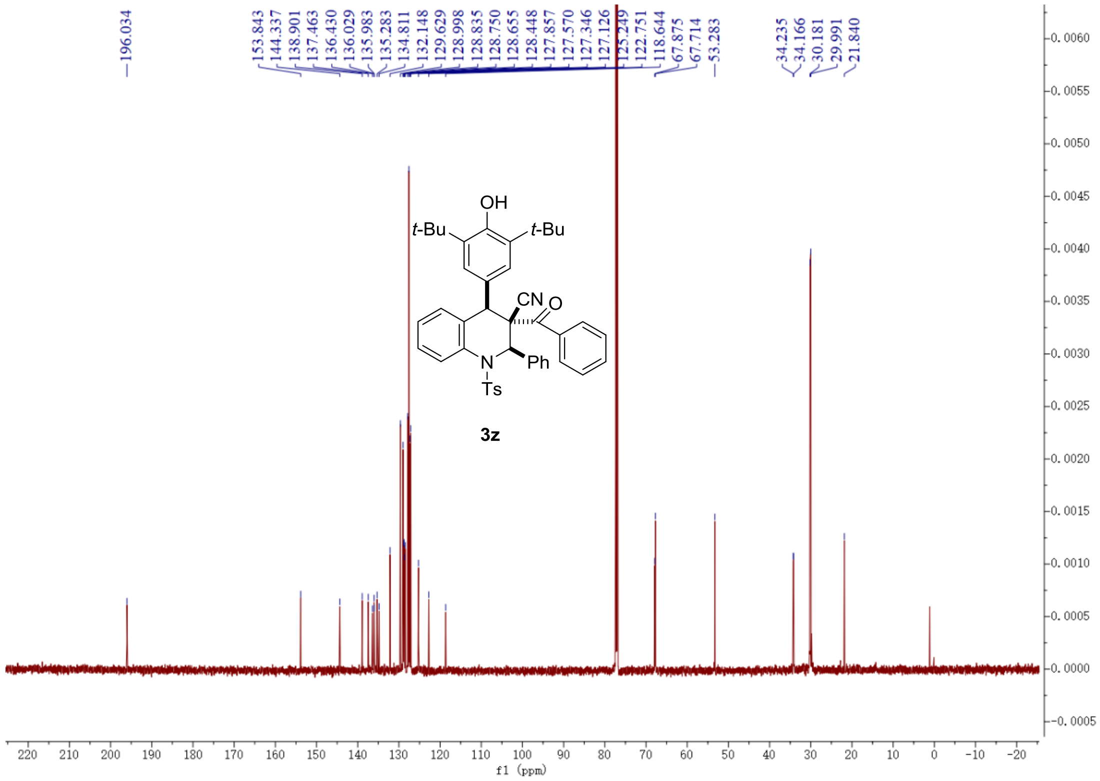

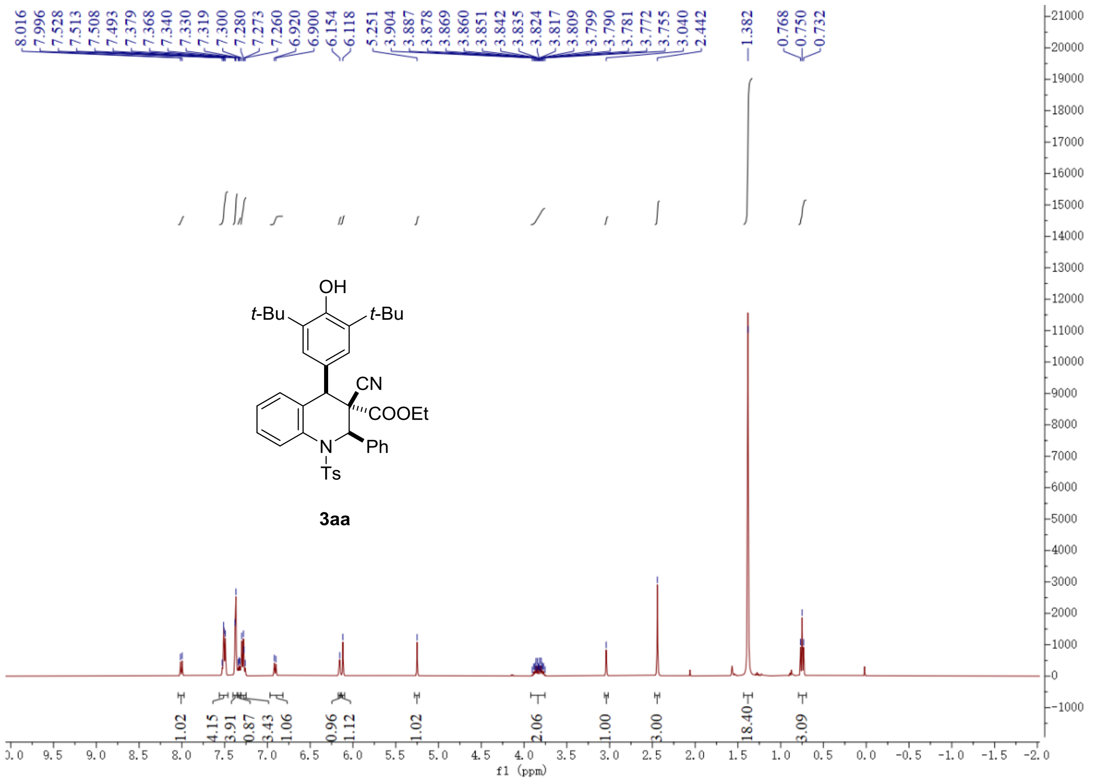
8.016
7.996
7.528
7.513
7.508
7.493
7.379
7.368
7.340
7.330
7.319
7.300
7.280
7.273
7.260
6.920
6.900
6.154
6.118
5.251
3.904
3.887
3.878
3.869
3.860
3.851
3.842
3.835
3.824
3.817
3.809
3.799
3.790
3.781
3.772
3.755
3.040
2.442
1.382
0.768
0.750
0.732
OH
t-Bu
t-Bu
CN
COOEt
N
Ph
Ts
3aa
1.02
4.15
3.91
0.87
3.43
1.06
0.96
1.12
1.02
2.06
1.00
3.00
18.40
3.09
f1 (ppm)

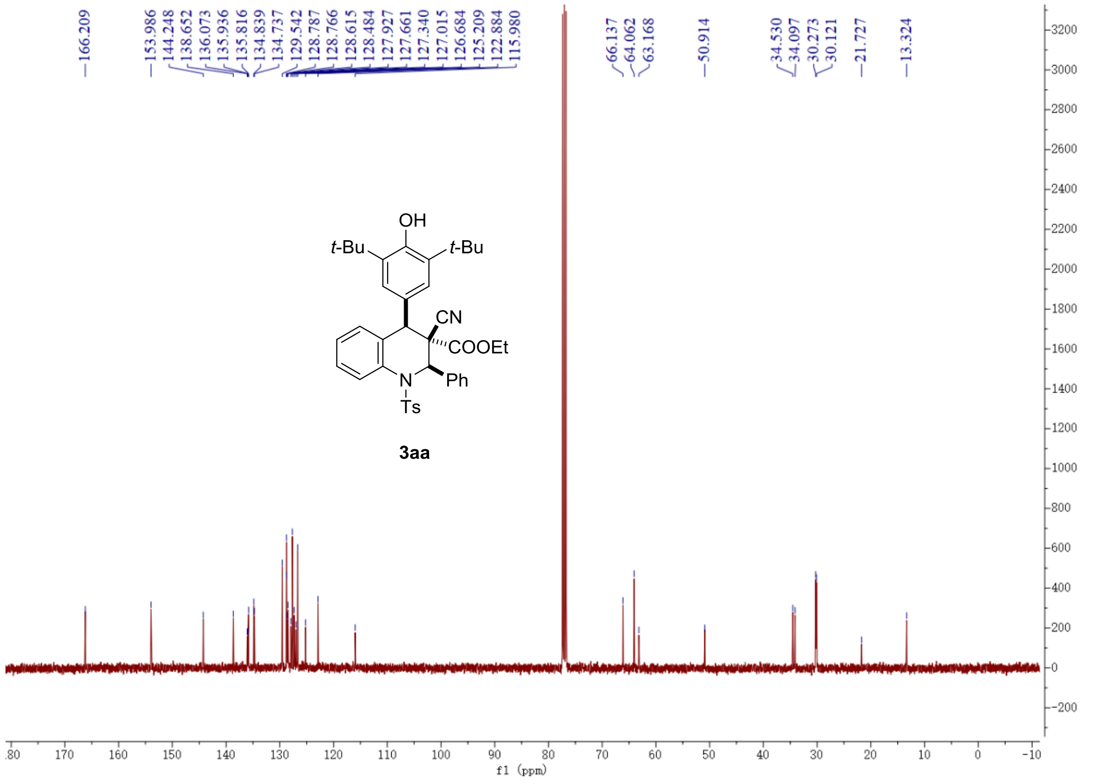
166.209
153.986
144.248
138.652
136.073
135.936
135.816
134.839
134.737
129.542
128.787
128.766
128.615
128.484
127.927
127.661
127.340
127.015
126.684
125.209
122.884
115.980
66.137
64.062
63.168
50.914
34.530
34.097
30.273
30.121
21.727
13.324
OH
t-Bu
t-Bu
CN
COOEt
N
Ph
Ts
3aa
f1 (ppm)

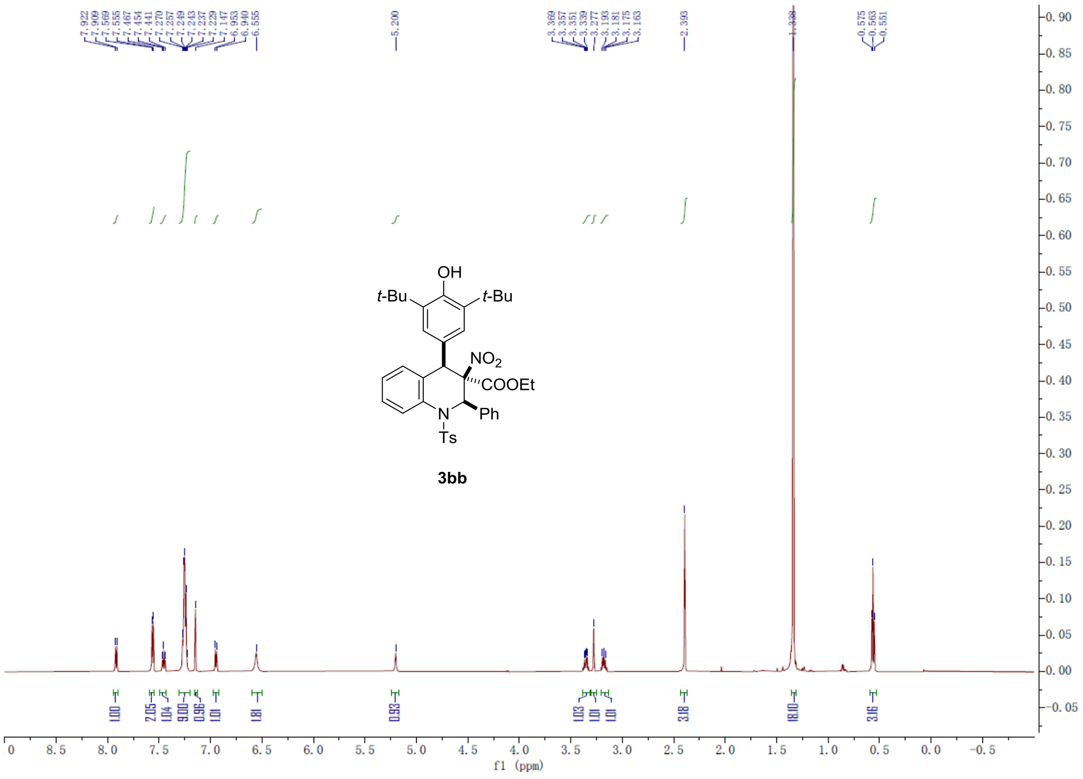

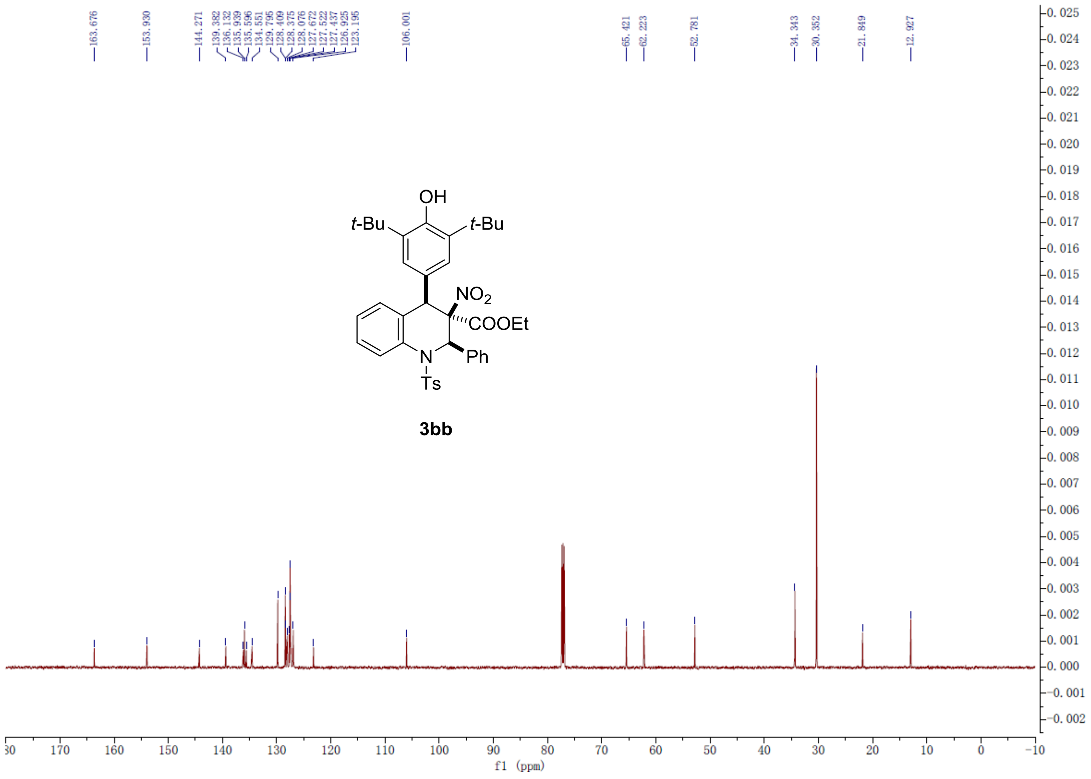

NMR spectra of products 5, 7 and 8

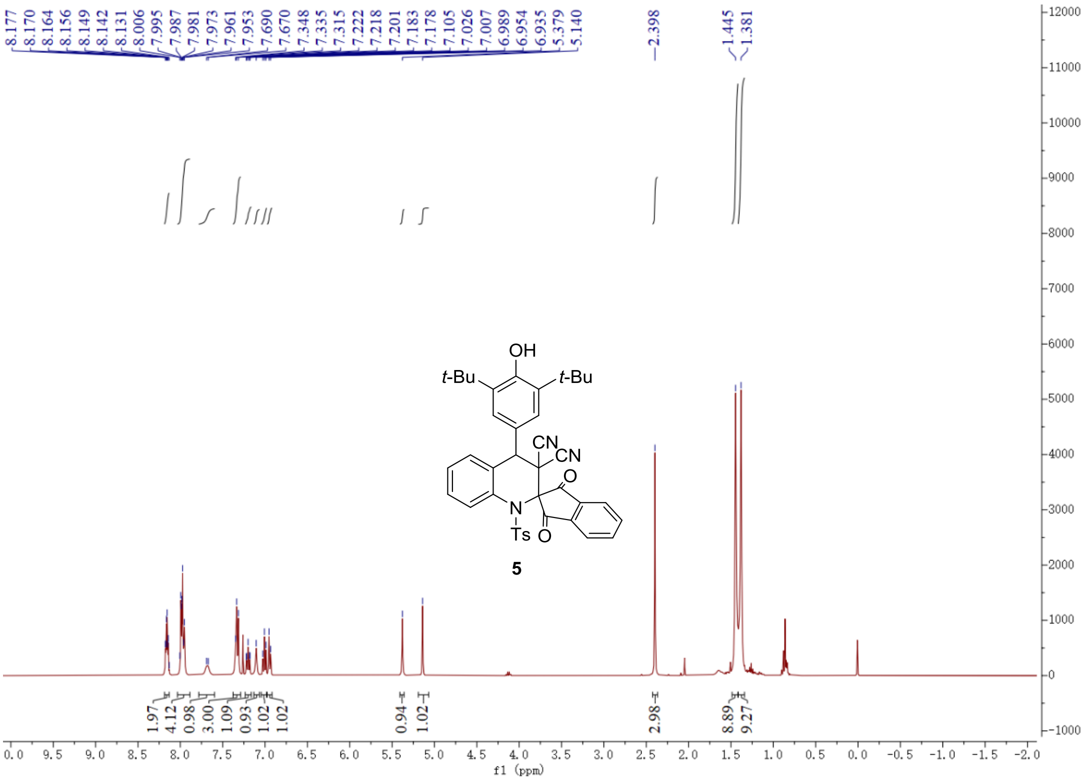

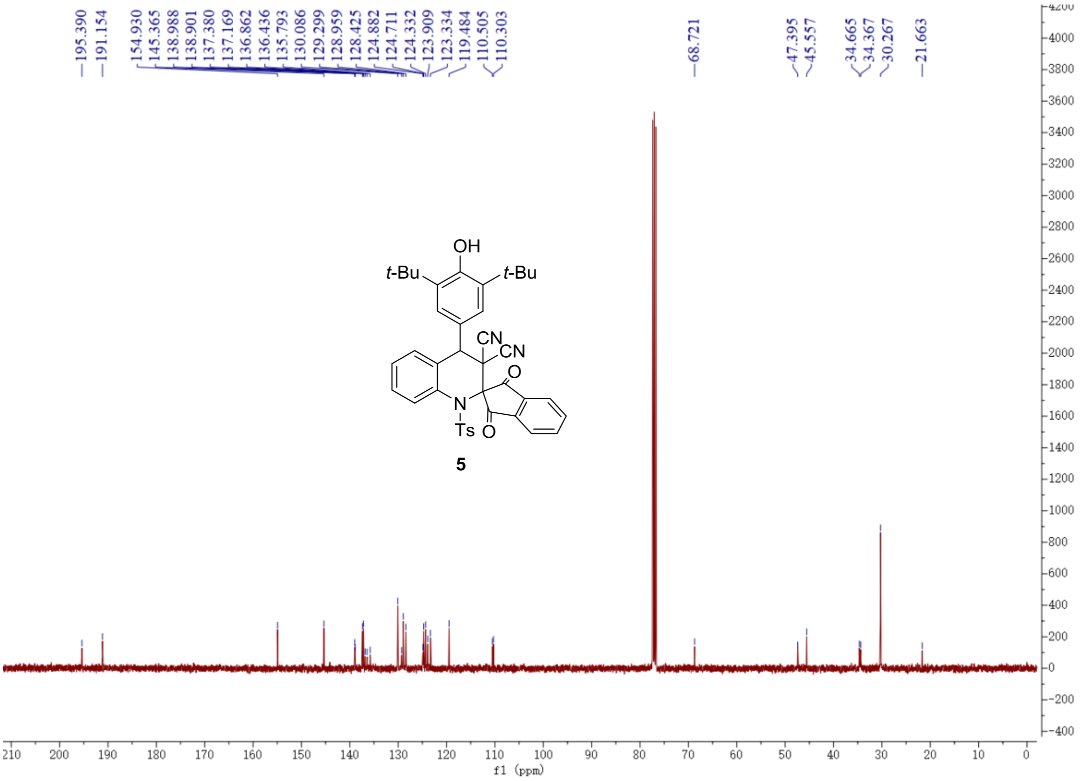

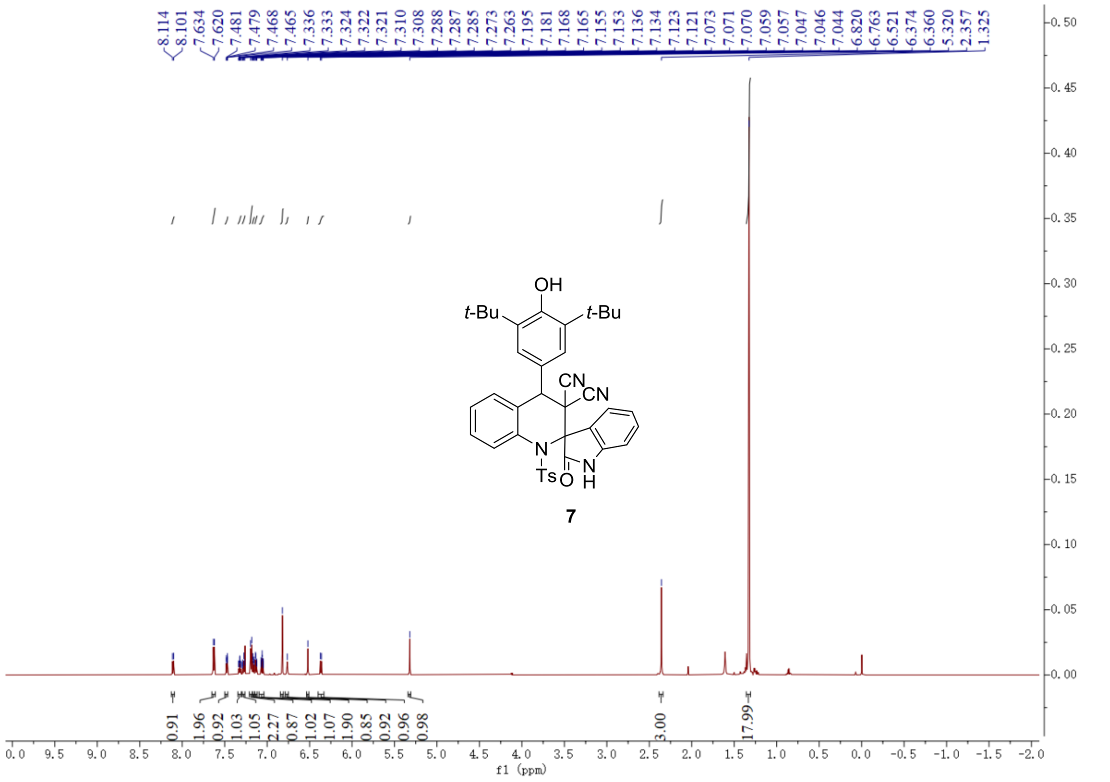
8.114
8.101
7.634
7.620
7.481
7.479
7.468
7.465
7.336
7.333
7.324
7.322
7.321
7.310
7.308
7.288
7.287
7.285
7.273
7.263
7.195
7.181
7.168
7.165
7.155
7.153
7.136
7.134
7.123
7.121
7.073
7.071
7.070
7.059
7.057
7.047
7.046
7.044
6.820
6.763
6.521
6.374
6.360
5.320
2.357
1.325
OH
t-Bu
t-Bu
CN
CN
N
Ts
O
N
H
7
0.91
1.96
0.92
1.03
1.05
2.27
0.87
1.02
1.07
1.90
0.85
0.92
0.96
0.98
3.00
17.99
f1 (ppm)

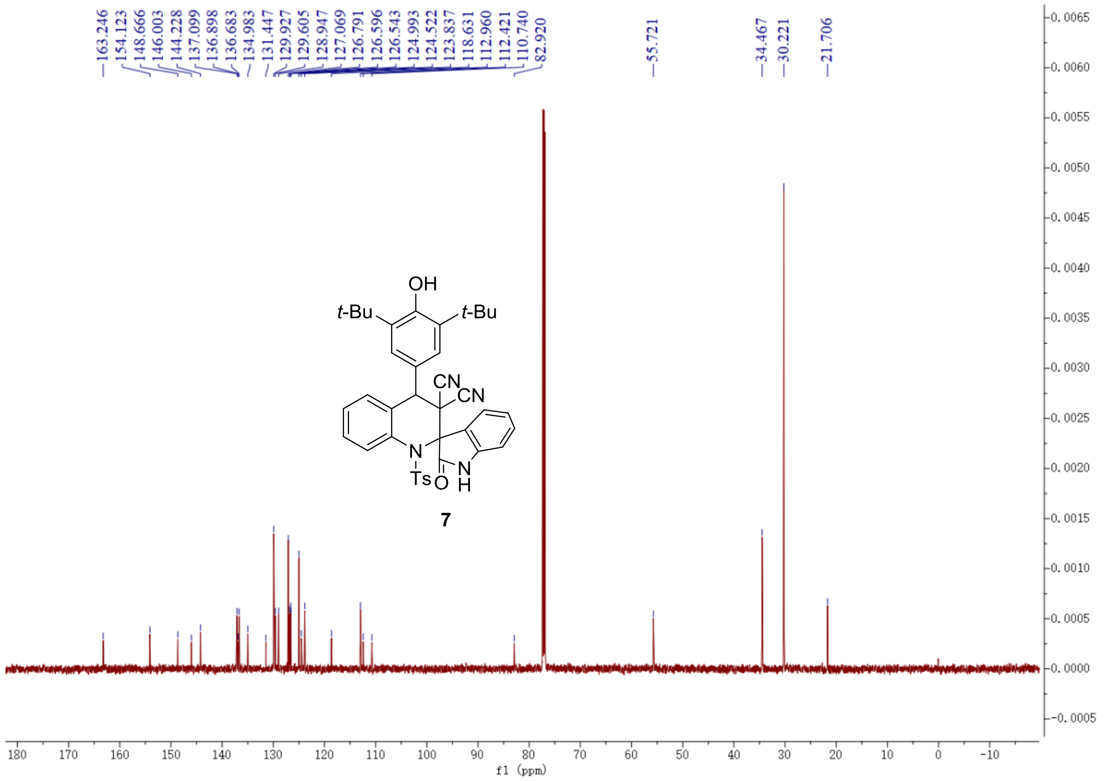
163.246
154.123
148.666
146.003
144.228
137.099
136.898
136.683
134.983
131.447
129.927
129.605
128.947
127.069
126.791
126.596
126.543
124.993
124.522
123.837
118.631
112.960
112.421
110.740
82.920
55.721
34.467
30.221
21.706
OH
t-Bu
t-Bu
CN
CN
N
Ts
O
N
H
7
f1 (ppm)

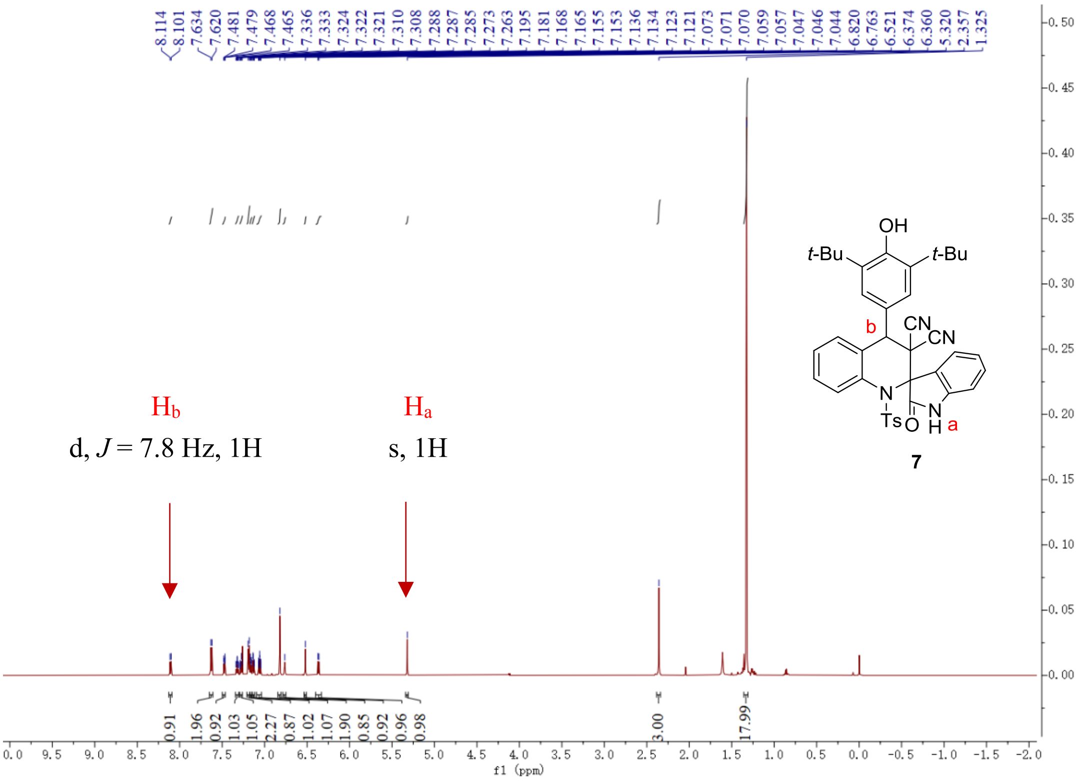
8.114
8.101
7.634
7.620
7.481
7.479
7.468
7.465
7.336
7.333
7.324
7.322
7.321
7.310
7.308
7.288
7.287
7.285
7.273
7.263
7.195
7.181
7.168
7.165
7.155
7.153
7.136
7.134
7.123
7.121
7.073
7.071
7.070
7.059
7.057
7.047
7.046
7.044
6.820
6.763
6.521
6.374
6.360
5.320
2.357
1.325
Hb
d, J = 7.8 Hz, 1H
Ha
s, 1H
OH
t-Bu
t-Bu
b
CN
CN
N
Ts
O
N
H
a
7
0.91
1.96
0.92
1.03
1.05
2.27
0.87
1.02
1.07
1.90
0.85
0.92
0.96
0.98
3.00
17.99
f1 (ppm)

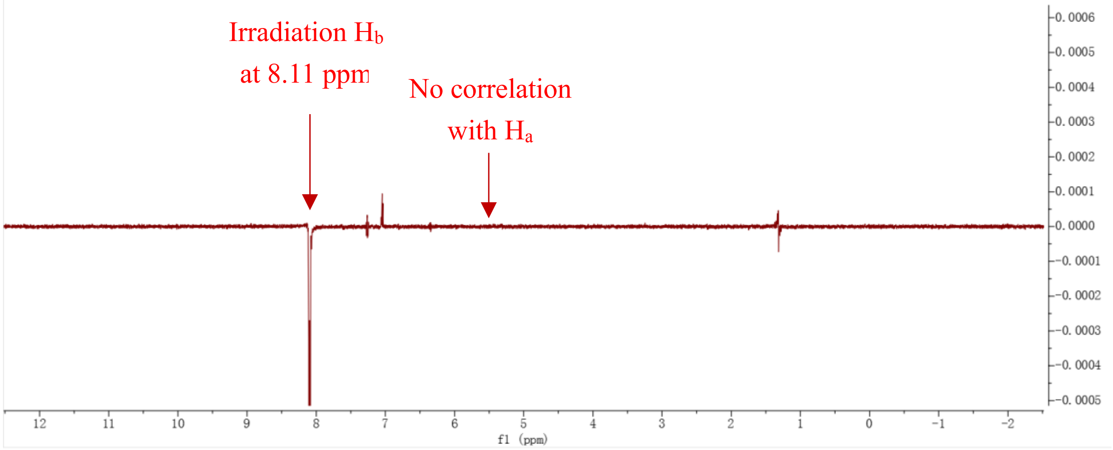
Irradiation Hb
at 8.11 ppm
No correlation
with Ha
f1 (ppm)

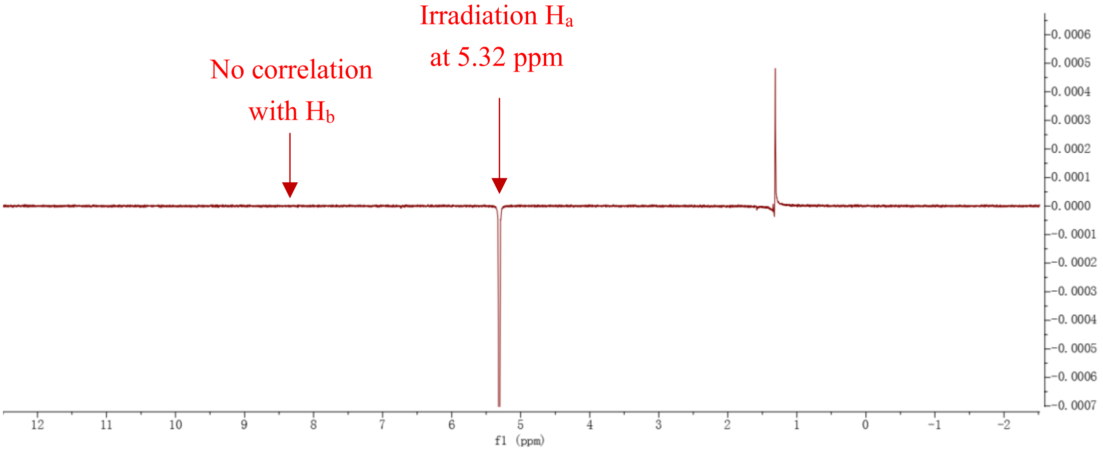
Irradiation Ha
at 5.32 ppm
No correlation
with Hb
f1 (ppm)

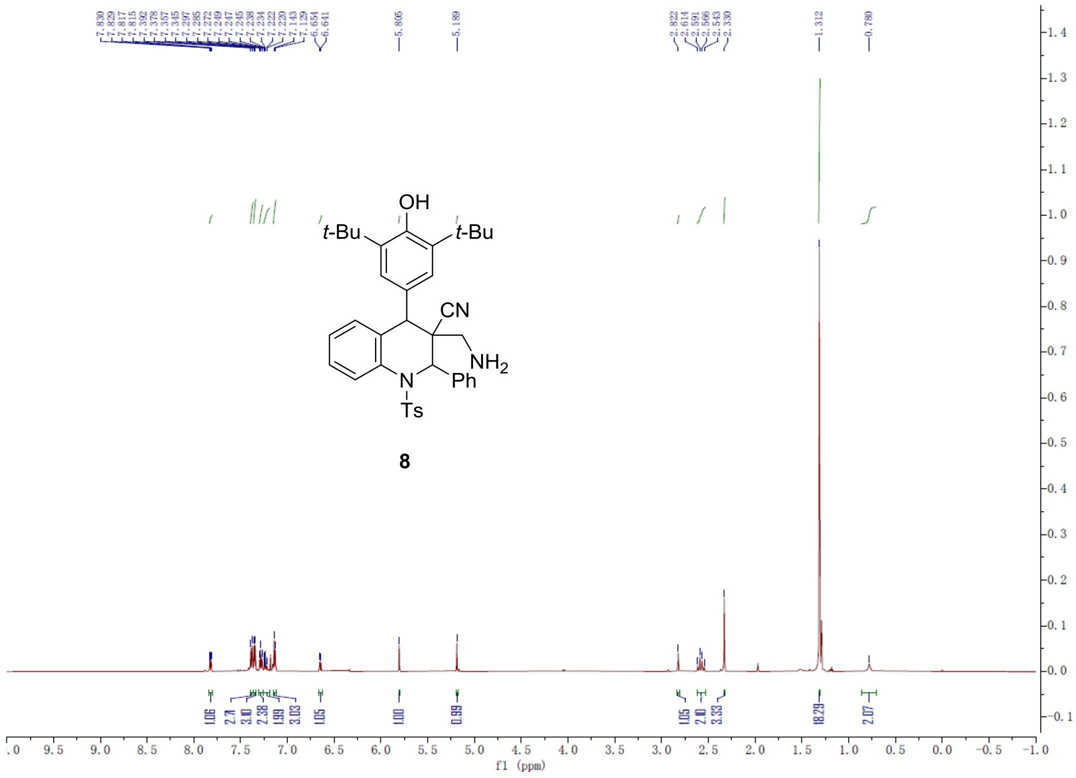

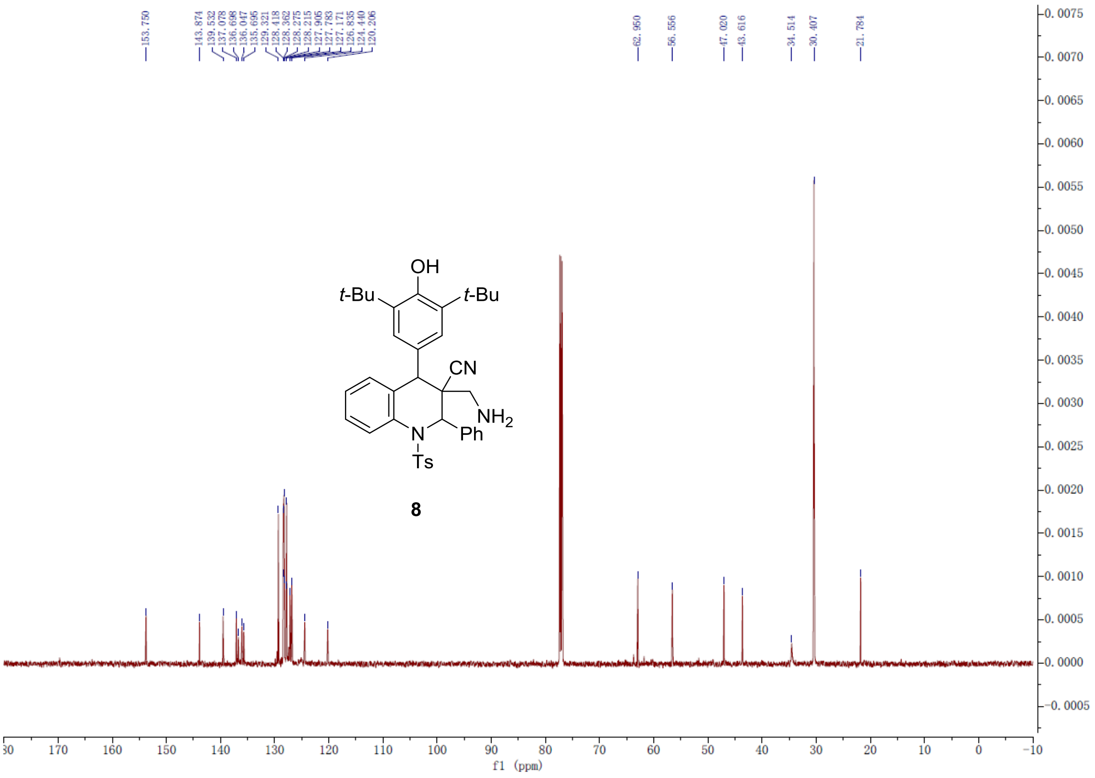

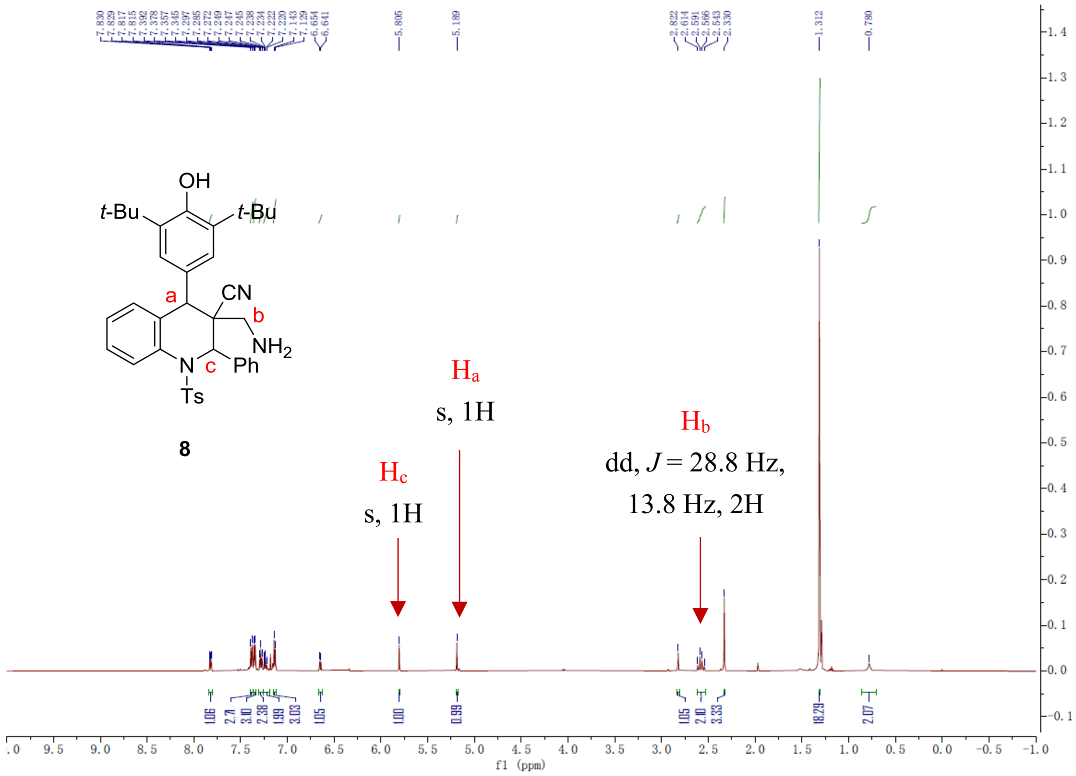
OH
t-Bu
t-Bu
a
CN
b
NH2
N
c
Ph
Ts
8
Ha
s, 1H
Hc
s, 1H
Hb
dd, J = 28.8 Hz, 13.8 Hz, 2H
f1 (ppm)

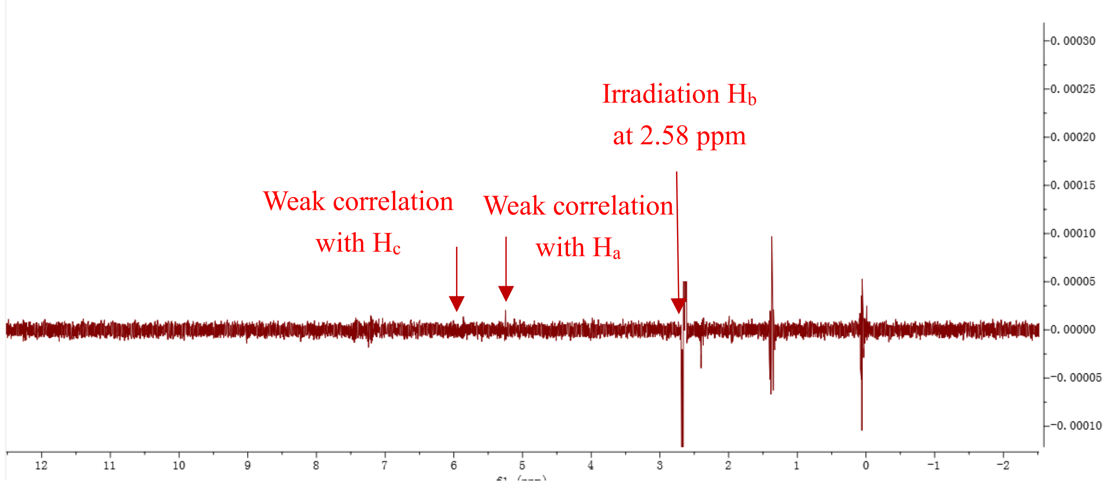
Irradiation Hb at 2.58 ppm
Weak correlation with Hc
Weak correlation with Ha

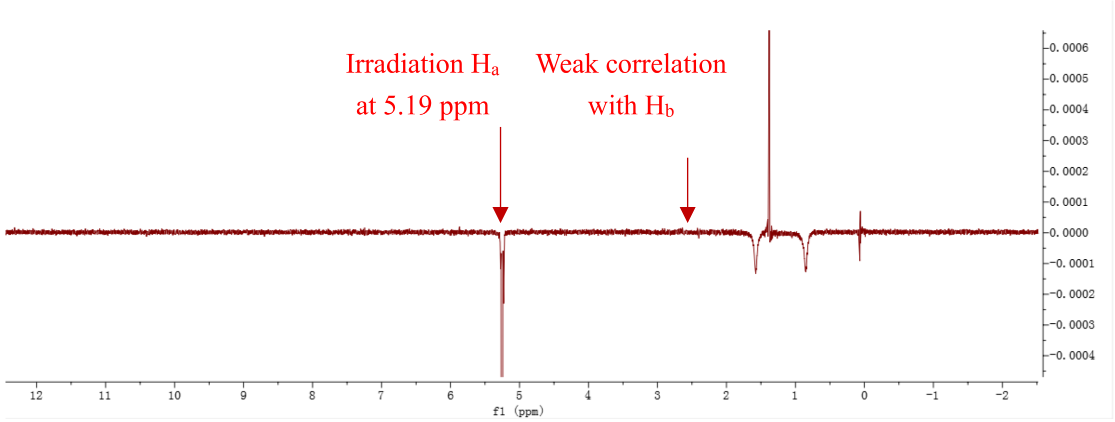
Irradiation Ha at 5.19 ppm
Weak correlation with Hb
f1 (ppm)

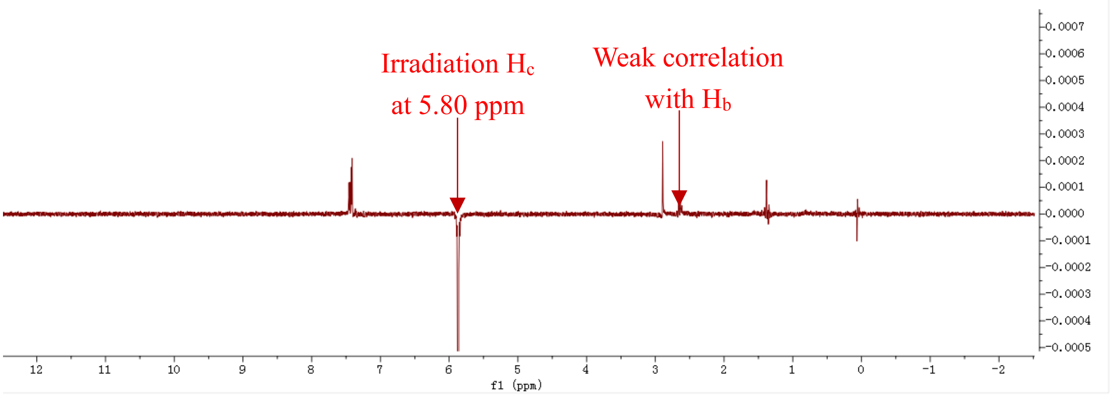
Irradiation Hc
at 5.80 ppm
Weak correlation
with Hb
f1 (ppm)

Crystal data of prodcut 3a and 3z

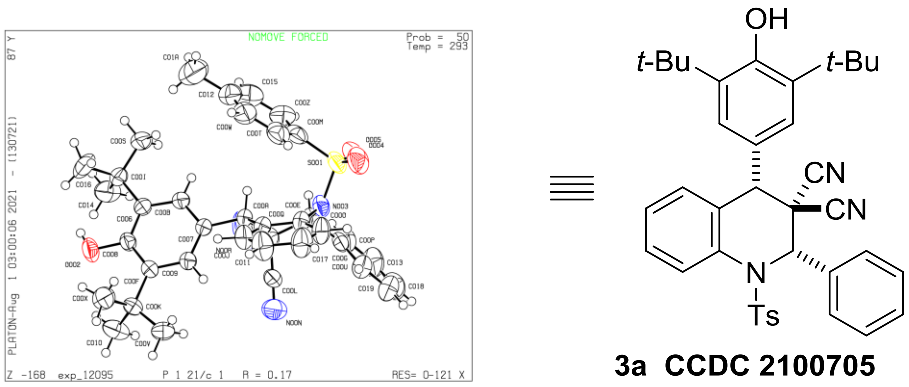

3a CCDC 2100705

Table 1 Crystal data and structure refinement for compound 3a.

Identification code	compound 3a
Empirical formula	C10HN11O2S
Formula weight	339.28
Temperature/K	293(2)
Crystal system	monoclinic
Space group	P21/c
a/Å	11.0596(10)
b/Å	34.5548(16)
c/Å	11.2811(10)
α/°	90
β/°	119.369(12)
γ/°	90
Volume/Å3	3757.1(6)
Z	1
ρcalcg/cm3	0.150
μ/mm-1	0.223
F(000)	170.0
Crystal size/mm3	? × ? × ?
Radiation	Cu Kα (λ = 1.54184)
2Θ range for data collection/°	9.176 to 143.618
Index ranges	-13 ≤ h ≤ 13, -29 ≤ k ≤ 42, -13 ≤ l ≤ 13
Reflections collected	24487
Independent reflections	7193 [Rint = 0.0628, Rsigma = 0.0630]
Data/restraints/parameters	7193/0/414
Goodness-of-fit on F2	1.763
Final R indexes [I>=2σ (I)]	R1 = 0.1735, wR2 = 0.4432
Final R indexes [all data]	R1 = 0.2139, wR2 = 0.4989
Largest diff. peak/hole / e Å-3	3.62/-0.79

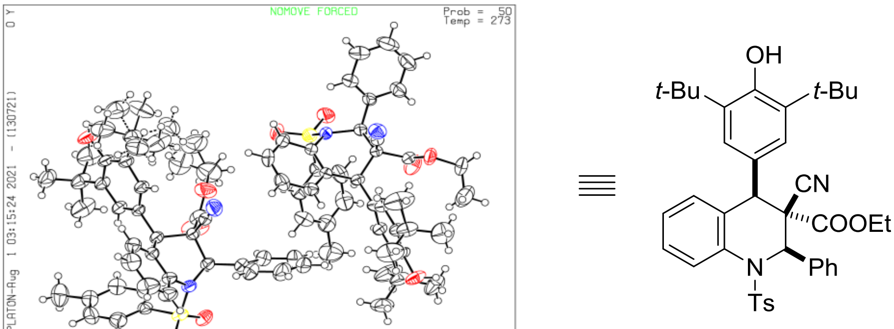

3z CCDC 2100706

Table 2 Crystal data and structure refinement for compound 3z.

Identification code	compound 3z
Empirical formula	$C_{40}H_{44}N_2O_5S$
Formula weight	664.83
Temperature/K	273.15
Crystal system	monoclinic
Space group	$P2_1/n$
a/Å	21.6637(17)
b/Å	16.6690(13)
c/Å	21.9890(18)
α/°	90
β/°	112.2210(10)
γ/°	90
Volume/Å^3	7350.8(10)
Z	8
ρ_{calc}g/cm^3	1.201
μ/mm^{-1}	0.133
F(000)	2832.0
Crystal size/mm^3	? × ? × ?
Radiation	MoKα (λ = 0.71073)
2Θ range for data collection/°	4.69 to 50
Index ranges	$-23 \leq h \leq 25$, $-19 \leq k \leq 12$, $-26 \leq l \leq 25$
Reflections collected	37159
Independent reflections	12912 [R_{int} = 0.0940, R_{sigma} = 0.1233]
Data/restraints/parameters	12912/118/923
Goodness-of-fit on F^2	0.896
Final R indexes [I>=2σ (I)]	R_1 = 0.0510, wR_2 = 0.1007
Final R indexes [all data]	R_1 = 0.1411, wR_2 = 0.1262
Largest diff. peak/hole / e Å^{-3}	0.33/-0.28
