# Supplementary material for: Highly Diastereoselective Synthesis of Tetrahydroquinoline Derivatives via [4 + 2] Annulation of Ortho-Tosylaminophenyl-Substituted Para-Quinone Methides and Cyanoalkenes
Source: Front Chem. 2021 Nov 3;9:764866. doi: 10.3389/fchem.2021.764866 (PMC8595915; doi:10.3389/fchem.2021.764866)

checkCIF/PLATON report

You have not supplied any structure factors. As a result the full set of tests cannot be run.

THIS REPORT IS FOR GUIDANCE ONLY. IF USED AS PART OF A REVIEW PROCEDURE FOR PUBLICATION, IT SHOULD NOT REPLACE THE EXPERTISE OF AN EXPERIENCED CRYSTALLOGRAPHIC REFEREE.

No syntax errors found. CIF dictionary Interpreting this report

Datablock: exp_12095_sq

```
Bond precision:  C-C = 0.0065 A          Wavelength=1.54184

Cell:           a=11.0596(10)    b=34.5548(16)       c=11.2811(10)
                alpha=90         beta=119.369(12)    gamma=90
Temperature:    293 K
```

	Calculated	Reported
Volume	3757.1(7)	3757.1(6)
Space group	P 21/c	P 1 21/c 1
Hall group	-P 2ybc	-P 2ybc
Moiety formula	C38 H39 N3 O3 S [+ solvent]	C38 H39 N3 O3 S
Sum formula	C38 H39 N3 O3 S [+ solvent]	C38 H39 N3 O3 S
Mr	617.78	617.78
Dx,g cm-3	1.092	1.092
Z	4	4
Mu (mm-1)	1.048	1.048
F000	1312.0	1312.0
F000’	1316.85	
h,k,lmax	13,42,13	13,42,13
Nref	7351	7193
Tmin,Tmax	0.828,0.882	0.486,1.000
Tmin’	0.811	

```
Correction method= # Reported T Limits: Tmin=0.486 Tmax=1.000
AbsCorr = MULTI-SCAN

Data completeness= 0.979          Theta(max)= 71.809

R(reflections)= 0.0745( 4392)     wR2(reflections)= 0.2781( 7193)

S = 1.109                  Npar= 415
```

The following ALERTS were generated. Each ALERT has the format
test-name_ALERT_alert-type_alert-level.
Click on the hyperlinks for more details of the test.

Alert level B

```
PLAT414_ALERT_2_B Short Intra D-H..H-X       H002     ..H01F             1.84 Ang.
                                                       x,y,z  =      1_555 Check
```

Alert level C

```
PLAT084_ALERT_3_C High wR2 Value (i.e. > 0.25) ...................       0.28 Report
PLAT242_ALERT_2_C Low    'MainMol' Ueq as Compared to Neighbors of       C00I Check
PLAT242_ALERT_2_C Low    'MainMol' Ueq as Compared to Neighbors of       C00K Check
PLAT242_ALERT_2_C Low    'MainMol' Ueq as Compared to Neighbors of       C00L Check
PLAT340_ALERT_3_C Low Bond Precision on  C-C Bonds ...............    0.00648 Ang.
```

Alert level G

```
PLAT007_ALERT_5_G Number of Unrefined Donor-H Atoms ..............          1 Report
PLAT072_ALERT_2_G SHELXL First  Parameter in WGHT  Unusually Large       0.14 Report
PLAT128_ALERT_4_G Alternate Setting for Input Space Group  P21/c        P21/n Note
PLAT199_ALERT_1_G Reported _cell_measurement_temperature ..... (K)        293 Check
PLAT200_ALERT_1_G Reported   _diffrn_ambient_temperature ..... (K)        293 Check
PLAT605_ALERT_4_G Largest Solvent Accessible VOID in the Structure        156 A**3
PLAT720_ALERT_4_G Number of Unusual/Non-Standard Labels ..........         84 Note
PLAT793_ALERT_4_G Model has Chirality at C00A        (Centro SPGR)          R Verify
PLAT793_ALERT_4_G Model has Chirality at C00E        (Centro SPGR)          S Verify
PLAT869_ALERT_4_G ALERTS Related to the Use of SQUEEZE Suppressed           ! Info
PLAT883_ALERT_1_G No Info/Value for _atom_sites_solution_primary .     Please Do !
PLAT941_ALERT_3_G Average HKL Measurement Multiplicity ...........        3.4 Low
```

---

   0 **ALERT level A** = Most likely a serious problem - resolve or explain
   1 **ALERT level B** = A potentially serious problem, consider carefully
   5 **ALERT level C** = Check. Ensure it is not caused by an omission or oversight
  12 **ALERT level G** = General information/check it is not something unexpected

   3 ALERT type 1 CIF construction/syntax error, inconsistent or missing data
   5 ALERT type 2 Indicator that the structure model may be wrong or deficient
   3 ALERT type 3 Indicator that the structure quality may be low
   6 ALERT type 4 Improvement, methodology, query or suggestion
   1 ALERT type 5 Informative message, check

It is advisable to attempt to resolve as many as possible of the alerts in all categories. Often the minor alerts point to easily fixed oversights, errors and omissions in your CIF or refinement strategy, so attention to these fine details can be worthwhile. In order to resolve some of the more serious problems it may be necessary to carry out additional measurements or structure refinements. However, the purpose of your study may justify the reported deviations and the more serious of these should normally be commented upon in the discussion or experimental section of a paper or in the "special_details" fields of the CIF. checkCIF was carefully designed to identify outliers and unusual parameters, but every test has its limitations and alerts that are not important in a particular case may appear. Conversely, the absence of alerts does not guarantee there are no aspects of the results needing attention. It is up to the individual to critically assess their own results and, if necessary, seek expert advice.

Publication of your CIF in IUCr journals

A basic structural check has been run on your CIF. These basic checks will be run on all CIFs submitted for publication in IUCr journals (*Acta Crystallographica*, *Journal of Applied Crystallography*, *Journal of Synchrotron Radiation*); however, if you intend to submit to *Acta Crystallographica Section C* or *E* or *IUCrData*, you should make sure that full publication checks are run on the final version of your CIF prior to submission.

Publication of your CIF in other journals

Please refer to the *Notes for Authors* of the relevant journal for any special instructions relating to CIF submission.

PLATON version of 22/03/2021; check.def file version of 19/03/2021

Datablock exp_12095_sq - ellipsoid plot

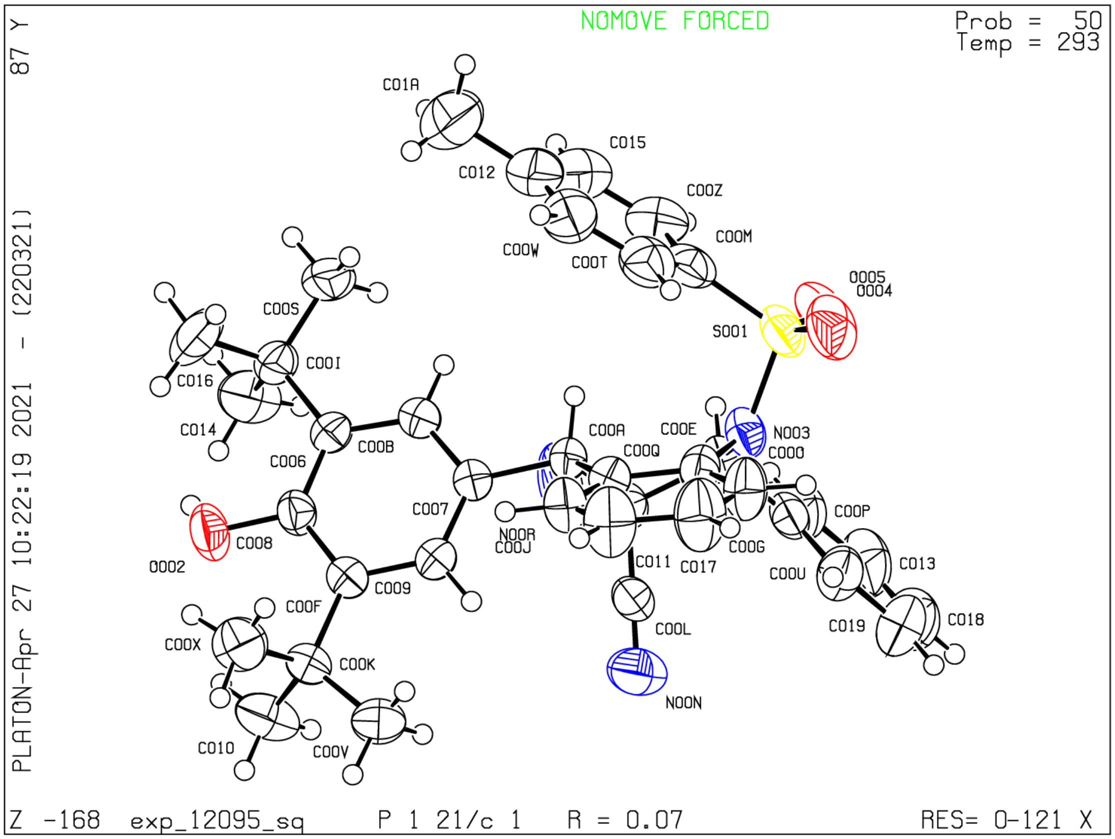

Supplement: Supplementary file 3 [file DataSheet1.PDF]
